# New species and additional records of saprobic microfungi associated with *Macadamia* in China and Thailand and their lignocellulitic enzyme production

**DOI:** 10.3897/mycokeys.141.209886

**Published:** 2026-10-07

**Authors:** Xian Zhang, Samantha C. Karunarathna, Saowaluck Tibpromma, Chanokned Senwanna, Jaturong Kumla, Tian-Ye Du, Abdallah M. Elgorban, Alanoud T. Alfagham, Faten Zubair Filimban, Dong-Qin Dai, Nakarin Suwannarach

**Affiliations:** 1 Department of Biology, Faculty of Science, Chiang Mai University, Chiang Mai 50200, Thailand Department of Biology, Faculty of Science, Chiang Mai University Chiang Mai Thailand; 2 Center for Yunnan Plateau Biological Resources Protection and Utilization & Yunnan International Joint Laboratory of Fungal Sustainable Utilization in South and Southeast Asia, College of Biology and Food Engineering, Qujing Normal University, Qujing 655099, China Center for Yunnan Plateau Biological Resources Protection and Utilization & Yunnan International Joint Laboratory of Fungal Sustainable Utilization in South and Southeast Asia, College of Biology and Food Engineering, Qujing Normal University Qujing China; 3 Key Laboratory of Yunnan Provincial Department of Education of the Deep-Time Evolution on Biodiversity from the Origin of the Pearl River, Qujing Normal University, Qujing 655011, China Key Laboratory of Yunnan Provincial Department of Education of the Deep-Time Evolution on Biodiversity from the Origin of the Pearl River, Qujing Normal University Qujing China; 4 Center of Excellence in Microbial Diversity and Sustainable Utilization, Chiang Mai University, Chiang Mai 50200, Thailand Center of Excellence in Microbial Diversity and Sustainable Utilization, Chiang Mai University Chiang Mai Thailand; 5 Office of Research Administration, Chiang Mai University, Chiang Mai 50200, Thailand Office of Research Administration, Chiang Mai University Chiang Mai Thailand; 6 Shenzhen Key Laboratory of Microbial Genetic Engineering, College of Life Science and Oceanography, Shenzhen University, Shenzhen 518060, China Shenzhen Key Laboratory of Microbial Genetic Engineering, College of Life Science and Oceanography, Shenzhen University Shenzhen China; 7 Center of Excellence in Biotechnology Research, DSR, King Saud University, Riyadh, 12372, Saudi Arabia Center of Excellence in Biotechnology Research, DSR, King Saud University Riyadh Saudi Arabia; 8 Department of Botany and Microbiology, College of Science, King Saud University, Riyadh 1l45l, Saudi Arabia Department of Botany and Microbiology, College of Science, King Saud University Riyadh Saudi Arabia; 9 Division of Plant Sciences, Department of Biological Sciences, Faculty of Sciences, King Abdulaziz University, Jeddah, Saudi Arabia Division of Plant Sciences, Department of Biological Sciences, Faculty of Sciences, King Abdulaziz University Jeddah Saudi Arabia

**Keywords:** Lignocellulose-degrading enzyme, morphology, new taxa, phylogenetic analysis, saprobes

## Abstract

*Macadamia*, a tropical and subtropical plant, is one of the most popular and nutritious nuts worldwide. Previous studies on macadamia-associated fungi have mainly focused on pathogenic and endophytic species, but the diversity and taxonomy of saprobic fungi associated with *Macadamia* remain poorly characterized. In this study, 18 fungal taxa associated with *Macadamia* spp. were collected from China and Thailand. Through morphological and multilocus phylogenetic analyses, five new species (*Dothiorella lignicola*, *Lasiodiplodia macadamiae*, *Palmiascoma macadamiae*, *Xylochrysis macadamiae*, and *X. yunnanensis*) are introduced, along with 13 previously known species across nine genera that are newly recorded from macadamia. In addition, 20 isolates representing these 18 taxa were evaluated for their potential to produce lignocellulolytic enzymes. Among the four enzymes evaluated, xylanase was the most frequently detected, followed by cellulase and mannanase, whereas laccase production was observed in only a limited number of isolates. These findings expand the known diversity of saprobic microfungi associated with macadamia and highlight their potential ecological roles in lignocellulose decomposition and possible applications in biotechnology.

## Introduction

*Macadamia* F. Muell. belongs to an early-diverging eudicot family and is cultivated as a perennial crop, and its nuts are widely known as “the king of nuts” because of their high nutritional value and economic importance (Wrona et al. 2020; Li et al. 2022; Tao et al. 2022; Zhang et al. 2025c). Beyond the production of edible nuts, macadamia has considerable commercial value because its various components and by-products can be utilized in a wide range of applications, including the production of adsorbents, edible oils, beverages, personal care products, cleaning products, and other value-added food products (Mojapelo et al. 2023; Alasalvar et al. 2025). In the last century, macadamia was found in tropical forest fringes from southeastern Queensland to northeastern New South Wales, Australia (Carpenter et al. 2015; Nock et al. 2019; Akinsanmi and Carvalhais 2020). The genus *Macadamia* includes four species, viz. *M. integrifolia*, *M. jansenii*, *M. ternifolia*, and *M. tetraphylla* (Tao et al. 2022; Zhang et al. 2025c). Among these four species, *M. integrifolia* and *M. tetraphylla* produce edible nuts; however, the other two species (*M. jansenii* and *M. ternifolia*) produce small and inedible kernels (Prasannath et al. 2021; Zhang et al. 2025c). *Macadamia integrifolia* and hybrid cultivars of *M. integrifolia* and *M. tetraphylla* are widely planted worldwide (Hardner 2016; Nock et al. 2019). Currently, macadamia nuts are mainly grown in Australia, Brazil, China, Kenya, Malawi, New Zealand, South Africa, South America, Thailand, and the United States (Nock et al. 2019; Twiddy et al. 2024). In China, macadamia was first introduced and planted in the 1970s (Shuai et al. 2022; Tao et al. 2022). In recent years, China has accounted for more than 60% of the world’s cultivated area, with the main plantations located in Guangxi, Guangdong, and Yunnan provinces (Tao et al. 2022; Gong et al. 2024; Zhang et al. 2025b). Among them, Yunnan Province has the largest macadamia plantation area, accounting for 88%, and its harvested area accounts for more than 90% of China’s total (Gong et al. 2024). Meanwhile, Yunnan also has the world’s largest cultivated area (Gong et al. 2024). In Thailand, macadamia was introduced for experimental cultivation in 1953 and was subsequently cultivated in northern Thailand, particularly in Chiang Mai Province. In recent years, macadamia cultivation has expanded considerably in northern Thailand and has become an increasingly important cash crop, driven by growing domestic consumption and its potential for international export (Nilpanich et al. 2025).

Macadamia-growing regions provide favorable habitats for diverse organisms, including fungi, which are among the most prevalent microorganisms associated with macadamia from cultivation through postharvest handling and processing (Wrona et al. 2020; Twiddy et al. 2024; Zhang et al. 2025b). Microfungi, including saprobic, endophytic, and pathogenic forms, are widely distributed worldwide. Saprobic fungi play a very important role as decomposers; they contribute to the carbon, nitrogen, and phosphorus cycles by decomposing organic matter (Treseder and Lennon 2015; Tian et al. 2024a; Du et al. 2025b). Several publications have reported fungi associated with *Macadamia* spp. (Prasannath et al. 2020, 2021; Li et al. 2022; Tao et al. 2022). For example, *Trichoderma hamatum* has been isolated as an endophyte from healthy macadamia trees (Lewis et al. 1996; Rabeendran et al. 2006; Mao et al. 2020; Li et al. 2022). In contrast, numerous pathogenic fungi, predominantly belonging to the genera *Colletotrichum*, *Diaporthe*, *Neopestalotiopsis*, and *Pseudocercospora*, have been reported to cause diseases affecting various parts of macadamia plants, including husks, flowers, leaves, and fruits (Sutton 1991; Denman et al. 2003; Miles et al. 2009; Akinsanmi et al. 2016a, 2016b; Liddle et al. 2018; Jeff-Ego and Akinsanmi 2019; Tan et al. 2019; Prasannath et al. 2020, 2021; Wrona et al. 2020; Li et al. 2022; Mohankumar et al. 2022; Twiddy et al. 2024). However, saprobic fungi associated with *Macadamia* spp. remain poorly documented compared with endophytic and pathogenic fungi. Only a limited number of saprobic species, including *Fusarium polyphialidicum*, *Melomastia puerensis*, *Neopalmiascoma macadamiae*, and *Phaeoacremonium chiangmaiense*, have been reported (Ruiz et al. 1997; Zhang et al. 2003; Prasannath et al. 2021; Zhang et al. 2024, 2025b). The fungal life mode of several species has not been reported (Prasannath et al. 2021; CABI 2026).

Saprobic microfungi, particularly those associated with woody substrates, play an essential role in organic matter decomposition and nutrient cycling in ecosystems (Sánchez 2009; Sharma and Arora 2015; Eliopoulos et al. 2025). By secreting extracellular hydrolytic and oxidative lignocellulolytic enzymes, including cellulases, xylanases, laccases, mannanases, manganese peroxidases, and lignin peroxidases, these fungi contribute to the degradation of complex organic compounds, particularly lignocellulosic materials, thereby facilitating nutrient release (Manavalan et al. 2015; Benatti and Polizeli 2023). In addition, saprobic microfungi are of considerable biotechnological interest because they produce lignocellulolytic enzymes with diverse applications in biomass conversion, biofuel production, the food and feed industries, pulp and paper processing, textile production, and environmental bioremediation (Chukwuma et al. 2020; Kumla et al. 2020). However, the diversity and ecological roles of saprobic fungi associated with *Macadamia* spp. remain insufficiently explored, particularly in tropical and subtropical regions. Therefore, this study aimed to investigate saprobic microfungi associated with macadamia in China and Thailand using morphological and multilocus phylogenetic analyses. Eighteen fungal taxa were obtained, including five new species and 13 species newly recorded from macadamia (Table 1). Moreover, all obtained microfungal strains were evaluated for their ability to produce lignocellulolytic enzymes.

**Table 1.** Saprobic microfungi obtained in this study.

**Phylum: *Ascomycota* Caval.-Sm**	
**Subphylum: *Pezizomycotina* O.E. Erikss. & Winka**	
Class: ***Sordariomycetes*** O.E. Erikss. & Winka	
Subclass: ***Diaporthomycetidae*** Senan., Maharachch. & K.D. Hyde	
Family: ***Woswasiaceae*** Huang Zhang, K.D. Hyde & Maharachch	
Genus: ***Xylochrysis*** Réblová	
1.	***Xylochrysis macadamiae*** X. Zhang, Tibpromma & Karun., sp. nov.
2.	***Xylochrysis yunnanensis*** X. Zhang, Tibpromma & Karun., sp. nov.
Subclass: ***Pleosporomycetidae*** C.L. Schoch, Spatafora, Crous & Shoemaker	
Order: ***Sporidesmiales*** Crous	
Family: ***Sporidesmiaceae*** Summa veg. Scand., Sectio Post	
Genus: ***Sporidesmium*** Link	
3.	***Sporidesmium tropicale*** M.B. Elli (new host record)
Class: ***Dothideomycetes*** O.E. Erikss. & Winka	
Subclass: ***Pleosporomycetidae*** C.L. Schoch, Spatafora, Crous & Shoemaker	
Order: ***Botryosphaeriales*** C.L. Schoch, Crous & Shoemaker	
Family: ***Botryosphaeriaceae*** Theiss. & Syd	
Genus: ***Dothiorella*** Sacc.	
4.	***Dothiorella lignicola*** X. Zhang & N. Suwannar., sp. nov.
Genus: ***Lasiodiplodia*** Ellis & Everh.	
5.	***Lasiodiplodia macadamiae*** X. Zhang, Tibpromma & Karun., sp. nov.
6.	***Lasiodiplodia ponkanicola*** X.E. Xiao, Crous & H.Y. Li (new host record and geographic record in Thailand)
7.	***Lasiodiplodia endophytica*** N.I. de Silva, A.J.L. Phillips & K.D. Hyde (new host record and geographic record in Thailand)
Order: ***Pleosporales*** Luttr. ex M.E. Barr	
Family: ***Bambusicolaceae*** D.Q. Dai & K.D. Hyde	
Genus: ***Palmiascoma*** Phookamsak & K.D. Hyde	
8.	***Palmiascoma macadamiae*** X. Zhang, Tibpromma & Karun., sp. nov.
9.	***Palmiascoma chiangraiense*** N. Afshari, Lumyong & K.D. Hyde (new host record and geographic record in China)
Genus: ***Crassiparies*** Matsumura, K. Hiray & Kaz.	
10.	***Crassiparies quadrisporus*** M. Matsum., K. Hiray. & Kaz. (new host record and geographic record in China)
Family: ***Didymosphaeriaceae*** Munk	
Genus: ***Chromolaenicola*** Mapook & K.D. Hyde	
11.	***Chromolaenicola ananasi*** X.G. Tian, K.D. Hyde & Tibpromma (new host record)
Genus: ***Montagnula*** Berl.	
12.	***Montagnula donacina*** (Niessl) Wanas., E.B.G. Jones & K.D. Hyde (new host record)
13.	***Montagnula thailandica*** Mapook & K.D. Hyde (new host record)
Family: ***Macrodiplodiopsidaceae*** Voglmayr, Jaklitsch & Crous	
Genus: ***Pseudochaetosphaeronema*** Punith.	
14.	***Pseudochaetosphaeronema chiangraiense*** Wijes., Boonmee & K.D. Hyde (new host record and geographic record from China)
15.	***Pseudochaetosphaeronema puerense*** R.F. Xu, K.D. Hyde & Tibpromma (new host record)
16.	***Pseudochaetosphaeronema siamense*** Jayasiri, E.B.G. Jones & K.D. Hyde (new host record and geographic record from China)
Family: ***Massarinaceae*** Munk	
Genus: ***Helminthosporium*** Link	
17.	***Helminthosporium puerense*** L. Lu & Tibpromma (new host record)
18.	***Helminthosporium nanjingense*** Meng Zhang, Xiao J. Wang & H.Y. Wu (new host record)

## Materials and methods

### Sample collection, fungal isolation, and morphological study

Dead and decaying twigs and stems of *Macadamia* sp. bearing fungal fruiting bodies were collected from macadamia plantations in Yunnan Province, China, and Chiang Mai Province, Thailand, during 2023–2024. Specimens were placed in separate plastic bags and transported to the mycology laboratories at Chiang Mai University, Thailand, and Qujing Normal University, China, respectively. Each bag was labeled with collection details, including altitude, date, site, and global positioning system (GPS) coordinates (Rathnayaka et al. 2025). The fruiting body features of the specimens were observed using an Olympus LEICA S8 APO optical microscope (Tokyo, Japan) and a Nikon SMZ800N stereomicroscope. Micromorphological structures were observed, and photographs were taken using an OLYMPUS BX53 compound microscope (Olympus, Tokyo, Japan) connected to an OLYMPUS DP74 digital camera (Olympus, Tokyo, Japan) or using a Nikon DS-Ri2 camera connected to a Nikon ECLIPSE Ni compound microscope (Tokyo, Japan). Adobe Photoshop CS3 Extended version 10.0 (Adobe Systems, USA) was used to prepare photographic plates, and Tarosoft (R) Image Frame Work (v. 0.9.0.7) was used to measure the microstructures. Pure fungal isolates were obtained by single-spore isolation following the method described by Senanayake et al. (2020). Germinated spores on potato dextrose agar (PDA) were transferred to new PDA plates, and culture characteristics were observed after 2–4 weeks of incubation at 25–28 °C in an incubator.

Specimens were deposited in the Herbarium of Guizhou Medical University (**GMB-W**) and the Herbarium of the Department of Biology (**CMUB**), Faculty of Science, Chiang Mai University, Thailand. Living cultures were deposited in the Guizhou Medical University Culture Collection (**GMBCC**), China, and the Sustainable Development of Biological Resources (**SDBR-CMU**), Faculty of Science, Chiang Mai University, Thailand. MycoBank numbers (MB) were registered as outlined in MycoBank (2026).

### DNA extraction, PCR amplification, and sequencing

Molecular studies were carried out according to the method described by Dissanayake et al. (2020). Genomic DNA was extracted from two-week-old mycelium cultivated on PDA using the DNA Extraction Kit-BSC14S1 (BioFlux, Hangzhou, P.R. China) and the FavorPrep Tissue Genomic DNA Extraction Mini Kit (FAVORGEN Biotech Corp., Taiwan, P.R. China) according to the manufacturers’ instructions. Polymerase chain reactions (PCR) were performed to amplify partial gene regions using the primers and conditions listed in Table 2. The 25 μL PCR mixtures contained the following components: ddH_2_O (9.5 μL), 2× Master Mix (12.5 μL) (Biotek Corporation, Beijing, China), DNA template (1 μL), and each forward and reverse primer (1 μL) (Tibpromma et al. 2018; Senwanna et al. 2023; Zhang et al. 2025b). PCR products were checked on 1% agarose gels and sent to Sangon Biotech (Shanghai Co., Ltd.), China, and 1^st^ BASE Company (Kembangan, Malaysia).

**Table 2.** Loci, primers, and PCR conditions used in this study.

**Loci**	**Primers**	**PCR conditions**	**References**
ITS	ITS4/ITS5	95 °C 3 min, (94 °C 30 s, 55 °C 50 s, 72 °C 90 s) × 35 cycles, 72 °C 10 min	White et al. (1990)
nrLSU	LR5/LR0R	95 °C 3 min, (94 °C 30 s, 55 °C 50 s, 72 °C 90 s) × 35 cycles, 72 °C 10 min	Vilgalys and Hester (1990)
nrSSU	NS1/NS4	95 °C 3 min, (94 °C 30 s, 55 °C 50 s, 72 °C 90 s) × 35 cycles, 72 °C 10 min	White et al. (1990)
*tef1-α*	983F/2218R	95 °C 3 min, (95 °C 30 s, 55 °C 50 s, 72 °C 90 s) × 35 cycles, 72 °C 10 min	Rehner and Buckley (2005)
	728F/986R	95 °C 3 min, (95 °C 30 s, 55 °C 30 s, 72 °C 60 s) × 40 cycles, 72 °C 10 min	Carbone and Kohn (1999)
*tub2*	T1/Bt2b	94 °C 4 min, (94 °C 40 s, 52 °C 30 s, 72 °C 60 s) × 35 cycles, 72 °C 10 min	Glass and Donaldson (1995); O’Donnell and Cigelnik (1997)
*rpb2*	fRPB2-5F/fRPB2-7cR	94 °C 5 min, (94 °C 45 s, 55 °C 120 s, 72 °C 180 s) × 40 cycles, 72 °C 10 min	Liu et al. (1999)

### Phylogenetic analyses

All sequences were assembled using Geneious 9.1.8 (https://www.geneious.com), and the newly generated sequences (Table 3) were submitted to BLASTn to search nucleotide databases and identify the most similar taxa. The sequences used in the phylogenetic analyses were downloaded from GenBank, as described in recent publications. Multiple sequence alignments were conducted using the online program MAFFT v. 7.110 (https://mafft.cbrc.jp/alignment/server/). TrimAl.v1.2rev59 (http://trimal.cgenomics.org) was used to automatically modify the alignments (Capella-Gutiérrez et al. 2009). All sequence data were combined using SequenceMatrix v. 1.7.8 (Vaidya et al. 2011). AliView v. 1.28 was used to convert the FASTA files to NEXUS files (Larsson 2014). Multilocus phylogenetic analyses of the combined sequences were conducted using randomized accelerated maximum likelihood (RAxML) and Bayesian inference (BI).

**Table 3:** . Voucher specimen data and GenBank accession numbers for sequences generated from this study. “T” isolates represent ex-type, “-” indicates no sequence data in GenBank.

**Species name**	**Strains**	**Host**	**Location**	**GenBank Accession No**.					
				**ITS**	**nrLSU**	**nrSSU**	***tef*1-*α***	***tub*2**	***rpb*2**
*Xylochrysis macadamiae*	GMBCC 2440T	*Macadamia* sp.	Dehong, Yunnan, China	PX921755	PX921759	PX921934	PZ224681	-	PZ210148
*Xylochrysis macadamiae*	GMBCC 2441	*Macadamia* sp.	Dehong, Yunnan, China	PX921756	PX921760	PX921935	PZ224682	-	PZ210149
*Xylochrysis yunnanensis*	GMBCC 2442T	*Macadamia* sp.	Dehong, Yunnan, China	PX921757	PX921761	-	PZ224679	-	PZ210150
*Xylochrysis yunnanensis*	GMBCC 2443	*Macadamia* sp.	Dehong, Yunnan, China	PX921758	PX921762	-	PZ224680	-	PZ210151
*Sporidesmium tropicale*	GMBCC 2444	*Macadamia* sp.	Pu’er, Yunnan, China	PZ147879	PZ147883	-	-	-	PZ150683
*Sporidesmium tropicale*	GMBCC 2445	*Macadamia* sp.	Pu’er, Yunnan, China	PZ147880	PZ147884	-	-	-	PZ150684
*Dothiorella lignicola*	SDBR-CMU873T	*Macadamia* sp.	Doi Saket, Chiang Mai, Thailand	PZ162690	-	-	PZ167135	PZ167137	-
*Dothiorella lignicola*	SDBR-CMU874	*Macadamia* sp.	Doi Saket, Chiang Mai, Thailand	PZ162691	-	-	PZ167136	PZ167138	-
*Lasiodiplodia macadamiae*	GMBCC 2446T	*Macadamia* sp.	Pu’er, Yunnan, China	PZ158349	-	-	PZ150673	PZ150677	-
*Lasiodiplodia macadamiae*	GMBCC 2447	*Macadamia* sp.	Pu’er, Yunnan, China	PZ158350	-	-	PZ150674	PZ150678	-
*Lasiodiplodia ponkanicola*	CMUB 40122	*Macadamia* sp.	Samoeng Tai, Chiang Mai, Thailand	PZ158351	-	-	PZ150675	PZ150679	-
*Lasiodiplodia ponkanicola*	SDBR-CMU875	*Macadamia* sp.	Samoeng Tai, Chiang Mai, Thailand	PZ158352	-	-	PZ150676	PZ150680	-
*Lasiodiplodia endophytica*	CMUB 40123	*Macadamia* sp.	Samoeng Tai, Chiang Mai, Thailand	PZ158353	-	-	QB037503	PZ150681	-
*Lasiodiplodia endophytica*	SDBR-CMU876	*Macadamia* sp.	Samoeng Tai, Chiang Mai, Thailand	PZ158354	-	-	QB037504	PZ150682	-
*Palmiascoma macadamiae*	GMBCC 2448T	*Macadamia* sp.	Dehong, Yunnan, China	PX929868	PX929872	PX929876	-	-	PX931730
*Palmiascoma macadamiae*	GMBCC 2449	*Macadamia* sp.	Dehong, Yunnan, China	PX929869	PX929873	PX929877	-	-	PX931731
*Palmiascoma chiangraiense*	GMBCC 2450	*Macadamia* sp.	Pu’er, Yunnan, China	PX929870	PX929874	PX929878	-	-	PX931728
*Palmiascoma chiangraiense*	GMBCC 2451	*Macadamia* sp.	Pu’er, Yunnan, China	PX929871	PX929875	PX929879	-	-	PX931729
*Crassiparies quadrisporus*	GMBCC 2452	*Macadamia* sp.	Dehong, Yunnan, China	PX931083	PX931114	PX931117	PX931734	-	PX931732
*Crassiparies quadrisporus*	GMBCC 2453	*Macadamia* sp.	Dehong, Yunnan, China	PX931084	PX931115	PX931118	PX931735	-	PX931733
*Chromolaenicola ananasi*	CMUB 40120	*Macadamia* sp.	Doi Saket, Chiang Mai, Thailand	PZ151946	PZ151950	PZ151952	PZ210152	-	-
*Chromolaenicola ananasi*	SDBR-CMU872	*Macadamia* sp.	Doi Saket, Chiang Mai, Thailand	PZ151947	PZ151951	PZ151953	PZ210153	-	-
*Montagnula donacina*	GMBCC 2455	*Macadamia* sp.	Honghe, Yunnan, China	PX945742	PX945744	PX945756	PX957010	-	-
*Montagnula thailandica*	GMBCC 2454	*Macadamia* sp.	Honghe, Yunnan, China	PX945741	PX945743	PX945755	PX957009	-	-
*Pseudochaetosphaeronema chiangraiense*	GMBCC 2456	*Macadamia* sp.	Dehong, Yunnan, China	PX946142	PX946148	PX946154	PX957003	-	-
*Pseudochaetosphaeronema chiangraiense*	GMBCC 2457	*Macadamia* sp.	Dehong, Yunnan, China	PX946143	PX946149	PX946155	PX957004	-	-
*Pseudochaetosphaeronema puerense*	GMBCC 2458	*Macadamia* sp.	Dehong, Yunnan, China	PX946144	PX946150	PX946156	PX957005	-	-
*Pseudochaetosphaeronema puerense*	GMBCC 2459	*Macadamia* sp.	Dehong, Yunnan, China	PX946145	PX946151	PX946157	PX957006	-	-
*Pseudochaetosphaeronema siamense*	GMBCC 2460	*Macadamia* sp.	Dehong, Yunnan, China	PX946146	PX946152	PX946158	PX957007	-	-
*Pseudochaetosphaeronema siamense*	GMBCC 2461	*Macadamia* sp.	Dehong, Yunnan, China	PX946147	PX946153	PX946159	PX957008	-	-
*Helminthosporium puerense*	GMBCC 2462	*Macadamia* sp.	Pu’er, Yunnan, China	PX946836	PX946845	PX946841	PZ150669	-	PZ150665
*Helminthosporium puerense*	GMBCC 2464	*Macadamia* sp.	Pu’er, Yunnan, China	PX946837	PX946846	PX946842	PZ150670	-	PZ150666
*Helminthosporium nanjingense*	GMBCC 2466	*Macadamia* sp.	Pu’er, Yunnan, China	PX946838	PX946847	PX946843	PZ150671	-	PZ150667
*Helminthosporium nanjingense*	GMBCC 2467	*Macadamia* sp.	Pu’er, Yunnan, China	PX946839	PX946848	PX946844	PZ150672	-	PZ150668

Maximum likelihood analyses were conducted on the online CIPRES Science Gateway portal (https://www.phylo.org/portal2/login!input.action; Miller et al. 2010) using the RAxML-HPC2 on XSEDE (8.2.12) tool with 1,000 bootstrap (BS) replicates (Stamatakis 2006, 2014; Stamatakis et al. 2008) and IQ-TREE v. 3.1.4 (https://www.hiv.lanl.gov/content/sequence/IQTREE/iqtree.html) (Nguyen et al. 2015). BI was performed using the MrBayes on XSEDE (3.2.7a) tool (Ronquist et al. 2012) and MrBayes v. 3.2.6 (Ronquist et al. 2012), and the best models of evolution were estimated using MrModelTest v. 2.3 (Nylander et al. 2008). Six simultaneous Markov chains were run for 2,000,000–100,000,000 generations (depending on the fungal genera), and trees were sampled every 100 generations (Lu et al. 2025b). The run was automatically terminated when the standard deviation of split frequencies dropped below 0.01, with a 25% burn-in. The phylogenetic trees were visualized using FigTree v. 1.4.2 (Rambaut 2012) and further edited in Microsoft PowerPoint 2021 and Adobe Photoshop CS3 Extended version 22.0.0 (Adobe Systems, California, USA). Maximum likelihood bootstrap support (MLBS) ≥ 60% and Bayesian posterior probabilities (BYPP) ≥ 0.90 are shown above the nodes.

### Evaluation of lignocellulolytic enzyme production

The fungal isolates obtained in this study were used to investigate lignocellulolytic enzyme production using agar plate assays. The production of four lignocellulolytic enzymes, namely cellulase, xylanase, mannanase, and laccase, was investigated following the methods described in previous studies (Kasana et al. 2008; Kalim and Ali 2016). Carboxymethyl cellulose (CMC) agar (10 g/L CMC, 2 g/L NaNO_3_, 1 g/L K_2_HPO_4_, 0.5 g/L MgSO_4_, 0.5 g/L KCl, 0.2 g/L peptone, 17 g/L agar, pH 5.5), xylan agar (10 g/L xylan, 3 g/L yeast extract, 1.5 g/L peptone, 3.5 g/L NaCl, 1 g/L NaNO_3_, 1 g/L KH_2_PO_4_, 0.3 g/L MgSO_4_, 20 g/L agar, pH 5.5), locust bean gum (LBG) agar (10 g/L LBG, 2 g/L NaNO_3_, 1 g/L K_2_HPO_4_, 0.5 g/L MgSO_4_, 0.5 g/L KCl, 0.2 g/L peptone, 17 g/L agar, pH 5.5), and guaiacol agar (0.02% guaiacol, 2 g/L NaNO_3_, 1 g/L K_2_HPO_4_, 0.5 g/L MgSO_4_, 0.5 g/L KCl, 0.2 g/L peptone, 17 g/L agar, pH 5.5) were used to screen for cellulase, xylanase, mannanase, and laccase production, respectively. A 5-mm-diameter mycelial plug taken from a 5-day-old PDA culture of each fungal isolate was inoculated onto the center of each tested agar medium. After 1 week of incubation at 30 °C, the colony diameter was measured. The plates were then flooded with 0.1% Congo red solution for 1 min and washed three times with 15 mL of 1 M NaCl for 15 min each. The diameter of the clear halo zone surrounding the fungal colony was measured as an indicator of positive cellulase, xylanase, and mannanase production (Kasana et al. 2008; Kalim and Ali 2016; Nirmalasari et al. 2022). In contrast, the appearance of a brownish-red zone surrounding the fungal colony on guaiacol agar was used as an indicator of positive laccase production (Yuliana et al. 2024). The production of all four lignocellulolytic enzymes was evaluated using the enzyme potency index (EPI), calculated as the difference between the halo zone diameter or brownish-red zone diameter and the colony diameter, divided by the colony diameter (Sánchez-Corzo et al. 2021; Luong et al. 2025). All experiments were performed in triplicate.

### Statistical analysis

Statistical analyses were performed using one-way analysis of variance (ANOVA) with SPSS Statistics version 27.0.1 (IBM Corp., Armonk, NY, USA). Differences among treatment means were considered statistically significant at *p* ≤ 0.05 and were compared using Duncan’s multiple range test.

## Results

Based on molecular and morphological analyses, five new species and 13 new records of saprobic microfungi associated with macadamia in China and Thailand were identified. Multilocus phylogenetic analyses were conducted to determine the placement of species within the appropriate orders, families, and genera. Full descriptions and photographic plates illustrating the morphological characteristics were provided for all new species. For new records, descriptions and photographic plates were provided only for taxa exhibiting additional morphological characteristics compared with those reported in previous studies.

### Phylogenetic analyses

**Figure 1.**
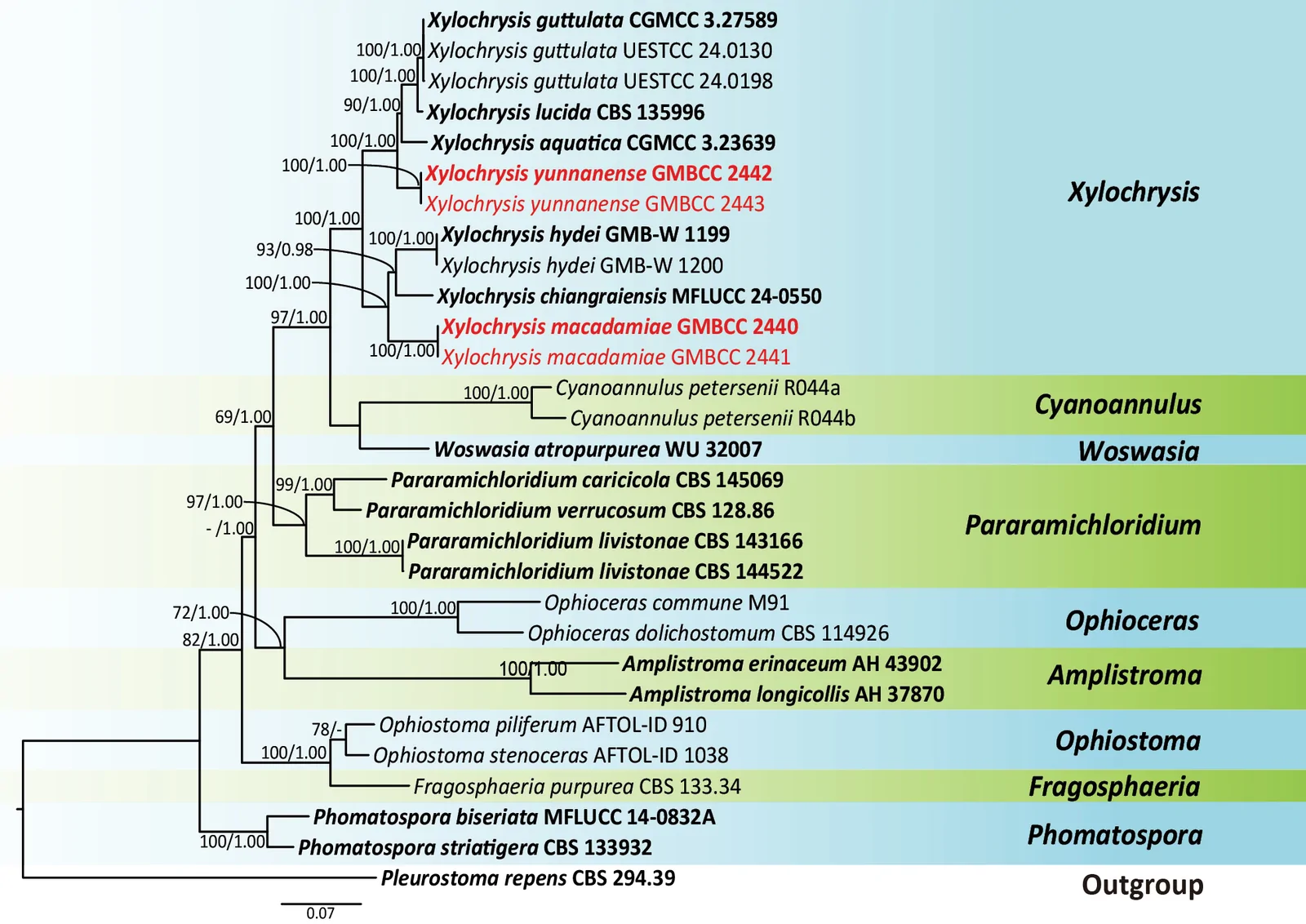
Phylogram generated from maximum likelihood analysis based on a combined dataset of nrLSU, nrSSU, ITS, *tef1-α*, and *rpb2* sequence data. The ML tree topology was similar to the BI topology. ML bootstrap support values of 60% or greater and BYPP values of 0.90 or greater are shown at the nodes. All type strains are shown in bold black, and newly generated sequences are shown in red.

Analysis 1: The phylogenetic analysis of the genus *Xylochrysis* was conducted based on a combined dataset of nrLSU, nrSSU, ITS, *tef1-α*, and *rpb2* sequence data following Xiao and Liu (2025). A total of 28 strains were included in the combined sequence analysis, which comprised 4,380 characters with gaps. *Pleurostoma repens* (CBS 294.39) was used as the outgroup taxon. The ML tree topology was similar to the BI topology. The best-scoring RAxML tree with a final likelihood value of −22144.489 is presented. The matrix contained 1,714 distinct alignment patterns, with 39.82% of characters undetermined or missing. Estimated base frequencies were as follows: A = 0.242119, C = 0.259806, G = 0.279643, T = 0.218433; substitution rates AC = 1.000000, AG = 1.888192, AT = 1.000000, CG = 1.000000, CT = 4.993208, GT = 1.000000; proportion of invariable sites *I* = 0.642117; gamma distribution shape parameter *α* = 0.738000. The ML and BI trees showed identical topologies. Therefore, only the ML tree is presented in Fig. 1. The phylogenetic tree showed that the *Xylochrysis* clade was strongly supported (100% MLBS, 1.00 BYPP) and was clearly separated from the other genera. The four *Xylochrysis* isolates obtained in this study formed two distinct monophyletic clades. The two isolates, GMBCC 2442 and GMBCC 2443, formed a distinct and well-supported lineage (100% MLBS, 1.00 BYPP), representing a new species, *X. yunnanensis*. The two isolates GMBCC 2440 and GMBCC 2441 formed a distinct lineage closely related to *X. hydei* and *X. chiangraiensis* (100% MLBS, 1.00 BYPP) and are introduced herein as a new species, *X. macadamiae*.

**Figure 2.**
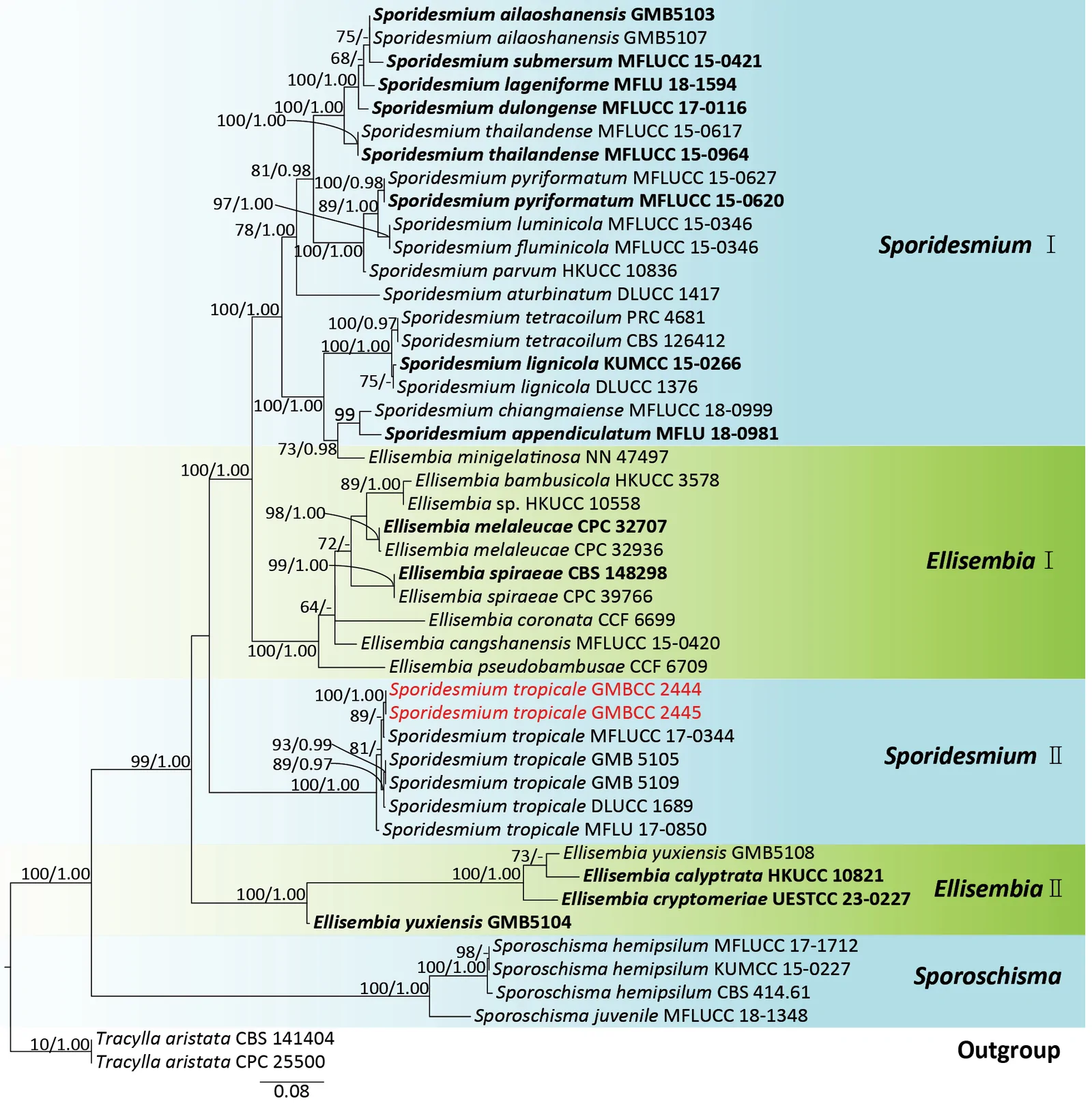
Phylogram generated from maximum likelihood analysis based on a combined dataset of ITS, nrLSU, and *rpb2* sequence data. The ML tree topology was similar to the BI topology. ML bootstrap support values of 60% or greater and BYPP values of 0.90 or greater are shown above the nodes. All type strains are shown in bold black, and newly generated sequences are shown in red.

Analysis 2: The phylogenetic analysis of the species of *Sporidesmium* was conducted based on a combined dataset of ITS, nrLSU, and *rpb2* sequence data following Zhang et al. (2025a). A total of 30 strains were included in the combined sequence analysis, which comprised 3,481 characters with gaps. *Tracylla aristata* (CBS 141404) and *Tracylla aristata* (CPC 25500) were used as the outgroup taxa. The ML tree topology was similar to the BI topology. Therefore, only the ML tree is presented in Fig. 2. The best-scoring RAxML tree with a final likelihood value of −15496.102001 is presented. The matrix contained 1,133 distinct alignment patterns, with 40.82% of characters undetermined or missing. Estimated base frequencies were as follows: A = 0.242664, C = 0.253299, G = 0.292907, T = 0.211130; substitution rates AC = 1.542893, AG = 2.733518, AT = 1.615427, CG = 0.991797, CT = 7.194628, GT = 1.000000; proportion of invariable sites *I* = 0.345021; gamma distribution shape parameter *α* = 0.533110. The phylogenetic tree showed that the two isolates GMBCC 2444 and GMBCC 2445 were nested within *S. tropicale* (GMB 5105, GMB 5109, DLUCC 1689, MFLU 17-0850, MFLUCC 17-0344) with 89% MLBS statistical support. Therefore, these two isolates are identified as *S. tropicale*.

**Figure 3.**
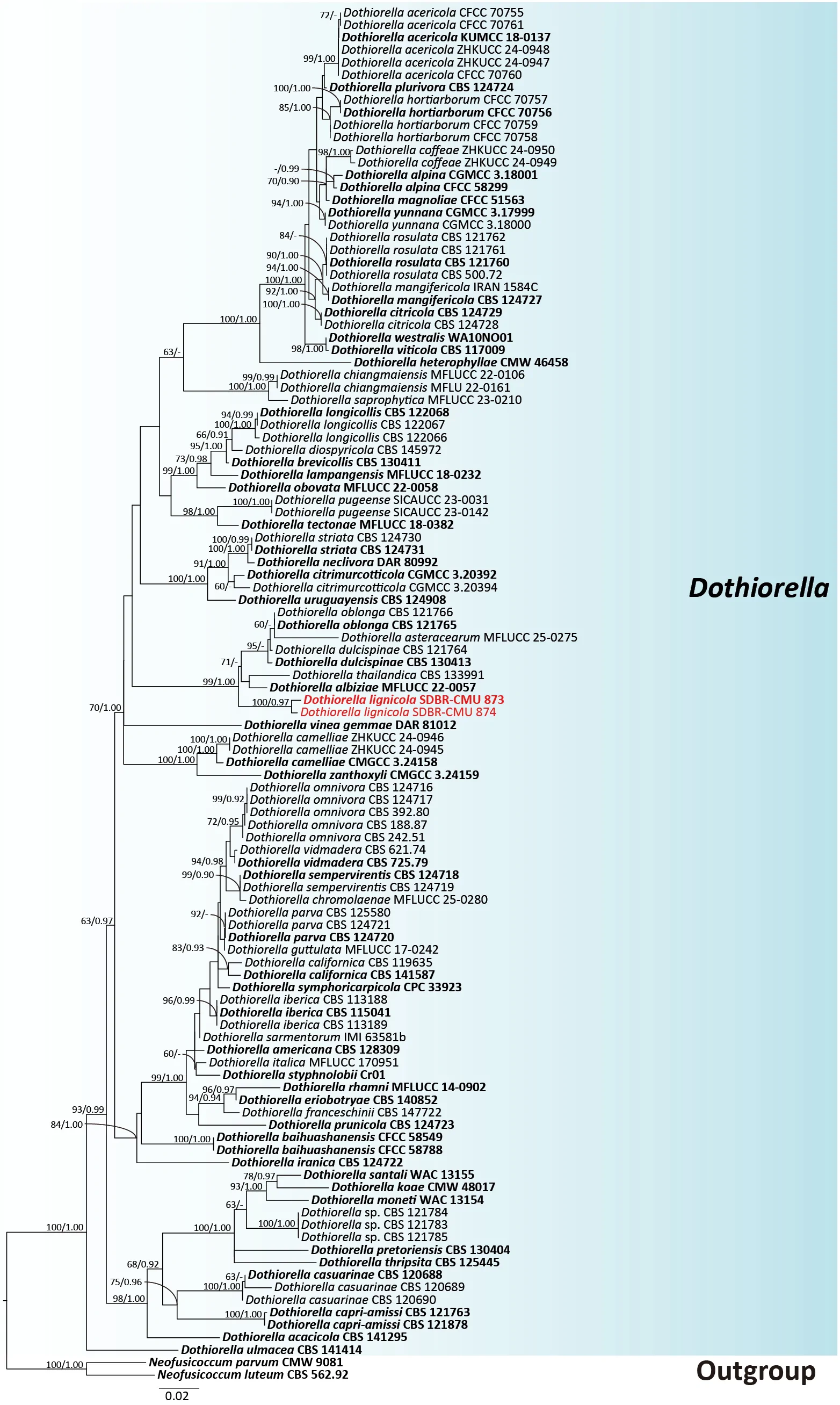
Phylogram generated from maximum likelihood analysis based on a combined dataset of ITS, *tef1-α*, and *rpb2* sequence data. The ML tree topology was similar to the BI topology. ML bootstrap support values of 60% or greater and BYPP values of 0.90 or greater are shown above the nodes. All type strains are shown in bold black, and newly generated sequences are shown in red.

Analysis 3: The phylogenetic analysis of the genus *Dothiorella* was conducted based on a combined dataset of ITS, *tef1-α*, and *rpb2* sequence data following Zhou et al. (2025). A total of 30 strains were included in the combined sequence analysis, which comprised 1,439 characters with gaps. *Neofusicoccum luteum* (CBS 562.92) and *Neofusicoccum parvum* (CMW 9081) were used as the outgroup taxa. The ML tree topology was similar to the BI topology. Therefore, only the ML tree is presented in Fig. 3. The best-scoring RAxML tree with a final likelihood value of −9878.969843 is presented. The matrix contained 745 distinct alignment patterns, with 23.50% of characters undetermined or missing. Estimated base frequencies were as follows: A = 0.209667, C = 0.306778, G = 0.250584, T = 0.232970; substitution rates AC = 1.108033, AG = 2.427500, AT = 1.299719, CG = 1.089085, CT = 4.472302, GT = 1.000000; proportion of invariable sites *I* = 0.464341; gamma distribution shape parameter *α* = 0.665543. The phylogenetic analysis showed that the two strains, SDBR-CMU873 and SDBR-CMU874, clustered within the genus *Dothiorella* and formed a distinct lineage with strong statistical support (100% MLBS, 0.97 BYPP). They were closely related to a clade comprising *D. albiziae* (MFLUCC 22-0057), *D. asteracearum* (MFLUCC 25-0275), *D. dulcispinae* (CBS 121764, CBS 130413), *D. oblonga* (CBS 121765, CBS 121766), and *D. thailandica* (CBS 133991), with strong statistical support (99% MLBS, 1.00 BYPP). Therefore, these two isolates are described as a new species, *D. lignicola*.

**Figure 4.**
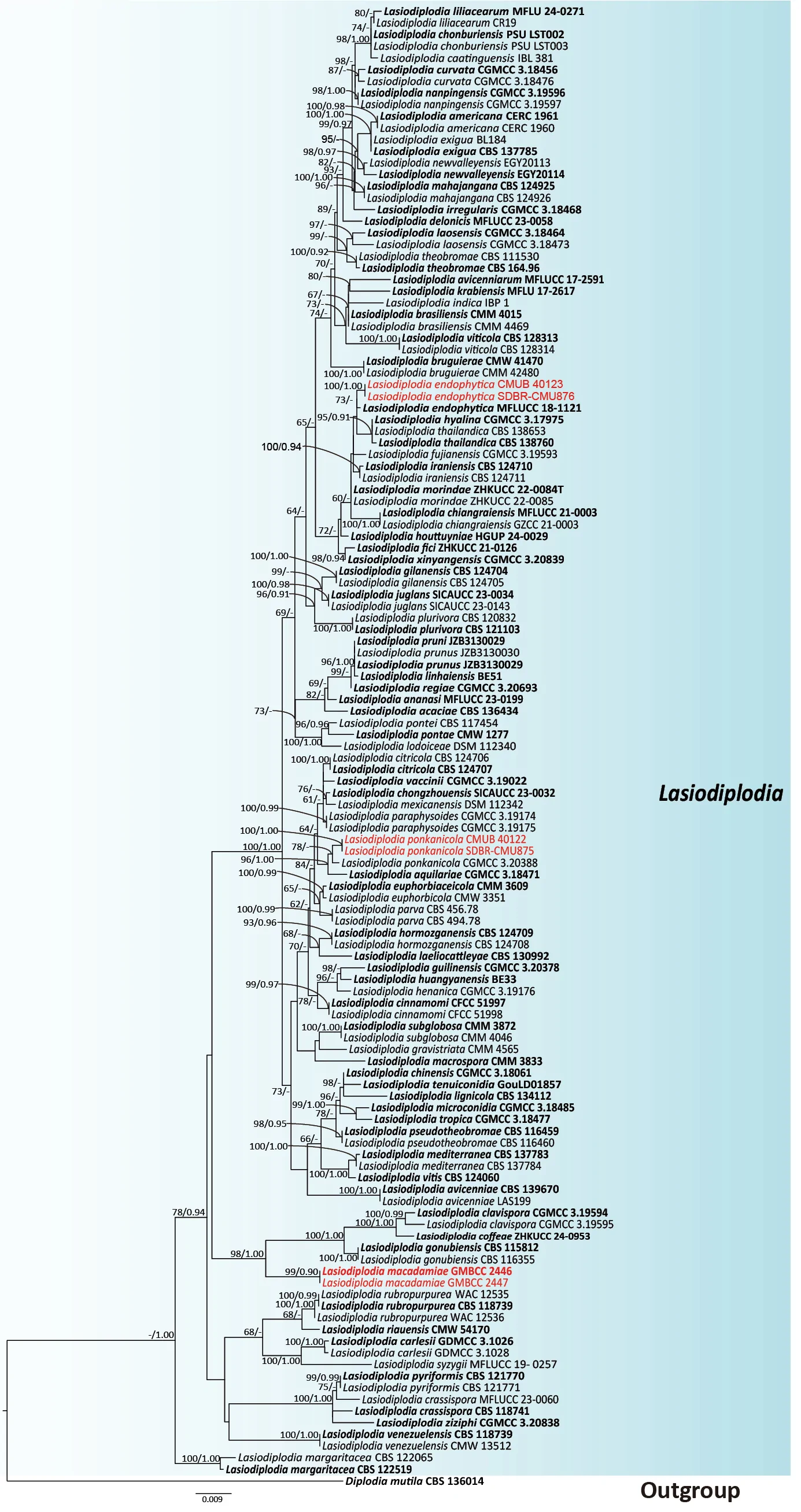
Phylogram generated from maximum likelihood analysis based on a combined dataset of ITS, *tef1-α*, and *tub2* sequence data. The ML tree topology was similar to the BI topology. ML bootstrap support values of 60% or greater and BYPP values of 0.90 or greater are shown above the nodes. All type strains are shown in bold black, and newly generated sequences are shown in red.

Analysis 4: The phylogenetic analysis of the genus *Lasiodiplodia* was conducted using a combined dataset of ITS, *tef1-α*, and *tub2* sequence data following Zhou et al. (2024) and Zhang et al. (2026). A total of 127 strains were included in the combined sequence analysis, which comprised 1,628 characters with gaps. *Diplodia mutila* (CBS 136014) was used as the outgroup taxon. The ML tree topology was similar to the BI topology. Therefore, only the ML tree is presented in Fig. 4. The best-scoring RAxML tree with a final likelihood value of −7361.068753 is presented. The matrix contained 532 distinct alignment patterns, with 27.44% of characters undetermined or missing. Estimated base frequencies were as follows: A = 0.210377, C = 0.306283, G = 0.256092, T = 0.228248; substitution rates AC = 1.000000, AG = 2.987354, AT = 1.000000, CG = 1.000000, CT = 4.262328, GT = 1.000000. A total of six fungal isolates obtained in this study were placed within the genus *Lasiodiplodia*. The two isolates, CMUB 40122 and SDBR-CMU875, clustered with *L. ponkanicola* and formed a well-supported lineage (78% MLBS) and were therefore identified as *L. ponkanicola*. In addition, the two isolates, CMUB 40123 and SDBR-CMU876, clustered with *L. endophytica* strain MFLUCC 18-1121 with 73% MLBS statistical support and were therefore identified as *L. endophytica*. The remaining two isolates, GMBCC 2446 and GMBCC 2447, formed a distinct, well-supported lineage (98% MLBS, 1.00 BYPP) and are proposed as a new species, *L. macadamiae*.

**Figure 5.**
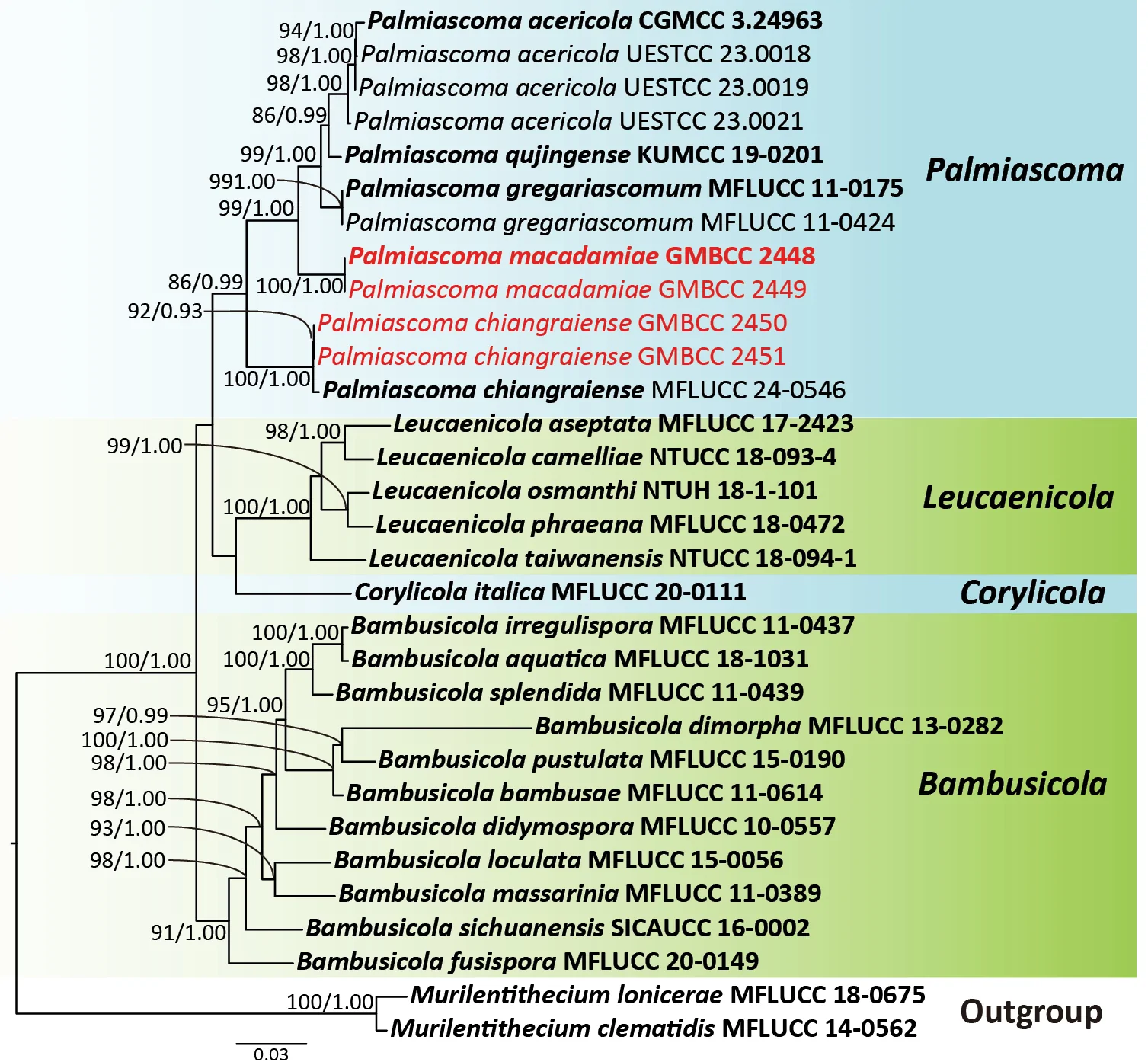
Phylogram generated from maximum likelihood analysis based on a combined dataset of nrLSU, ITS, nrSSU, and *rpb2* sequence data. The ML tree topology was similar to the BI topology. ML bootstrap support values of 60% or greater and BYPP values of 0.90 or greater are shown above the nodes. All type strains are shown in bold black, and newly generated sequences are shown in red.

Analysis 5: The phylogenetic analysis of the species of *Palmiascoma* was conducted based on a combined dataset of nrLSU, ITS, nrSSU, and *rpb2* sequence data following Sun et al. (2025). A total of 30 strains were included in the combined sequence analysis, which comprised 3,298 characters with gaps. *Murilentithecium clematidis* (MFLUCC 14-0562) and *Murilentithecium lonicerae* (MFLUCC 18-0675) were used as the outgroup taxa. The ML tree topology was similar to the BI topology. Therefore, only the ML tree is presented in Fig. 5. The best-scoring RAxML tree with a final likelihood value of −13933.604 is presented. The matrix contained 951 distinct alignment patterns, with 13.73% of characters undetermined or missing. Estimated base frequencies were as follows: A = 0.250562, C = 0.250730, G = 0.250256, T = 0.250453; substitution rates AC = 1.372348, AG = 3.498633, AT = 1.000000, CG = 1.372347, CT = 7.158491, GT = 1.000000; proportion of invariable sites *I* = 0.589309; gamma distribution shape parameter *α* = 0.546238. The phylogenetic tree showed that the *Palmiascoma* clade was strongly supported (86% MLBS, 0.99 BYPP) and was clearly separated from the other genera. The four *Palmiascoma* isolates obtained in this study formed two distinct monophyletic clades. The two isolates GMBCC 2448 and GMBCC 2449 formed a distinct and well-supported lineage (99% MLBS, 1.00 BYPP), representing a new species, *P. macadamiae*. The other two isolates, GMBCC 2450 and GMBCC 2451, formed a well-supported lineage with *P. chiangraiense* (MFLUCC 24-0546) and represent a new host record of *P. chiangraiense*.

**Figure 6.**
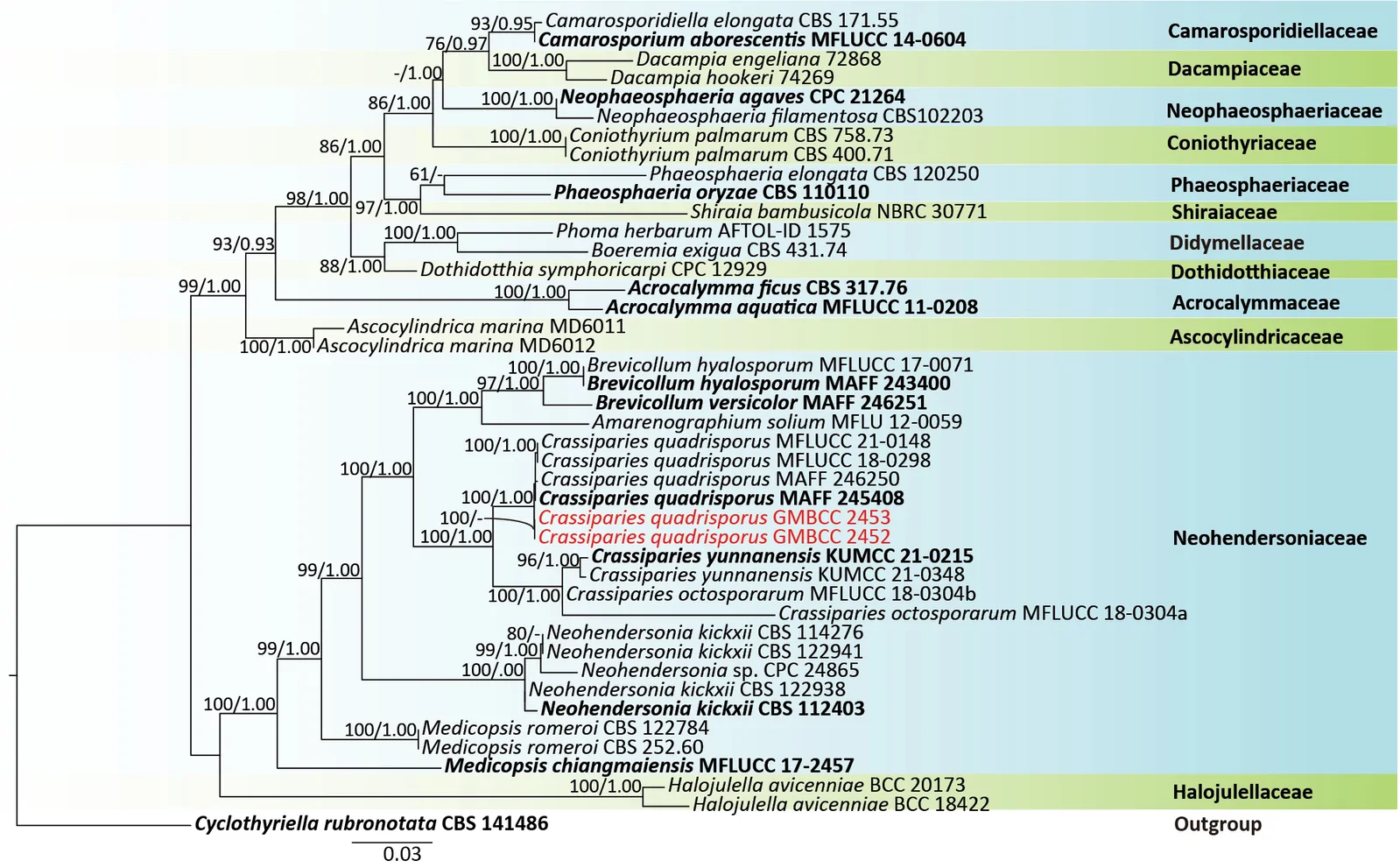
Phylogram generated from maximum likelihood analysis based on a combined dataset of ITS, nrLSU, nrSSU, *tef1-α*, and *rpb2* sequence data. The ML tree topology was similar to the BI topology. ML bootstrap support values of 60% or greater and BYPP values of 0.90 or greater are shown above the nodes. All type strains are shown in bold black, and newly generated sequences are shown in red.

Analysis 6: The phylogenetic analysis of the genus *Crassiparies* was conducted using a combined dataset of ITS, nrLSU, nrSSU, *tef1-α*, and *rpb2* sequences following Lu et al. (2022). A total of 43 strains were included in the combined sequence analysis, which comprised 4,432 characters with gaps. *Cyclothyriella rubronotata* (CBS 141486) was used as the outgroup taxon. The ML tree topology was similar to the BI topology. Therefore, only the ML tree is presented in Fig. 6. The best-scoring RAxML tree with a final likelihood value of −21428.969985 is presented. The matrix contained 1,524 distinct alignment patterns, with 42.07% of characters undetermined or missing. Estimated base frequencies were as follows: A = 0.249122, C = 0.237069, G = 0.268948, T = 0.244860; substitution rates AC = 1.714269, AG = 3.905301, AT = 1.953992, CG = 1.108375, CT = 7.859984, GT = 1.000000; proportion of invariable sites *I* = 0.479072; gamma distribution shape parameter *α* = 0.580554. The phylogenetic tree showed that the two isolates obtained in this study (GMBCC 2452 and GMBCC 2453) were nested within *C. quadrisporus* strains (MAFF 245408, MAFF 245408, MFLUCC 18-0298, and MFLUCC 21-0148) and formed a well-supported lineage (100% MLBS, 1.00 BYPP). Therefore, these two isolates are identified as *C. quadrisporus*.

**Figure 7.**
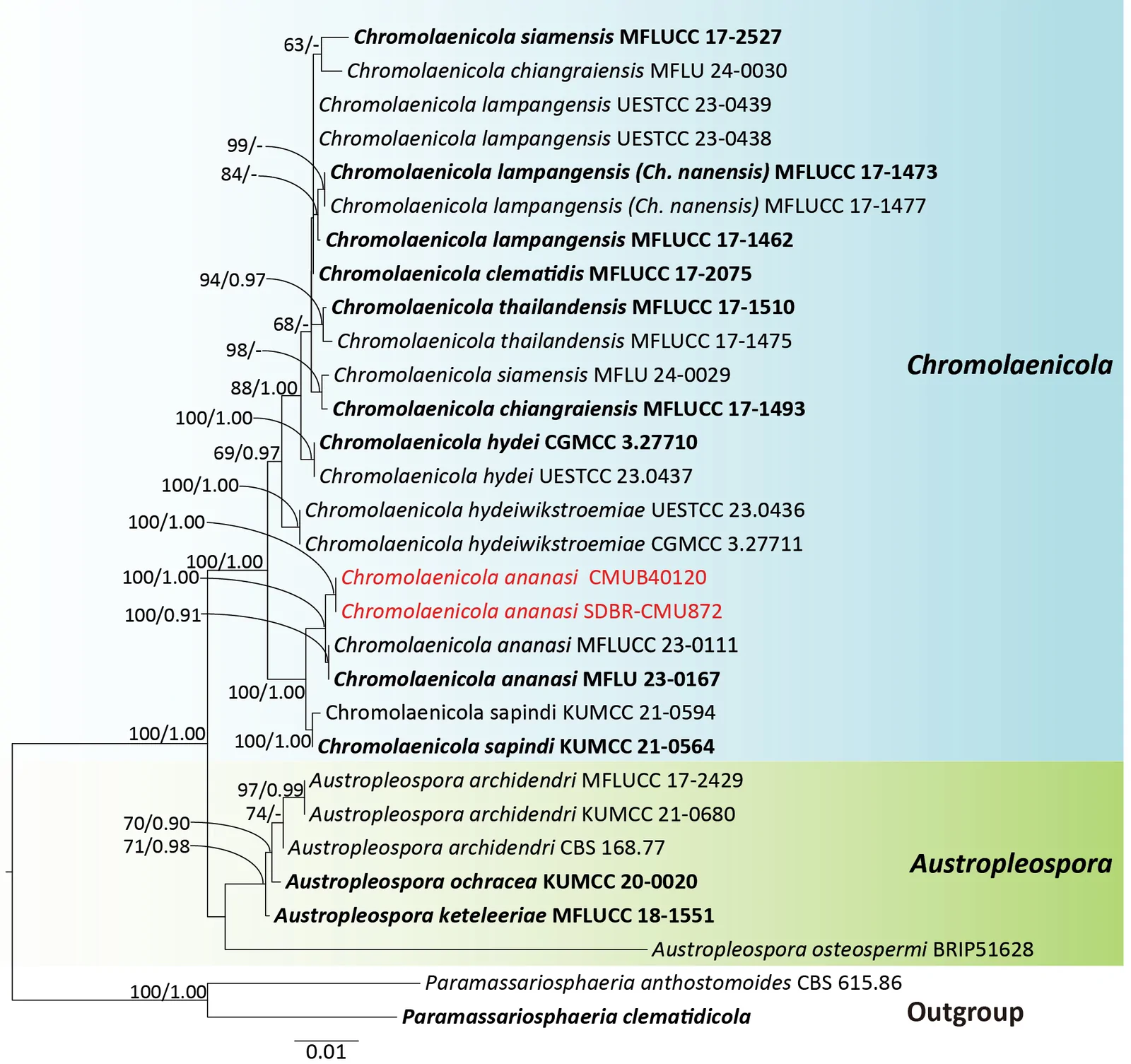
Phylogram generated from maximum likelihood analysis based on a combined dataset of ITS, nrLSU, nrSSU, *tef1-α*, and *rpb2* sequence data. The ML tree topology was similar to the BI topology. ML bootstrap support values of 60% or greater and BYPP values of 0.90 or greater are shown above the nodes. All type strains are shown in bold black, and newly generated sequences are shown in red.

Analysis 7: The phylogenetic analysis of species within the genus *Chromolaenicola* was conducted using a combined dataset of ITS, nrLSU, nrSSU, *tef1-α*, and *rpb2* sequence data following Du et al. (2025a). A total of 30 strains were included in the combined sequence analysis, which comprised 3,351 characters with gaps. *Paramassariosphaeria anthostomoides* (CBS 615.86) and *Paramassariosphaeria clematidicola* (MFLU 16-0172) were used as the outgroup taxa. The ML tree topology was similar to the BI topology. Therefore, only the ML tree is presented in Fig. 7. The best-scoring RAxML tree with a final likelihood value of −7173.931452 is presented. The matrix contained 382 distinct alignment patterns, with 25.28% of characters undetermined or missing. Estimated base frequencies were as follows: A = 0.232658, C = 0.257851, G = 0.270185, T = 0.239307; substitution rates AC = 1.197274, AG = 1.631475, AT = 1.105977, CG = 0.799163, CT = 5.824551, GT = 1.000000; proportion of invariable sites *I* = 0.674366; gamma distribution shape parameter *α* = 0.709915. The multilocus phylogenetic analysis showed that the two isolates obtained in this study (CMUB 40120 and SDBR-CMU872) were nested with *Ch. ananasi* (MFLU 23-0167 and MFLUCC 23-0111) in a well-supported lineage (100% MLBS, 1.00 BYPP). Therefore, they are identified as *Ch. ananasi*.

**Figure 8.**
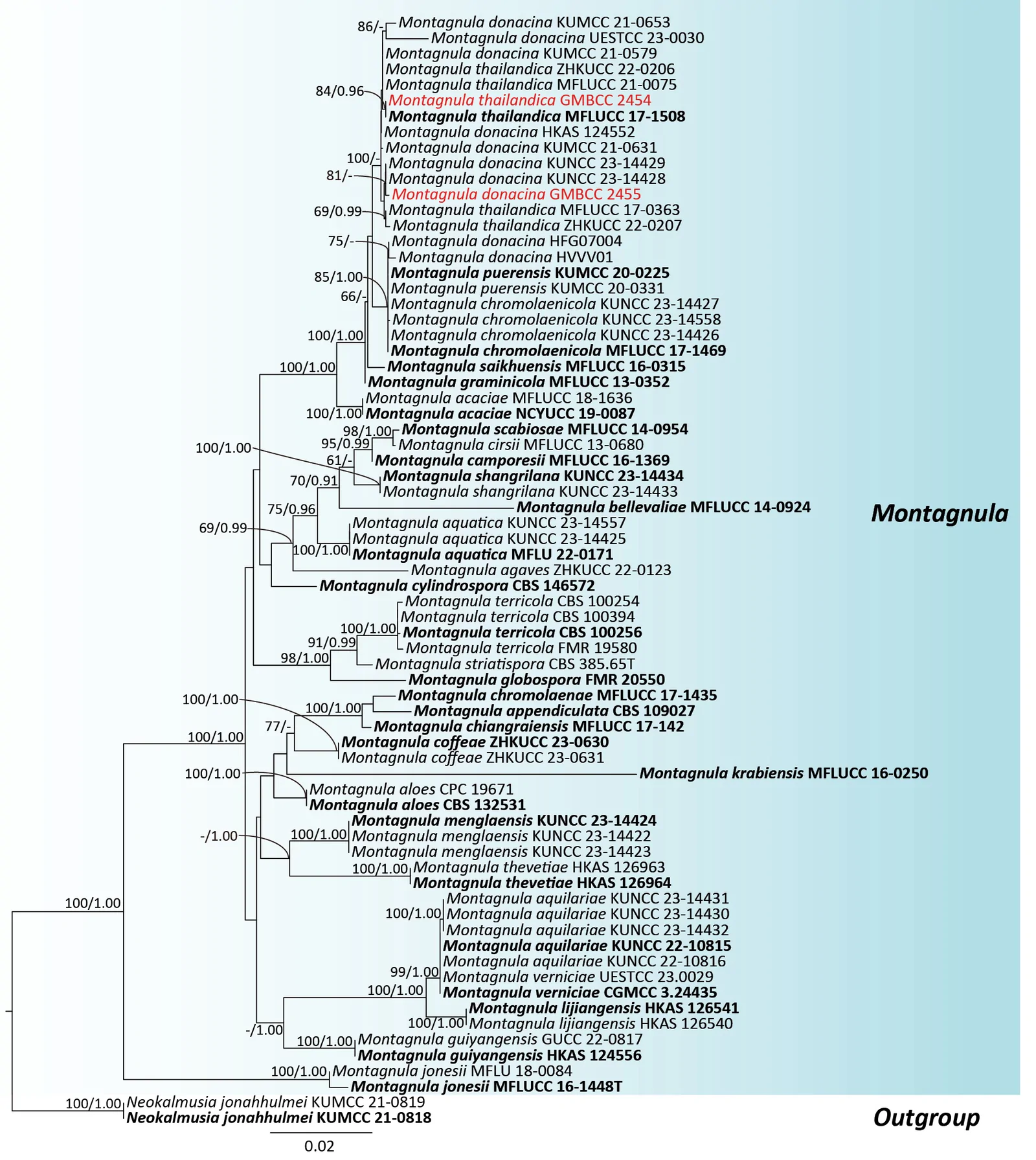
Phylogram generated from maximum likelihood analysis based on a combined dataset of ITS, nrLSU, nrSSU, and *tef1-α* sequence data. The ML tree topology was similar to the BI topology. ML bootstrap support values of 60% or greater and BYPP values of 0.90 or greater are shown above the nodes. All type strains are shown in bold black, and newly generated sequences are shown in red.

Analysis 8: The phylogenetic analysis of *Montagnula* species was conducted using a combined dataset of ITS, nrLSU, nrSSU, and *tef1-α* sequences following Wanasinghe et al. (2024). Seventy-one strains were included in the combined sequence analysis, which comprised 3,324 characters with gaps. *Neokalmusia jonahhulmei* (KUMCC 21-0818T) and *Neokalmusia jonahhulmei* (KUMCC 21-0819) were used as the outgroup taxa. The ML tree topology was similar to the BI topology. Therefore, only the ML tree is presented in Fig. 8. The best-scoring RAxML tree with a final likelihood value of −11807.011024 is presented. The matrix contained 800 distinct alignment patterns, with 16.94% of characters undetermined or missing. Estimated base frequencies were as follows: A = 0.239518, C = 0.258465, G = 0.270946, T = 0.231072; substitution rates AC = 1.857369, AG = 3.055749, AT = 1.470808, CG = 1.117958, CT = 9.363964, GT = 1.000000; proportion of invariable sites *I* = 0.593192; gamma distribution shape parameter *α* = 0.662679. Multilocus phylogenetic analysis showed that one isolate, GMBCC 2454, was nested within *M. thailandica* in a well-supported lineage (84% MLBS, 0.96 BYPP); another isolate, GMBCC 2455, clustered with *M. donacina* with 81% ML bootstrap support. Therefore, they are identified as *M. thailandica* and *M. donacina*.

**Figure 9.**
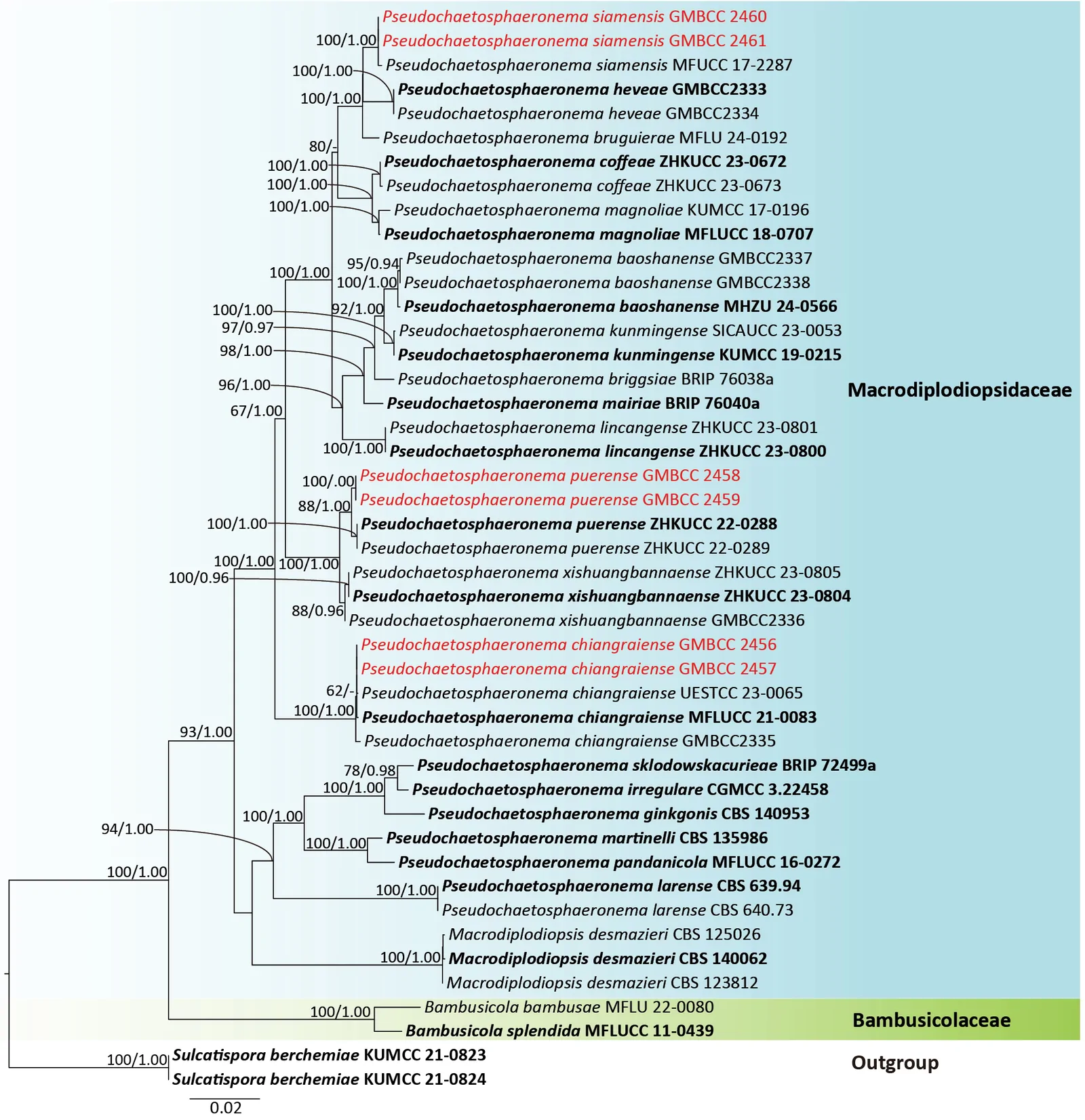
Phylogram generated from maximum likelihood analysis based on a combined dataset of ITS, nrLSU, nrSSU, and *tef1-α* sequence data. The ML tree topology was similar to the BI topology. ML bootstrap support values of 60% or greater and BYPP values of 0.90 or greater are shown above the nodes. All type strains are shown in bold black, and newly generated sequences are shown in red.

Analysis 9: The phylogenetic analysis of *Pseudochaetosphaeronema* species was conducted using a combined dataset of ITS, nrLSU, nrSSU, and *tef1-α* sequences following Apurillo et al. (2025). Forty-five strains were included in the combined sequence analysis, which comprised 3,387 characters with gaps. *Sulcatispora berchemiae* (KUMCC 21-0823) and *Sulcatispora berchemiae* (KUMCC 21-0824) were used as the outgroup taxa. The ML tree topology was similar to the BI topology. Therefore, only the ML tree is presented in Fig. 9. The best-scoring RAxML tree with a final likelihood value of −11641.241405 is presented. The matrix contained 758 distinct alignment patterns, with 12.56% of characters undetermined or missing. Estimated base frequencies were as follows: A = 0.237749, C = 0.251987, G = 0.269533, T = 0.240732; substitution rates AC = 1.091671, AG = 3.413617, AT = 2.115619, CG = 1.229077, CT = 8.686784, GT = 1.000000; proportion of invariable sites *I* = 0.632918; gamma distribution shape parameter *α* = 0.512730. The phylogenetic tree showed that the *Pseudochaetosphaeronema* clade was strongly supported (93% MLBS, 1.00 BYPP). Six *Pseudochaetosphaeronema* isolates from this study fell into three distinct clades corresponding to *Pse. siamense*, *Pse. puerense*, and *Pse. chiangraiense*, each with strong statistical support. Therefore, they are identified as *Pse. siamense* (GMBCC 2460 and GMBCC 2461), *Pse. puerense* (GMBCC 2458 and GMBCC 2459), and *Pse. chiangraiense* (GMBCC 2456 and GMBCC 2457).

**Figure 10.**
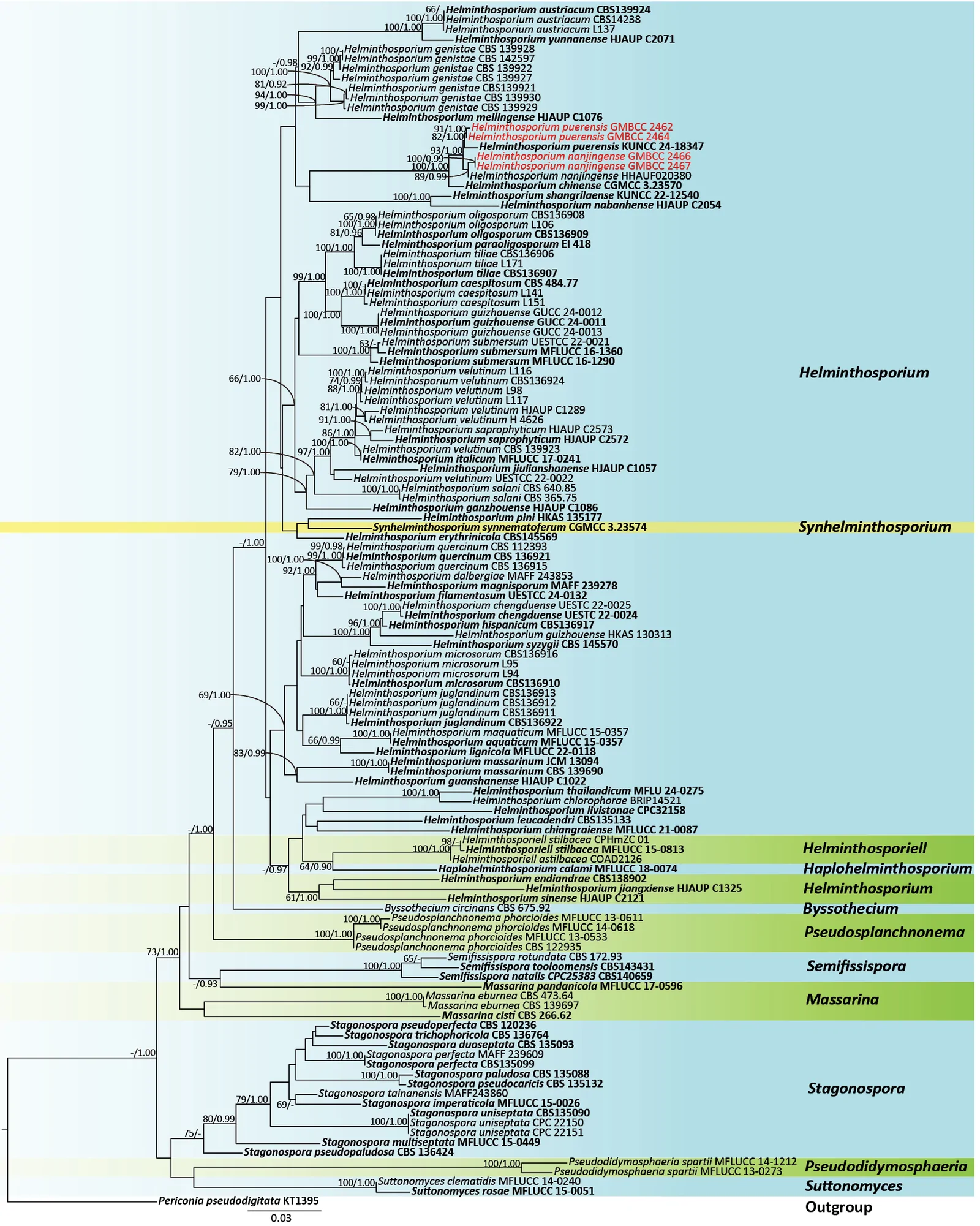
Phylogram generated from maximum likelihood analysis based on a combined dataset of nrSSU, nrLSU, ITS, *rpb2*, and *tef1-α* sequence data. The ML tree topology was similar to the BI topology. ML bootstrap support values of 60% or greater and BYPP values of 0.90 or greater are shown above the nodes. All type strains are shown in bold black, and newly generated sequences are shown in red.

Analysis 10: The phylogenetic analysis of *Helminthosporium* species was conducted using a combined dataset of nrSSU, nrLSU, ITS, *rpb2*, and *tef1-α* sequences following Liao et al. (2025). One hundred and twenty-five strains were included in the combined sequence analysis, which comprised 5,111 characters with gaps. *Periconia pseudodigitata* (CBS 139699) was used as the outgroup taxon. The ML tree topology was similar to the BI topology. Therefore, only the ML tree is presented in Fig. 10. The best-scoring RAxML tree with a final likelihood value of −36400.855198 is presented. The matrix contained 2,133 distinct alignment patterns, with 38.91% of characters undetermined or missing. Estimated base frequencies were as follows: A = 0.239473, C = 0.252773, G = 0.270386, T = 0.237368; substitution rates AC = 1.540445, AG = 4.461885, AT = 1.674575, CG = 0.965087, CT = 7.862411, GT = 1.000000; proportion of invariable sites *I* = 0.491607; gamma distribution shape parameter *α* = 0.496098. The multilocus phylogenetic analysis showed that four isolates obtained in this study belonged to the genus *Helminthosporium*. Phylogenetically, isolates GMBCC 2462 and GMBCC 2464 clustered with *H. puerense* (KUNCC 24-18347) and are identified herein as *H. puerense*. Isolates GMBCC 2466 and GMBCC 2467 clustered with *H. nanjingense* (HHAUF020380) in a highly supported lineage (89% MLBS, 0.99 BYPP) and are identified herein as *H. nanjingense*.

### Taxonomy

#### Xylochrysis macadamiae

Taxon classificationFungiSordariomycetesWoswasiaceae

X. Zhang, Tibpromma & Karun. sp. nov.

0B8AEBC2-AEA6-5F46-9E73-16A5E1219FA8

863022

Figs 1, 11

##### Etymology.

“*macadamiae*” refers to the host plant genus “*Macadamia*.”

**Figure 11.**
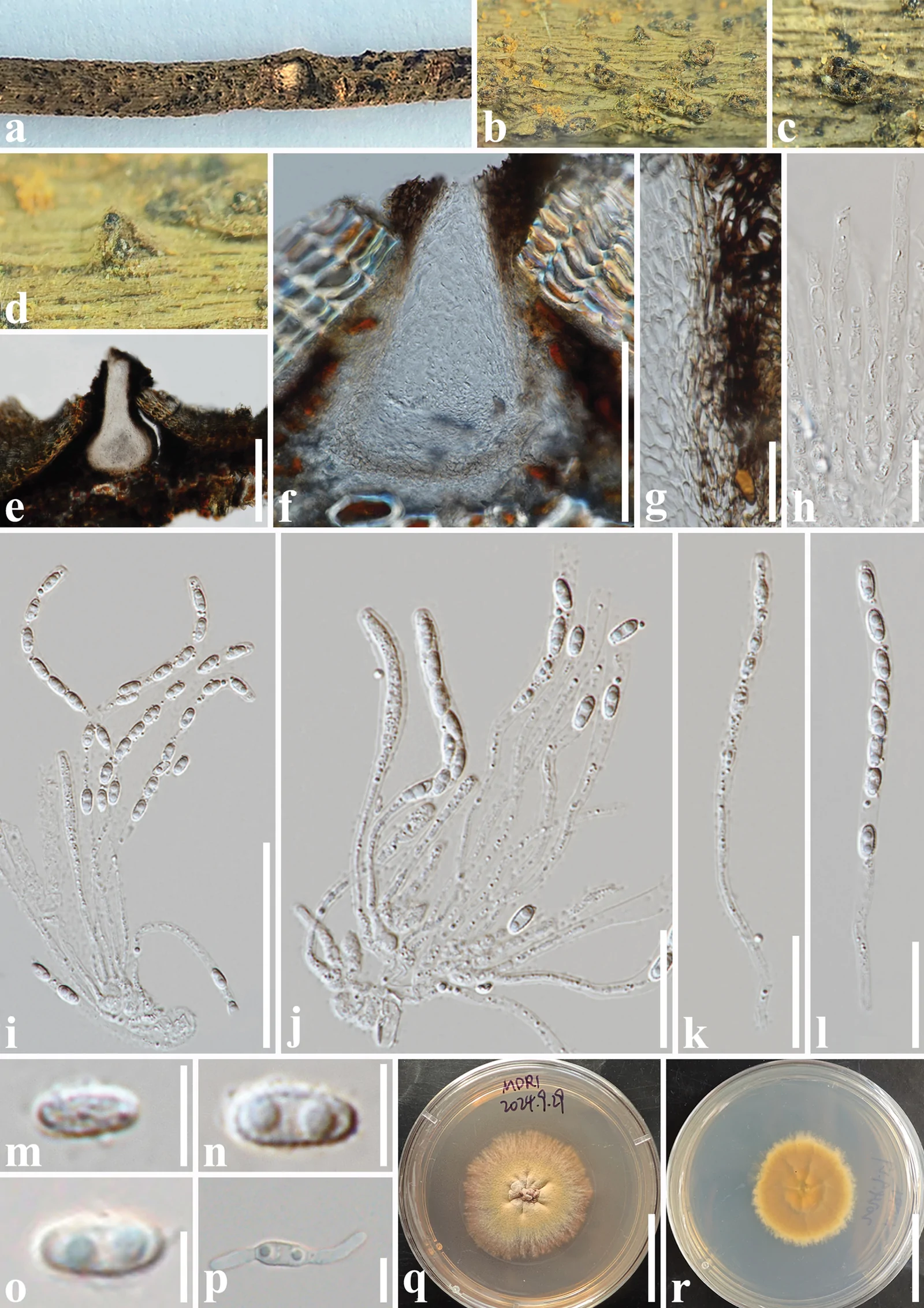
*Xylochrysis macadamiae* (GMB-W 1561, holotype). **a–d**. Appearance of ascomata on substrate; **e, f**. Vertical sections of ascomata; **g**. Peridium; **h**. Hamathecium; **i–l**. Asci; **m–o**. Ascospores; **p**. Germinated ascospore; **q, r**. Culture on PDA from above (**q**) and below (**r**). Scale bars: 200 μm (**e**); 100 μm (**f**); 50 μm (**i**); 20 μm (**g, h, j–l**); 10 μm (**p**); 5 μm (**m–o**); 2 cm (**q, r**).

##### Diagnosis.

*Xylochrysis macadamiae* differs from *X. chiangraiensis* and *X. hydei* by its rough-walled ascospores, distinct nrLSU, nrSSU, ITS, *tef1-α*, and *rpb2* sequences, and distinct phylogenetic position.

##### Holotype.

GMB-W 1561.

##### Description.

***Saprobic*** on dead twigs of *Macadamia* sp. **Sexual morph: *Ascomata*** (including necks) 435–695 × 235–360 µm (x̄ = 528 × 307 µm, *n* = 10), scattered or aggregated or confluent into small groups, semi-immersed, globose to subglobose to pyriform, uniloculate, nonstromatic, glabrous at neck, not surrounded by a pigmented layer of cells. ***Ostioles*** 95–165 × 25–70 µm (x̄ = 133 × 50 µm, *n* = 10), black, glabrous, papillate, shiny, filled with hyaline periphyses. ***Peridium*** multi-layered, with outer layers 20–70 µm thick, carbonized, composed of brown to dark brown cells of textura prismatica, with inner layers 12–34 µm, thin, composed of flattened, hyaline cells of textura prismatica. ***Hamathecium*** 3.5–6.0 µm wide (x̄ = 4.6 μm, *n* = 40), composed of abundant, narrowly cylindrical to filiform, hyaline, branched, septate, guttulate, paraphyses slightly constricted at septa, not embedded in a gelatinous matrix. ***Asci*** (65–) 80–135 (–215) × 3.5–4.5 µm (x̄ = 106 × 4 µm, *n* = 40), 8-spored, unitunicate, cylindrical, long pedicellate, clear apical apparatus, tapering to the bulbous base, with a bulbous long pedicel, apically rounded. ***Ascospores*** 4.5–6.5 × 2–3.5 µm (x̄ = 5.6 × 2.6 µm, *n* = 40), uniseriate, hyaline, oval to ellipsoid, straight, aseptate, 2-guttulate when mature, rough-walled, without mucilaginous sheath or appendages. **Asexual morph**: Not observed.

##### Culture characteristics.

Ascospores germinating on PDA within 24 h at 28 °C, with colonies on PDA reaching 3 cm in diam. after 3 weeks at 28 °C, with a rough surface, filamentous, hairy, slightly raised in the middle, cottony, yellow-brown at the edge, becoming pale yellow toward the center; on the reverse side, as above, light yellow at the margins, turning light brown toward the center, wrinkled, and rarely longitudinally wrinkled in the first circle.

##### Material examined.

China • Yunnan Province, Dehong City, on dead twigs of *Macadamia* sp., 16 April 2024, Xian Zhang, MDR1 (GMB-W 1561), holotype; ex-type living culture, GMBCC 2440, other living culture GMBCC 2441.

##### Notes.

In the phylogenetic analyses, the isolates of *Xylochrysis macadamiae* clustered within *Xylochrysis*, sister to *X. hydei* (GMB-W 1199, holotype, and GMB-W 1200) and *X. chiangraiensis* (MFLUCC 24-0550, ex-type), with 100% MLBS and 1.00 BYPP statistical support (Fig. 1). Morphologically, *X. macadamiae* resembles *X. chiangraiensis* (MFLU 24-0470, holotype) and *X. hydei* (GMB-W 1199, holotype) in having 8-spored, unitunicate, long-pedicellate asci tapering to a bulbous base and hyaline ascospores. However, *X. macadamiae* differs from *X. chiangraiensis* by having larger (435–695 × 235–360 µm vs. 138–282 × 130–216 μm), semi-immersed ascomata, a peridium composed of textura prismatica cells, and rough-walled ascospores, whereas *X. chiangraiensis* has smaller, immersed ascomata, a peridium composed of textura angularis cells, and smooth- and thin-walled ascospores (Afshari et al. 2025b). *Xylochrysis macadamiae* differs from *X. hydei* by having globose to subglobose to pyriform ascomata with long ostioles and oval to ellipsoid, rough-walled ascospores, whereas *X. hydei* has apically rounded asci with an apical ring and ellipsoidal ascospores with thin and smooth walls (Hongsanan et al. 2025). Based on nucleotide comparisons, *X. macadamiae* differs from *X. hydei* (GMB-W 1199) by 12/824 bp (1.5%, without gaps) in nrLSU, 38/545 bp (7.0%, without gaps) in ITS, 110/933 bp (11.8%, without gaps) in *tef1-α*, and 113/1112 bp (10.2%, without gaps) in *rpb2*, and differs from *X. chiangraiensis* (MFLUCC 24-0550) by 15/825 bp (1.82%, without gaps) in nrLSU, 48/526 bp (9.13%, without gaps) in ITS, and 139/1112 bp (12.5%, without gaps) in *rpb2*. Therefore, *Xylochrysis macadamiae* is introduced here as a new species in *Xylochrysis*, collected from *Macadamia* sp. in China.

#### Xylochrysis yunnanensis

Taxon classificationFungiSordariomycetesWoswasiaceae

X. Zhang, Tibpromma & Karun. sp. nov.

94E13853-5A17-5D35-9ECF-B195E4494EA6

863023

Figs 1, 12

##### Etymology.

The species epithet refers to the type locality, Yunnan, China.

**Figure 12.**
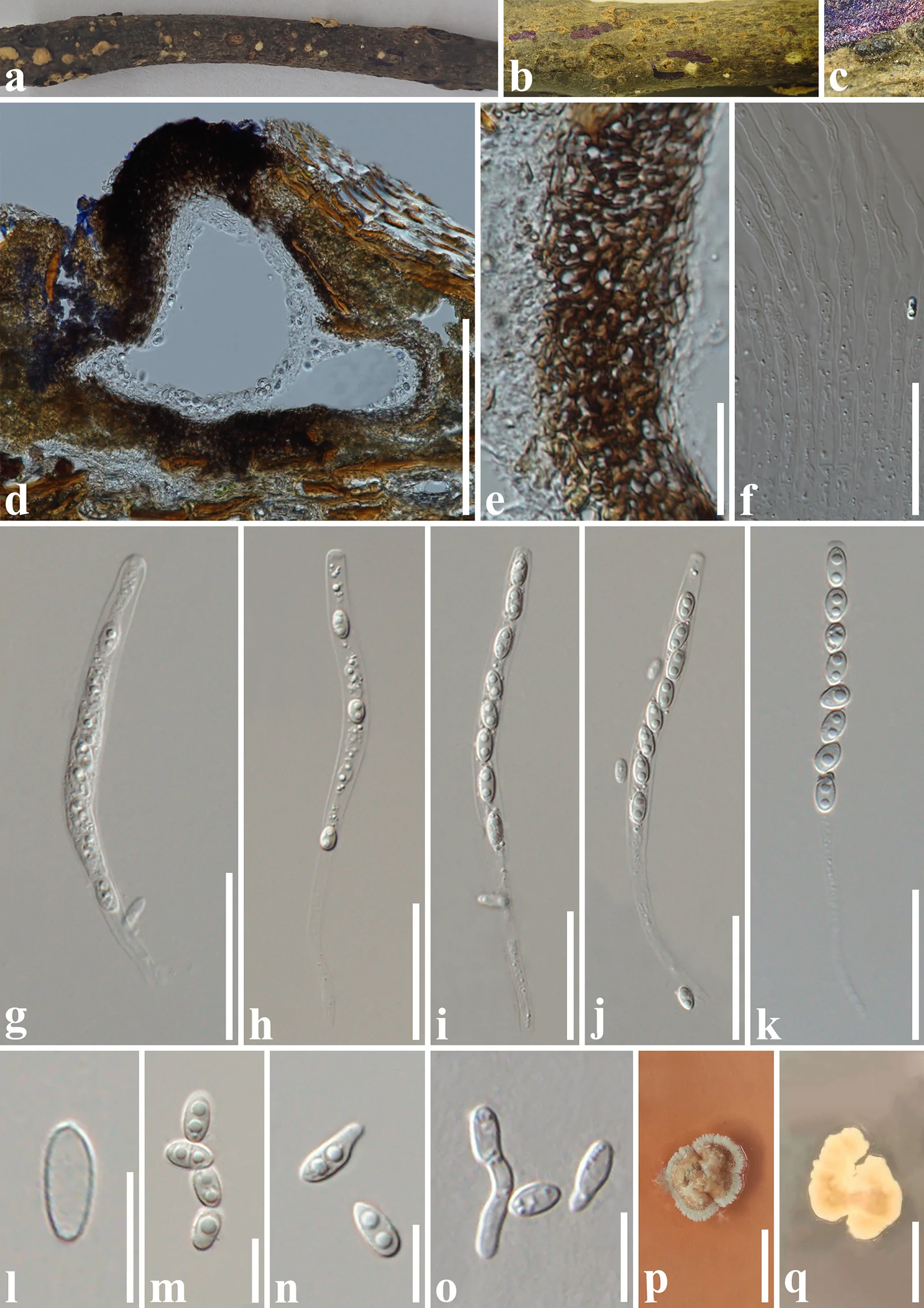
*Xylochrysis yunnanensis* (GMB-W 1562, holotype). **a–c**. Appearance of ascomata on substrate; **d**. Vertical section of ascoma; **e**. Peridium; **f**. Hamathecium; **g–k**. Asci; **l–n**. Ascospores; **o**. Germinated ascospores; **p, q**. Culture on PDA from above (**p**) and below (**q**). Scale bars: 100 μm (**d**); 30 μm (**f–k**); 10 μm (**l–o**); 1 cm (**p, q**).

##### Diagnosis.

*Xylochrysis yunnanensis* differs from *X. aquatica*, *X. guttulata*, and *X. lucida* by its oval to ellipsoid ascospores, distinct nrLSU, nrSSU, ITS, *tef1-α*, and *rpb2* sequences, and distinct phylogenetic position.

##### Holotype.

GMB-W 1562.

##### Description.

***Saprobic*** on dead twigs of *Macadamia* sp. **Sexual morph: *Ascomata*** 150–225 × 150–320 µm (x̄ = 193 × 238 µm, *n* = 10), scattered, semi-immersed, subglobose to irregular, glabrous, uniloculate, nonstromatic, short ostiolate. ***Peridium*** 15–55 µm (x̄ = 31 × 20 µm, *n* = 10), multi-layered, with outer layers thick, carbonized, composed of brown to dark brown cells of textura angularis, with inner layers thin, composed of flattened, hyaline cells of textura prismatica. ***Hamathecium*** 2.5–6.0 µm wide (x̄ = 4.2 μm, *n* = 30), composed of abundant, narrowly cylindrical to filiform, hyaline, unbranched, septate with slightly constricted, guttulate, paraphyses not embedded in a gelatinous matrix. ***Asci*** 90–140 × 5.5–7.5 µm (x̄ = 113 × 6.3 µm, *n* = 30), 8-spored, unitunicate, cylindrical, long pedicellate, tapering to a bulbous base, apically rounded, with a clear apical apparatus. ***Ascospores*** 6–8.5 (–11) × 3.5–5.0 µm (x̄ = 7.3 × 4 µm, *n* = 45), uniseriate, hyaline, oval to ellipsoid, aseptate or 1- septate, 1–2-guttulate, straight, without mucilaginous sheath or appendages. **Asexual morph**: Not observed.

##### Culture characteristics.

Ascospores germinating on PDA within 24 h at 28 °C, producing colonies on PDA reaching 1.5 cm in diam. after 3 weeks at 28 °C, with occasional orange pigmentation diffusing into the medium. Colonies with a rough surface, filamentous, hairy, irregular, raised, cottony, and white-yellow from above; light yellow to light brown and irregular in reverse.

##### Materials examined.

China • Yunnan Province, Dehong City, on dead twigs of *Macadamia* sp., 24 April 2024, Xian Zhang, MYD5 (GMB-W 1562), holotype; ex-type living culture, GMBCC 2442, other living culture GMBCC 2443.

##### Notes.

In the phylogenetic analyses, the isolates of *Xylochrysis yunnanensis* were placed as an independent clade within *Xylochrysis*, with 100% MLBS and 1.00 BYPP statistical support (Fig. 1). The collection shares similar morphology with *X. guttulata*, *X. lucida*, and *X. aquatica* in having semi-immersed ascomata, 8-spored, cylindrical asci, and hyaline, 2-guttulate ascospores (Réblová et al. 2014; Song et al. 2023; Xiao and Liu 2025). However, *X. yunnanensis* can be distinguished from *X. guttulata* (HKAS 135169, holotype) by having unbranched, septate paraphyses slightly constricted at the septa, wider (90–140 × 5.5–7.5 µm vs. 70–110 × 3.5–4.5 μm) asci, and aseptate or 1-septate, oval to ellipsoid ascospores, whereas *X. guttulata* has a hamathecium with branched paraphyses and lanceolate to oval ascospores (Xiao and Liu 2025). It differs from *X. lucida* (PRM 922621, holotype) by having wider paraphyses (2.5–6.0 µm vs. 2.5–3.0 µm), bulbous, long-pedicellate asci, and oval to ellipsoid, aseptate or 1-septate ascospores, whereas *X. lucida* has ellipsoidal and unicellular ascospores (Réblová et al. 2014). It also differs from *X. aquatica* (HKAS 124019, holotype) by having larger (6–8.5(–11) × 3.5–5.0 μm vs. 4.5–6.5 × 2.5–4.0 μm), oval to ellipsoid, aseptate or 1-septate ascospores and slightly constricted paraphyses, whereas *X. aquatica* has ellipsoidal, aseptate, 2-guttulate ascospores (Song et al. 2023). Based on both morphological characteristics and phylogenetic evidence, *Xylochrysis yunnanensis* is introduced as a novel species.

#### Sporidesmium tropicale

Taxon classificationFungiPleosporales

M.B. Elli, Mycology Pap. 70: 58 (1958)

444F299C-B58D-5A6D-A729-C6D22CEF3990

306326

Fig. 2

##### Description.

For the original description, see Bao et al. (2021).

##### Known hosts.

*Hevea brasiliensis* (Senwanna et al. 2021), *Macadamia* sp. (this study), *Pinus yunnanensis* (Zhang et al. 2025a), *Psidium guajava* (Ellis 1971), unknown submerged decaying wood (Yang et al. 2018; Bao et al. 2021), and *Camellia sinensis* (Johnston 1960).

##### Known distribution.

China (Yunnan Province; Bao et al. 2021; Zhang et al. 2025a; this study), Sierra Leone (Ellis 1971), Thailand (Yang et al. 2018; Senwanna et al. 2021), and Malaysia (Johnston 1960).

##### Materials examined.

China • Yunnan Province, Pu’er City, on dead twigs of *Macadamia* sp., 1 August 2024, Xian Zhang, MYP22 (GMB-W 1563); living culture, GMBCC 2444, other living culture GMBCC 2445.

##### Notes.

In the phylogenetic analyses, the isolates (GMBCC 2444 and GMBCC 2445) were nested with *Sporidesmium tropicale* strains (GMB 5105, GMB 5109, DLUCC 1689, MFLU 17-0850, and MFLUCC 17-0344) with 89% ML bootstrap support (Fig. 2). Ellis (1971) introduced *Sporidesmium tropicale* from decaying wood in Sierra Leone based on its asexual morph. The morphology of the collection is similar to that of *S. tropicale* (MFLUCC 17-0344), with terminal and intercalary conidiophores and verruculose, obclavate, rostrate conidia that are truncate at the base. Later, *Sporidesmium tropicale* was found on *Hevea brasiliensis*, *Pinus yunnanensis*, and unknown wood in China and Thailand (Yang et al. 2018; Bao et al. 2021; Senwanna et al. 2021; Zhang et al. 2025a). In this study, *S. tropicale* is introduced as a new host record collected from *Macadamia* sp. in China.

#### Dothiorella lignicola

Taxon classificationFungiBotryosphaerialesBotryosphaeriaceae

X. Zhang & N. Suwannar. sp. nov.

52BF62D0-53B9-5142-BB1B-2D1CB9BABC64

863024

Figs 3, 13

##### Etymology.

The name refers to the lignicolous habit of the species.

**Figure 13.**
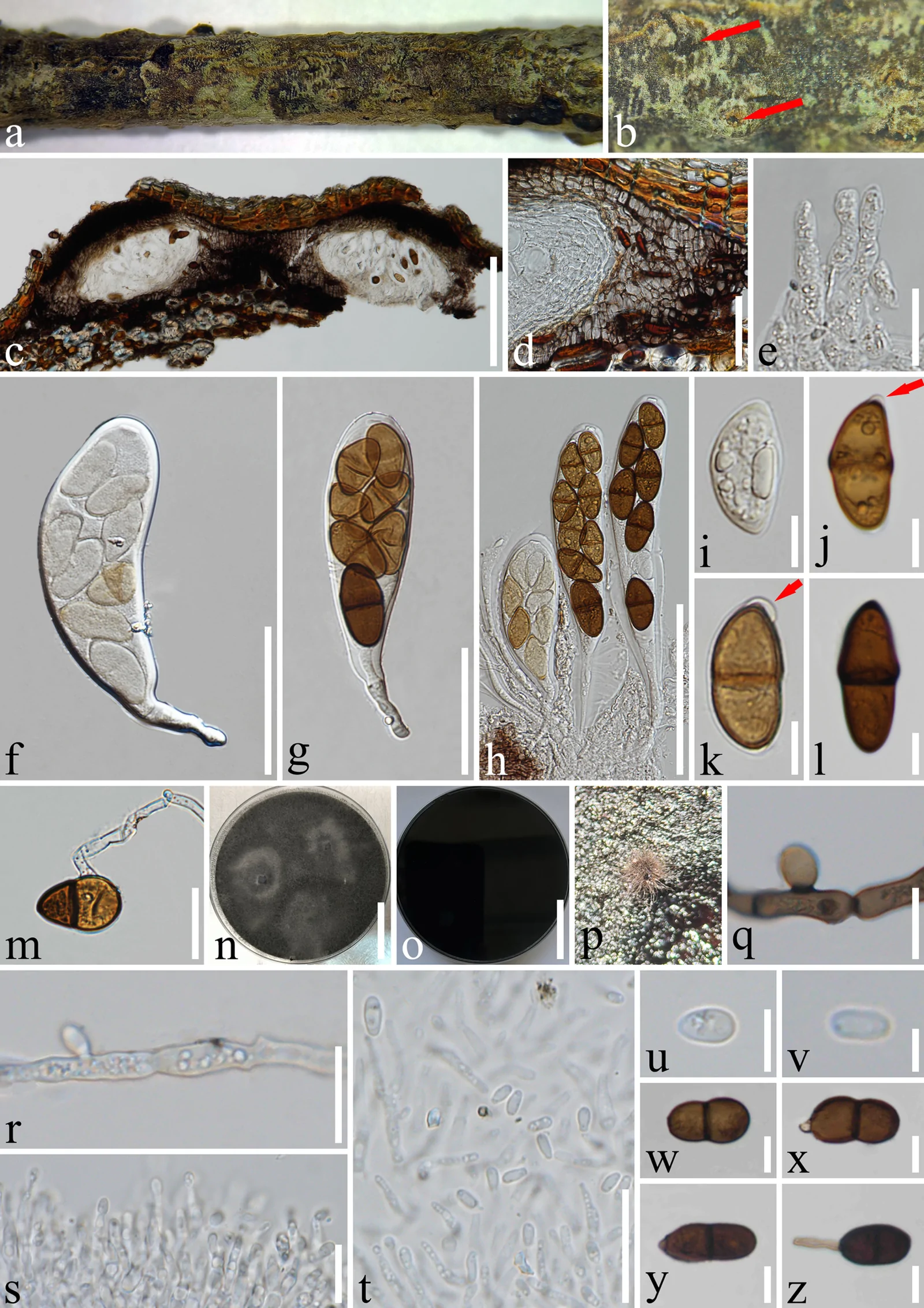
*Dothiorella lignicola* (CMUB 40121, holotype). **a, b**. Appearance of ascomata on substrate; **c**. Vertical section of ascoma; **d**. Peridium; **e**. Hamathecium; **f–h**. Asci; **i–l**. Ascospores (arrows indicate mucilaginous polar appendages); **m**. Germinated ascospore; **n, o**. Culture on PDA from above (**n**) and below (**o**); **p**. Sporulating colonies on PDA; **q, r**. Chlamydospores; **s, t**. Conidiogenous cells with conidia; **u–z**. Conidia. Scale bars: 200 μm (**c**); 100 μm (**h**); 50 μm (**d, f, g**); 30 μm (**m, t**); 20 μm (**e, q–s**); 10 μm (**i–l, u, w–z**); 5 μm (**v**); 3 cm (**n, o**).

##### Diagnosis.

*Dothiorella lignicola* differs from *D. albiziae*, *D. dulcispinae*, and *D. thailandica* by its ovoid conidia; from *D. oblonga* by its aseptate conidia; and by distinct ITS, *tub2*, and *tef1-α* sequences and a distinct phylogenetic position.

##### Holotype.

CMUB 40121.

##### Description.

***Saprobic*** on dead twigs of *Macadamia* sp. **Sexual morph: *Ascomata*** 80–120 × 160–220 µm (x̄ = 100 × 193 µm, *n* = 10), immersed, visible as dark dots on host surface, dark brown to black, ampulliform, papillate, without ostiole. ***Peridium*** 50–190 µm wide (x̄ = 95 µm, *n* = 30), composed of three layers, outer layer dark brown to black cells of textura prismatica, middle layer light brown to brown cells of texture angularis, and inner hyaline cells of texture angularis. ***Hamathecium*** composed of 4.5–8.5 µm wide (x̄ = 6.5 μm, *n* = 30), hyaline, septate, pseudoparaphyses slightly constricted at septum. ***Asci*** 110–235 × 23–38 µm (x̄ = 173 × 32 µm, *n* = 30), 8-spored, bitunicate, clavate to cylindric-clavate, pedicel, straight or slightly curved, with well-developed ocular chamber, rounded at apex, irregularly biseriate. ***Ascospores*** (27–)30–37(–40) × 14–19 µm (x̄ = 33 × 17 µm, *n* = 60), irregularly overlapping, subglobose to elliptical, 0–1-septate, slightly or constricted at septum, subhyaline to pale brown, brown to dark brown on maturity with a dark brown band at septa, smooth-walled, cells obtuse towards ends, cells often slightly thickened at midpoint, with or without mucilaginous polar appendages at one or both ends. **Asexual morph: *Conidiomata*** produced in culture on PDA after seven weeks at room temperature, solitary, superficial, brown to dark brown, hairy, subglobose to globose, covered with hyphal hairs, unilocular. ***Conidiophores*** reduced to conidiogenous cells. ***Conidiogenous cells*** holoblastic, discrete, cylindrical to subcylindrical, sometimes slightly tapering towards apex, hyaline, smooth, terminal. ***Conidia*** 19–26.5 × 10–13.5 µm (x̄ = 22.6 × 11.2 µm, *n* = 50), ellipsoid to oblong to ovoid, with rounded ends, initially hyaline and aseptate, becoming brown to dark brown and 1-septate when mature, slightly constricted or not constricted at septum, smooth-walled.

##### Culture characteristics.

Ascospores germinating on PDA within 24 h at 28 °C, with colonies on PDA reaching 9 cm in diam. after 1 week at 28 °C, with a rough surface, filamentous, hairy, cottony, dark gray to black, with indistinct concentric zonation from above and dull gray to black from below.

##### Materials examined.

Thailand • Chiang Mai Province, Doi Saket District, 18°52'43"N, 99°13'15"E, 384 m elevation, on a dead twig of *Macadamia* sp., 24 November 2023, Xian Zhang, TCMM51 (CMUB40121), holotype; ex-type living culture, SDBR-CMU873, other living culture SDBR-CMU874.

##### Notes.

In the phylogenetic analyses, the isolates of *Dothiorella lignicola* clustered within *Dothiorella*, sister to *D. albiziae* (MFLUCC 22-0057), *D. asteracearum* (MFLUCC 25-0275), *D. dulcispinae* (CBS 121764 and CBS 130413), *D. oblonga* (CBS 121765 and CBS 121766), and *D. thailandica* (CBS 133991), with 99% MLBS and 1.00 BYPP statistical support (Fig. 3). Morphologically, *D. lignicola* differs from *D. albiziae* (MFLU 22-0093, holotype) by having larger (19–26.5 × 10–13.5 µm vs. 14–18 × 6–8 μm), ellipsoid to oblong to ovoid conidia, whereas *D. albiziae* has solitary or gregarious, immersed conidiomata (Rathnayaka et al. 2022). The isolates (CMUB 40121, holotype) differ from *D. dulcispinae* (PREM 60706, holotype) by having terminal conidiogenous cells and longer and narrower (19–26.5 × 10–13.5 µm vs. (6–)7–10(–11) × (14–)16–22(–24) µm), ellipsoid to oblong to ovoid conidia, whereas *D. dulcispinae* has percurrently proliferating conidiogenous cells near the apex and ovoid conidia (Jami et al. 2012). *Dothiorella lignicola* differs from *D. oblonga* (PREM 59628, holotype) by having initially hyaline and aseptate conidia, whereas *D. oblonga* has honey-colored to brown conidia that become blackish brown at maturity (Slippers et al. 2014). *Dothiorella lignicola* differs from *D. thailandica* (MFLU 12-0751, holotype) by having larger (19–26.5 × 10–13.5 µm vs. 15–20 × 6.5–8 μm), granular, oblong to ovoid conidia that are slightly constricted or not constricted at the septum, whereas *D. thailandica* has oblong to ellipsoidal conidia with a smaller lower cell (Liu et al. 2012; Phillips et al. 2013). *Dothiorella albiziae*, *D. dulcispinae*, *D. oblonga*, and *D. thailandica* have been recorded in their asexual morphs; therefore, the sexual morphological characteristics of *D. lignicola* could not be compared with those of the four previously described species. Regarding the sexual morphological characteristics, the isolates (CMUB 40121, holotype) differ from *D. asteracearum* (MFLU 25-0274, holotype) by having immersed ascomata, a peridium comprising three layers of cells of textura angularis and textura prismatica, and irregularly overlapping ascospores with the apex often slightly thickened, whereas *D. asteracearum* has immersed to semi-immersed ascomata and ascospores without a gelatinous sheath (Htet et al. 2025). *Dothiorella asteracearum* has only been recorded from its sexual morph; therefore, the asexual morphological characteristics could not be compared. Based on nucleotide comparisons, the species (SDBR-CMU873) was compared for ITS, *tef1-α*, and *tub2* with seven strains (Table 4), and the *tef1-α* gene showed more than 20 bp differences. Therefore, *Dothiorella lignicola* is introduced here as a new species in *Dothiorella*, collected from Thailand.

**Table 4.** Nucleotide comparisons of *D. lignicola* (SDBR-CMU873, ex-type) with *D. albiziae* (MFLUCC 22-0057, ex-type), *D. asteracearum* (MFLUCC 25-0275), *D. dulcispinae* (CBS 121764 and CBS 130413, ex-type), *D. oblonga* (CBS 121765, ex-type, and CBS 121766), and *D. thailandica* (CBS 133991), based on ITS, *tef1-α*, and *tub2*. All comparisons excluded gaps; “-” indicates unavailable data.

**Taxa**	**ITS**	***tef1-α***	***tub2***
*D. albiziae* (MFLUCC 22-0057)	3/471 bp (0.6%)	21/241 bp (8.7%)	1/341 bp (0.3%)
*D. asteracearum* (MFLUCC 25-0275)	12/502 bp (2.4%)	–	–
*D. dulcispinae* (CBS 121764)	3/500 bp (0.6%)	25/260 bp (9.6%)	5/431 bp (1.2%)
*D. dulcispinae* (CBS 130413)	3/478 bp (0.6%)	26/263 bp (9.9%)	5/428 bp (1.2%)
*D. oblonga* (CBS 121765)	2/500 bp (0.4%)	26/257 bp (10.1%)	5/431 bp (1.2%)
*D. oblonga* (CBS 121766)	2/500 bp (0.4%)	26/257 bp (10.1%)	5/431 bp (1.2%)
*D. thailandica* (CBS 133991)	5/531 bp (0.9%)	31/298 bp (10.4%)	4/380 bp (1.1%)

*Dothiorella lignicola* was introduced as *D. macadamiae* X. Zhang & N. Suwannar. in Zhang et al. (2025b). However, the name *D. macadamiae* is rejected under Article 52.1 of the International Code of Nomenclature for algae, fungi, and plants (ICN) because the description was based on an incorrect host, species name, etymology, and DNA sequences. Therefore, *D. lignicola* is proposed as a replacement name for *D. macadamiae*.

#### Lasiodiplodia macadamiae

Taxon classificationFungiBotryosphaerialesBotryosphaeriaceae

X. Zhang, Tibpromma & Karun. sp. nov.

B7228FE3-20C0-52F2-A898-DE65D1D6DD41

863025

Figs 4, 14

##### Etymology.

“*macadamiae*” refers to the host plant genus *Macadamia*.

**Figure 14.**
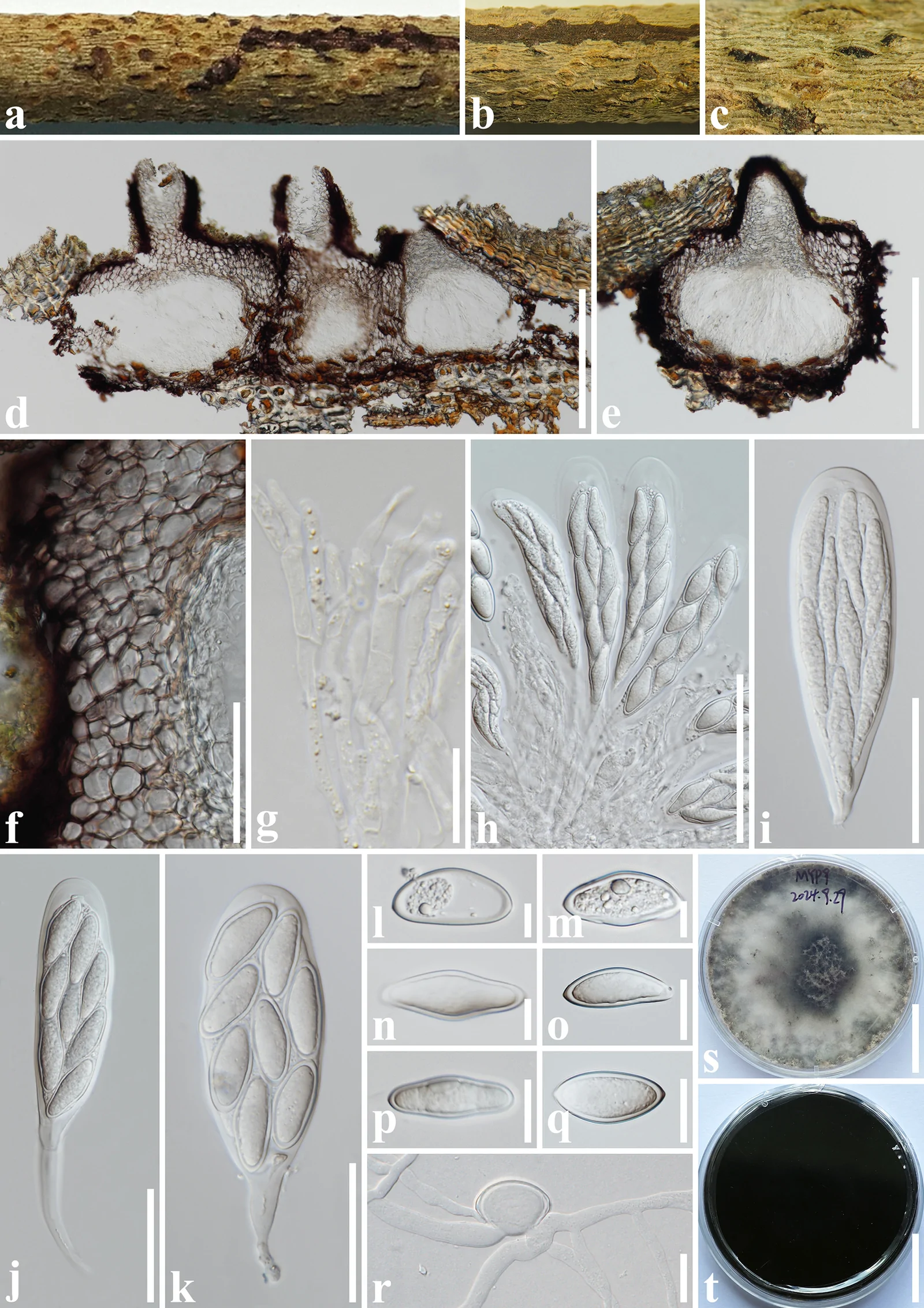
*Lasiodiplodia macadamiae* (GMB-W 1564, holotype). **a–c**. Appearance of ascomata on substrate; **d, e**. Vertical sections of ascomata; **f**. Peridium; **g**. Hamathecium; **h–k**. Asci; **l–q**. Ascospores; **r**. Germinated ascospore; **s, t**. Culture on PDA from above (**s**) and below (**t**). Scale bars: 200 μm (**d, e**); 100 μm (**h**); 50 μm (**f, i–k**); 20 μm (**g, o–r**); 10 μm (**l, m**); 2 cm (**s, t**).

##### Diagnosis.

*Lasiodiplodia macadamiae* differs from *L. chinensis* by having wider asci and ascospores; from *L. gonubiensis*, *L. lignicola*, and *L. pseudotheobromae* by having hyaline ascospores; and from *L. theobromae* by having overlapping bi- to tri-seriate ascospores. It also has distinct ITS, *tef1-α*, and *tub2* sequences and a distinct phylogenetic position.

##### Holotype.

GMB-W 1564.

##### Description.

***Saprobic*** on dead twigs of *Macadamia* sp. **Sexual morph: *Ascomata*** (including ostioles) 220–320 × 340–580 µm (x̄ = 277 × 451 µm, *n* = 10), solitary or in small groups, immersed to erumpent through the host surface, appearing as black spots on the host surface, subglobose to flask-shaped, sometimes ascomata gregarious, forming small clusters, dark brown to black, ostiolate, with a papilla. ***Ostioles*** up to 55–90 µm wide, central at neck, cylindrical to papillate. ***Peridium*** 30–90 µm thin to thick-walled, with outer layers composed of dark brown cells of textura angularis, with inner layers composed of light brown cells of textura prismatica. ***Hamathecium*** composed of 3–7.5 µm wide (x̄ = 4.7 μm, *n* = 40), abundant, dense, narrowly cylindrical to filiform, hyaline, septate, branched, broad cellular pseudoparaphyses sometimes constricted at the septum. ***Asci*** 95–180 × 25–50 µm (x̄ = 131.7 × 35.5 µm, *n* = 40), 8-spored, bitunicate, fissitunicate, clavate to broadly clavate, straight to slightly curved, rounded at apex, short furcate or obtuse at pedicel, with an apical chamber. ***Ascospores*** 30–40 × 8.5–17 µm (x̄ = 34.8 × 14 µm, *n* = 40), overlapping uni- to bi-seriate, fusiform to broadly fusiform, with rounded ends, wider at middle, aseptate, hyaline, guttulate, smooth-walled. **Asexual morph**: Not observed.

##### Culture characteristics.

Ascospores germinating on PDA within 24 h at 28 °C. Colonies on PDA reaching 6 cm in diam. after 3 weeks at 28 °C, with a rough surface, filamentous, hairy, circular, and cottony; from above, gray at the margin, becoming light gray to gray and dark gray at the center; gray to black in reverse.

##### Material examined.

China • Yunnan Province, Pu’er City, on dead twigs of *Macadamia* sp., 1 August 2024, Xian Zhang, MYP9 (GMB-W 1564), holotype; ex-type living culture, GMBCC 2446, other living culture GMBCC 2447.

##### Notes.

In the phylogenetic analyses, the isolates (GMBCC 2446 and GMBCC 2447) clustered within *Lasiodiplodia*, sister to *L. clavispora* (CGMCC 3.19594 and CGMCC 3.19595) and *L. gonubiensis* (CBS 115812 and CBS 116355), with 97% MLBS and 1.00 BYPP statistical support (Fig. 4). Based on base-pair comparisons, GMBCC 2446 was compared for ITS, *tef1-α*, and *tub2* with *L. clavispora* (CGMCC 3.19594 and CGMCC 3.19595) and *L. gonubiensis* (CBS 116355 and CBS 115812) (Table 5), and the *tef1-α* gene showed more than 25 bp differences from *L. clavispora* and *L. gonubiensis* strains. Morphologically, *L. clavispora* has only been recorded in its asexual morph, whereas *L. macadamiae* is recorded from its sexual morph; therefore, comparing these species morphologically was not possible. In this genus, only five species (viz. *L. chinensis*, *L. gonubiensis*, *L. lignicola*, *L. pseudotheobromae*, and *L. theobromae*) have been recorded from their sexual morphs (Phillips et al. 2013; Trakunyingcharoen et al. 2015; Dou et al. 2017; Tennakoon et al. 2017). *Lasiodiplodia macadamiae* resembles other *Lasiodiplodia* species in having dark brown to black ascomata, bitunicate, clavate asci, and overlapping ascospores. The isolates can be distinguished from *L. chinensis* (HMAS247143, holotype) by having a peridium composed of cells of textura angularis and textura prismatica, fissitunicate and wider asci without a furcate pedicel (95–180 × 25–50 µm vs. 53–90(–116) × 14–23(–25) µm), and overlapping uni- to bi-seriate, wider ascospores (30–40 × 8.5–17 µm vs. 19–25 × 6–9 μm), whereas *L. chinensis* has solitary ascomata or ascomata in small groups of up to four, a peridium composed of textura angularis cells, asci with a thick endotunica, and overlapping bi- to tri-seriate ascospores (Dou et al. 2017).

**Table 5.** Nucleotide comparisons of *L. macadamiae* (GMBCC 2446, ex-type) with *L. clavispora* (CGMCC 3.19594, ex-type, and CGMCC 3.19595) and *L. gonubiensis* (CBS 116355 and CBS 115812, ex-type), based on ITS, *tef1-α*, and *tub2*. All comparisons excluded gaps.

**Taxa**	**ITS**	***tef1-α***	***tub2***
*L. clavispora* (CGMCC 3.19594)	0/485 bp	31/245 bp (12.65%)	3/377 bp (0.8%)
*L. clavispora* (CGMCC 3.19595)	0/485 bp	31/245 bp (12.65%)	3/377 bp (0.8%)
*L. gonubiensis* (CBS 116355)	0/528 bp	29/242 bp (12%)	5/422 bp (1.2%)
*L. gonubiensis* (CBS 115812)	0/528 bp	29/242 bp (12%)	5/422 bp (1.2%)

*Lasiodiplodia macadamiae* differs from *L. gonubiensis* (PREM 58127, holotype) by having 8-spored, short-stalked asci and aseptate, hyaline ascospores, whereas *L. gonubiensis* has asci containing (4–)6–8 ascospores and ascospores that rarely become 1–2-septate with age and turn pale brown, becoming 1–2-septate within the ascoma or shortly after discharge (Trakunyingcharoen et al. 2015). It differs from *L. lignicola* (MFLU 12-0750, holotype) by having immersed to erumpent ascomata, asci with a short, furcate or obtuse pedicel, longer asci (95–180 × 25–50 µm vs. 80–90 × 15–25 μm), and overlapping uni- to bi-seriate, hyaline ascospores, whereas *L. lignicola* has semi-immersed ascomata, asci with a short and narrow pedicel, and uniseriate or partially overlapping, reddish-brown to dark brown ascospores (Phillips et al. 2013). It differs from *L. pseudotheobromae* (CBS-H 19916, holotype) by having a branched hamathecium, short-stalked asci, and hyaline, overlapping bi- to tri-seriate ascospores, whereas *L. pseudotheobromae* has cylindric-clavate asci and ellipsoidal to fusiform ascospores that are initially hyaline to pale yellowish and become golden to dark brown at maturity (Tennakoon et al. 2017). It differs from *L. theobromae* (CBS H-21411, neotype) by having clavate to broadly clavate, non-stipitate asci and overlapping bi- to tri-seriate ascospores, whereas *L. theobromae* has stipitate asci and irregularly biseriate ascospores (Phillips et al. 2013). Therefore, *Lasiodiplodia macadamiae*, collected from *Macadamia* sp. in China, is introduced here as a new species in *Lasiodiplodia*.

#### Lasiodiplodia ponkanicola

Taxon classificationFungiBotryosphaerialesBotryosphaeriaceae

X.E. Xiao, Crous & H.Y. Li, in Xiao, Wang, Crous, Wang, Jiao, Huang, Pu, Zhu & Li, Persoonia 47: 128 (2021)

FDD48B20-70D0-52F8-9C39-7B74FD9DBC21

840685

Fig. 4

##### Description.

For the original description, see Xiao et al. (2021).

##### Known hosts.

*Citrus reticulata* (Xiao et al. 2021), *Macadamia* sp. (this study).

##### Known distribution.

China (Zhejiang Province; Xiao et al. 2021), Thailand (this study).

##### Material examined.

Thailand • Chiang Mai Province, Samoeng Tai, on dead twigs of *Macadamia* sp. (18°85'38"N, 98°73'0"E, 512 m), 24 May 2024, Xian Zhang, TCSM58 (CMUB 40122); living culture, SDBR-CMU875.

##### Notes.

Xiao et al. (2021) introduced *Lasiodiplodia ponkanicola* as a pathogen associated with the trunk of *Citrus reticulata* cv. Ponkan and confirmed its pathogenicity with a pathogenicity test. The morphological characteristics of the isolate (CMUB 40122) resemble those of *L. ponkanicola* (ZJUE H-0044, holotype), with holoblastic, hyaline conidiogenous cells and hyaline, aseptate, ellipsoid to ovoid conidia (Xiao et al. 2021). In the phylogenetic analysis, the isolate clustered with *L. ponkanicola* (CGMCC 3.20388), with 78% ML bootstrap support (Fig. 4). Based on nucleotide comparisons, the isolate (SDBR-CMU875) showed similarities to *L. ponkanicola* (CGMCC 3.20388), with 1/433 bp (0.23%, without gaps) differences in *tub2*, 2/292 bp (0.68%, without gaps) differences in *tef1-α*, and identical ITS sequences. Therefore, based on morphological and phylogenetic analyses, the saprobic isolate of *Lasiodiplodia ponkanicola* is reported here as a new life mode (saprobic), a new host record, and a new country record, collected from *Macadamia* sp. in Thailand.

#### Lasiodiplodia endophytica

Taxon classificationFungiBotryosphaerialesBotryosphaeriaceae

N.I. de Silva, A.J.L. Phillips & K.D. Hyde, in de Silva, Scientific Reports 9 (no. 14355): 4 (2019)

62FFE32C-19B7-58C9-8BF5-5ACCE0917AED

556218

Figs 4, 15

##### Description.

***Saprobic*** dead twigs of *Macadamia* sp. **Asexual morph: *Conidiomata*** 400–565 × 150–250 µm (x̄ = 483 × 195 µm, *n* = 10), pycnidial, immersed to semi-immersed, scattered to aggregated, unilocular or multilocular, brown to dark brown, globose to depressed globose, ostiolate. ***Conidiomata walls*** 20–60 µm wide, composed of hyaline to brown to dark brown cells of textura angularis. ***Paraphyses*** 3–6.5 μm wide arising amongst the conidiogenous cells, hyaline, cylindrical, the basal cells often slightly swollen, rounded at apex. ***Conidiophores*** absent. ***Conidiogenous cells*** 2.0–5.5 µm diam., hyaline, discrete, cylindrical, holoblastic. ***Conidia*** 19.5–32.5 × 10–17 (x̄ = 26.5 × 13.6 µm, *n* = 30) μm, hyaline, aseptate, ellipsoidal to broadly ellipsoidal, occasionally oblong, both ends broadly rounded, smooth- and thick-walled, contents granular, small guttules.

**Figure 15.**
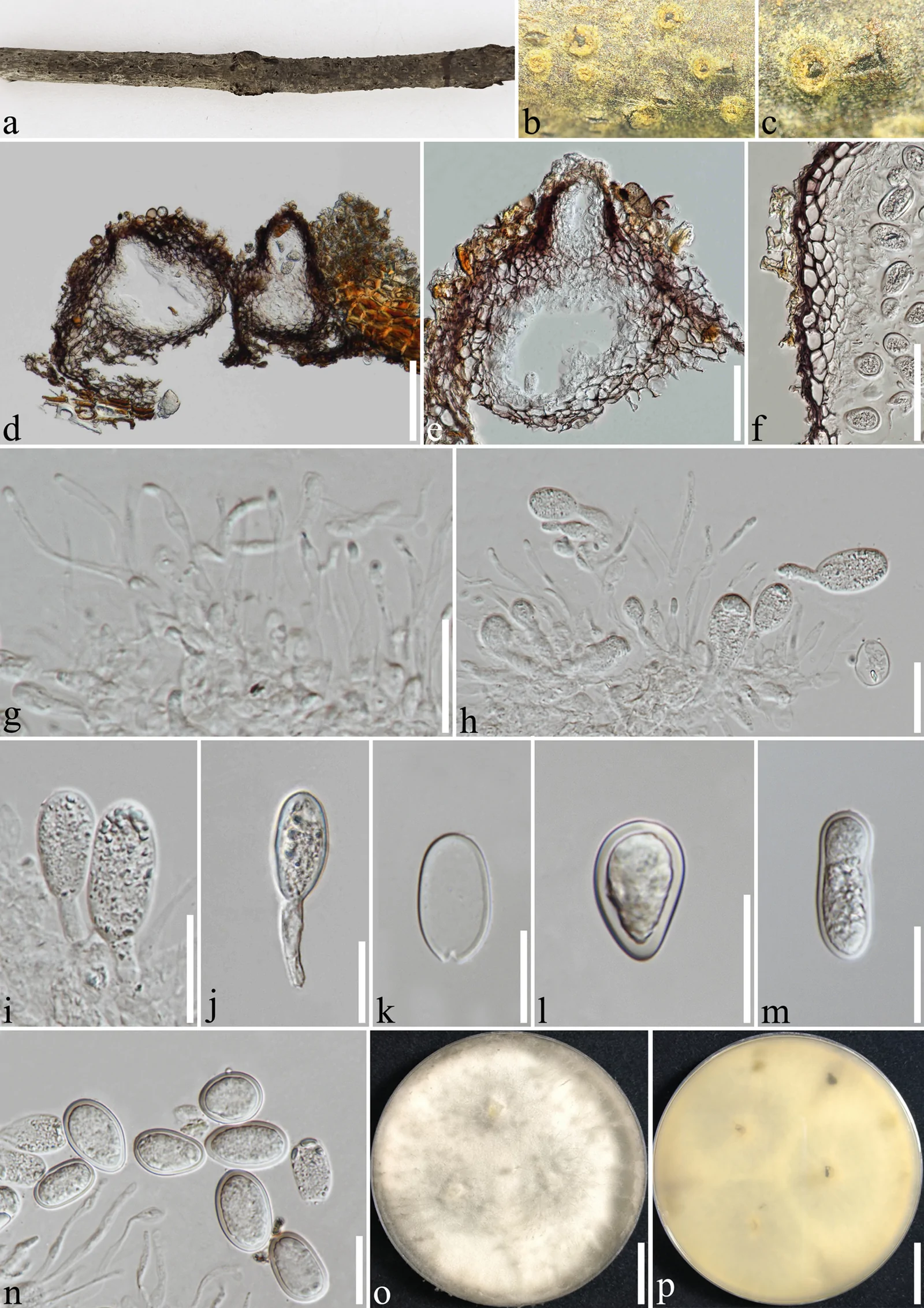
*Lasiodiplodia endophytica* (CMUB 40123, new host record and country record). **a–c**. Conidiomata on a decaying twig of *Macadamia* sp.; **d, e**. Vertical sections of conidioma; **f**. Conidiomata wall; **g**. Paraphyses; **h–j**. Conidiogenous cells with conidia; **k–n**. Conidia; **o, p**. Culture on PDA from above (**o**) and below (**p**). Scale bars: 100 μm (**d**); 50 μm (**e, f**); 20 μm (**g–n**); 2 cm (**o, p**).

##### Known hosts.

*Macadamia* sp. (this study), *Magnolia candolii* (de Silva et al. 2019).

##### Known distribution.

China (Yunnan Province; de Silva et al. 2019), Thailand (this study).

##### Material examined.

Thailand • Chiang Mai Province, Samoeng Tai, on dead twigs of *Macadamia* sp. (18°85'38"N, 98°73'0"E, 512 m), 24 May 2024, Xian Zhang, TCSM53 (CMUB 40123); living culture, SDBR-CMU876.

##### Notes.

de Silva et al. (2019) introduced *Lasiodiplodia endophytica*. *Lasiodiplodia endophytica* has been reported as an endophyte on fresh leaves of *Magnolia candolii* (de Silva et al. 2019). The morphological characteristics of the isolates resemble those of *L. hyalina* (HMAS 255216, holotype) and *Lasiodiplodia thailandica* (CBS-H 21933, holotype) in having solitary conidiomata, holoblastic, discrete, hyaline conidiogenous cells, and thick-walled, aseptate conidia with both ends broadly rounded. However, they can be distinguished from *L. hyalina* (HMAS 255216, holotype) by having unilocular or multilocular conidiomata and ellipsoidal to broadly ellipsoidal conidia, and from *L. thailandica* (CBS-H 21933, holotype) by having immersed to semi-immersed conidiomata and ellipsoidal to broadly ellipsoidal conidia (Trakunyingcharoen et al. 2015; Dou et al. 2017). In the phylogenetic analysis, the isolates clustered with *L. endophytica* (MFLUCC 18-1121), with 73% ML bootstrap support (Fig. 4). Based on nucleotide comparisons, the isolates showed similarity to *L. hyalina* strain CGMCC 3.17975, with 1/353 bp (0.28%, without gaps) difference in *tub2* and identical ITS and *tef1-α* sequences. Therefore, based on morphological and phylogenetic analyses, *Lasiodiplodia endophytica* is introduced here as a new host record and a new country record, collected from *Macadamia* sp. in Thailand.

#### Palmiascoma macadamiae

Taxon classificationFungiPleosporalesBambusicolaceae

X. Zhang, Tibpromma & Karun. sp. nov.

5FC70F32-675B-5304-A219-A42A9220C165

863026

Figs 5, 16

##### Etymology.

“*macadamiae*” refers to the host plant genus *Macadamia*.

**Figure 16.**
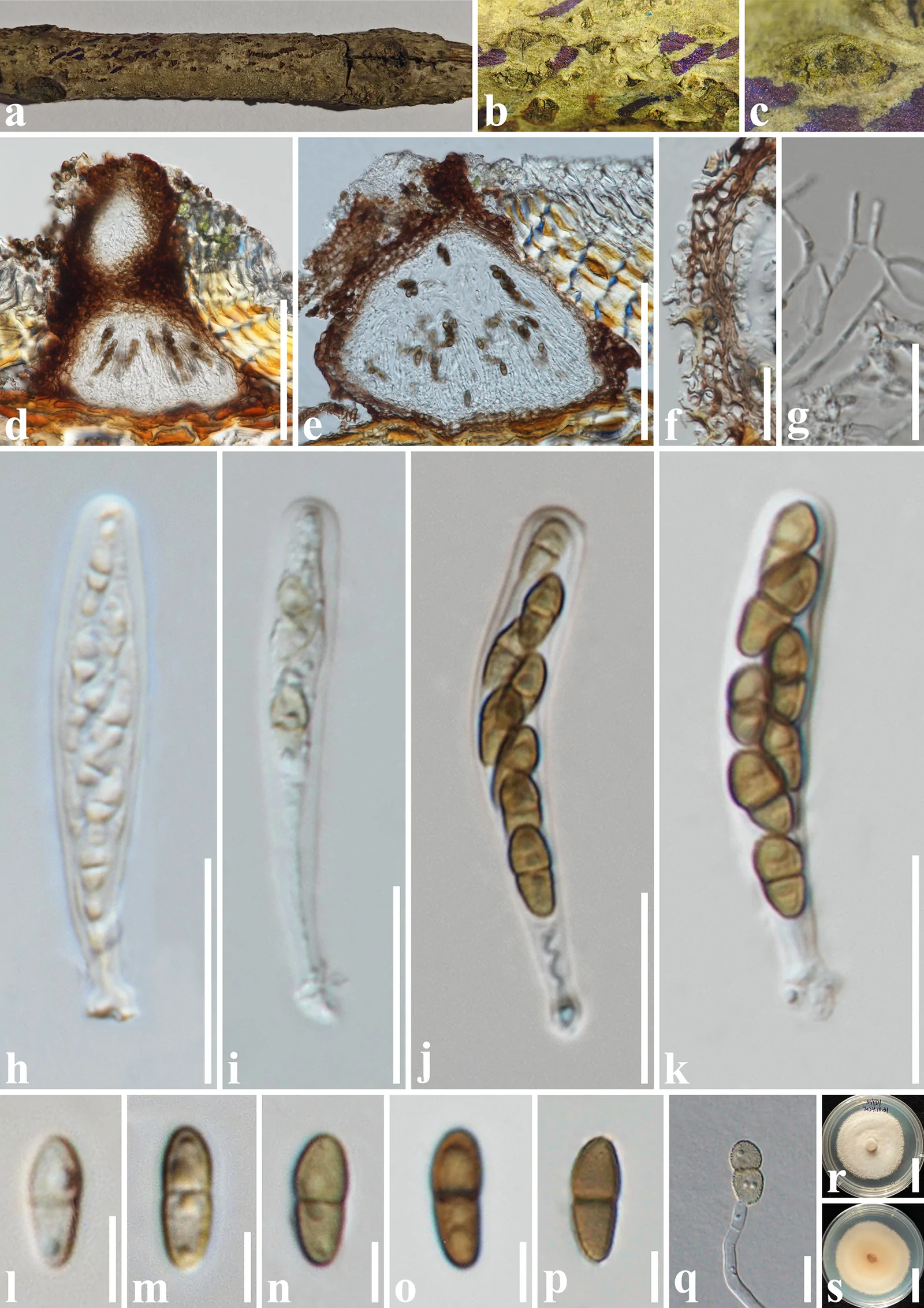
*Palmiascoma macadamiae* (GMB-W 1565, holotype). **a–c**. Appearance of ascomata on substrate; **d, e**. Vertical sections of ascomata; **f**. Peridium; **g**. Hamathecium; **h–k**. Asci; **l–p**. Ascospores; **q**. Germinated ascospore; **r, s**. Culture on PDA from above (**r**) and below (**s**). Scale bars: 100 μm (**d, e**); 20 μm (**f–k**); 10 μm (**q**); 5 μm (**l–p**); 2 cm (**r, s**).

##### Diagnosis.

*Palmiascoma macadamiae* differs from *P. acericola* by having overlapping 1–2-seriate ascospores, from *P. gregariascomum* by lacking a mucilaginous sheath or appendages on the ascospores, and from *P. qujingense* by having non-echinulate, slightly shorter ascospores. It also has distinct nrLSU, nrSSU, ITS, and *rpb2* sequences and a distinct phylogenetic position.

##### Holotype.

GMB-W 1565.

##### Description.

***Saprobic*** on dead twigs of *Macadamia* sp. **Sexual morph: *Ascomata*** 115–205 × 145–265 µm (x̄ = 159 × 198 µm, *n* = 10), scattered to clustered, semi-immersed, slightly erumpent, dark spots on the host surface, globose to subglobose or irregular, glabrous, uniloculate, centrally ostiolate, papillate. ***Ostioles*** 40–150 µm long, 25–60 µm diam. (x̄ = 80 × 41 µm, *n* = 10), brown to dark brown, glabrous, papillate, shiny, lined with hyaline periphyses. ***Peridium*** 10–30 µm thin to thick-walled, of unequal thickness, slightly thick at sides, composed of brown to dark brown cells of textura angularis. ***Hamathecium*** 1.5–3.0 µm wide (x̄ = 2.2 μm, *n* = 30), abundant, dense, narrowly cylindrical to filiform, hyaline, branched, pseudoparaphyses; no distinct septation was observed, anastomosing above the asci, embedded in a mucilaginous matrix. ***Asci*** 40–60 × 7.5–11.5 µm (x̄ = 49.5 × 8.7 µm, *n* = 30), 8-spored, bitunicate, fissitunicate, cylindric-clavate, short pedicellate, with a furcate base, apically rounded, with an ocular chamber. ***Ascospores*** 8.5–12 × 4–5 µm (x̄ = 10.5 × 4.5 µm, *n* = 30), overlapping 1–2-seriate, fusiform to ellipsoid, with rounded ends, hyaline to slight brown to brown, straight to slightly curved, uniseptate, with slightly constricted at the septum, upper part slight wider, with or without guttules, without mucilaginous sheath or appendages, rough-walled. **Asexual morph**: Not observed.

##### Culture characteristics.

Ascospores germinating on PDA within 24 h at 28 °C, with colonies on PDA reaching 3.6 cm in diam. after 3 weeks at 28 °C, with a rough surface, filamentous, hairy, circular, cottony, and white from above; light yellow at the margins, turning light brown toward the center in reverse.

##### Materials examined.

China • Yunnan Province, Dehong City, on dead twigs of *Macadamia* sp., 24 April 2024, Xian Zhang, MYD1 (GMB-W 1565), holotype; ex-type living culture, GMBCC 2448, other living culture GMBCC 2449.

##### Notes.

In the phylogenetic analyses, the isolates (GMBCC 2448 and GMBCC 2449) clustered within *Palmiascoma*, sister to *P. acericola* (CGMCC 3.24963 and UESTCC 23.0018), *P. gregariascomum* (MFLUCC 11-0175 and MFLUCC 11-0424), and *P. qujingense* (KKUMCC 19-0201), with 100% MLBS and 1.00 BYPP statistical support (Fig. 5). Morphologically, *Palmiascoma macadamiae* resembles *P. acericola*, *P. gregariascomum*, and *P. qujingense* in having glabrous, uniloculate, papillate ascomata, a hamathecium containing hyaline, septate, branched pseudoparaphyses, 8-spored, bitunicate, fissitunicate, cylindric-clavate, apically rounded asci, and fusiform to ellipsoid, uniseptate, rough-walled ascospores (Liu et al. 2015; Monkai et al. 2021; Li et al. 2023). However, *P. macadamiae* can be distinguished from *P. acericola* (HKAS 127160, holotype) by having scattered to clustered, semi-immersed, subglobose or irregular ascomata, pseudoparaphyses without distinct septation, short-pedicellate asci, and overlapping 1–2-seriate, slightly shorter (8.5–12 × 4–5 µm vs. 12–14 × 3.5–4.5 μm) ascospores, whereas *P. acericola* has immersed to erumpent ascomata and elongated, cylindrical, pedicellate asci (Li et al. 2023). It can be distinguished from *P. gregariascomum* (MFLU 11-0211, holotype) by having a hamathecium without distinctly observed septation and ascospores that are hyaline when young, slightly constricted at the septum, and without a mucilaginous sheath or appendages, whereas *P. gregariascomum* has a hamathecium with distinct septa and echinulate ascospores surrounded by a mucilaginous sheath (Liu et al. 2015). It can also be distinguished from *P. qujingense* (KUN-HKAS109581, holotype) by having semi-immersed ascomata, pseudoparaphyses without distinctly observed septation, slightly shorter (40–60 × 7.5–11.5 µm vs. 60–80 × 9–10 μm) asci, and non-echinulate, slightly shorter (8.5–12 × 4–5 µm vs. 12–16 × 4.5–5.5 μm) ascospores, whereas *P. qujingense* has immersed to semi-immersed ascomata and clavate to ellipsoidal, echinulate ascospores (Monkai et al. 2021). Therefore, *Palmiascoma macadamiae* is introduced here as a new species in *Palmiascoma*, collected from *Macadamia* sp. in China.

#### Palmiascoma chiangraiense

Taxon classificationFungiPleosporalesBambusicolaceae

N. Afshari, Lumyong & K.D. Hyde, in Afshari et al., Mycosphere 16: 4812 (2025)

97364F88-B55D-5468-96F6-EEB50A357EA5

904739

Figs 5, 17

##### Holotype.

MFLU 24-0459.

**Figure 17.**
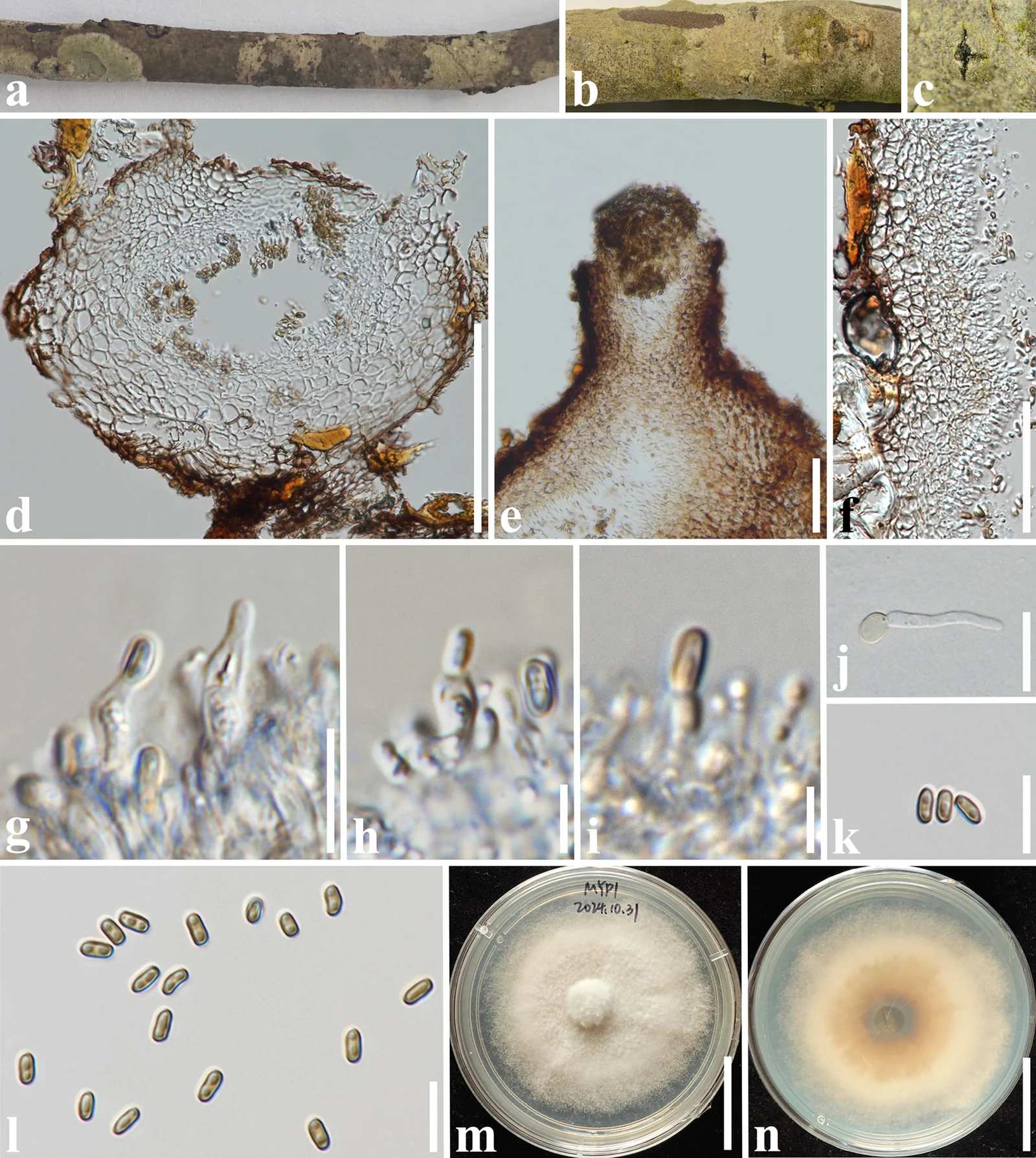
*Palmiascoma chiangraiense* (GMB-W 1566, new host record and country record). **a–c**. Conidiomata on a decaying branch of *Macadamia* sp.; **d**. Vertical section of conidioma; **e**. Vertical section through conidioma showing the ostiolar neck; **f**. Conidiomata wall; **g–i**. Conidiogenous cells with conidia; **j**. Germinated conidium; **k, l**. Conidia; **m, n**. Culture on PDA from above (**m**) and below (**n**). Scale bars: 50 μm (**d–f**); 20 μm (**j**); 10 μm (**g, k, l**); 5 μm (**h, i**); 2 cm (**m, n**).

##### Description.

***Saprobic*** on dead twigs of *Macadamia* sp. **Sexual morph**: Not observed. **Asexual morph: *Conidiomata*** 225–380 × 335–900 µm (x̄ = 299 × 634 µm, *n* = 10), pycnidial, immersed to semi-immersed or erumpent, solitary or aggregated, globose to subglobose, irregular, glabrous, ostiole, uniloculate, scattered, papillate. ***Conidiomata walls*** 25–75 µm (x̄ = 45 µm, *n* = 30) thin to thick walled, composed of several layers of hyaline to dark brown, outer layers composed of 13–37 µm (x̄ = 22.6 µm, *n* = 15), brown to dark brown cells of textura angularis, inner layers composed of 18–47 µm (x̄ = 30.8 µm, *n* = 15), hyaline cells, organized in a textura angularis. ***Conidiophores*** arising from basal cavity of conidiomata, mostly reduced to conidiogenous cells. ***Conidiogenous cells*** holoblastic, phialidic, ampulliform to cylindrical, hyaline, aseptate, smooth-walled, guttulate. ***Conidia*** 4–5.5 × 1–3 µm (x̄ = 4.8 × 2.3 µm, *n* = 30), solitary, oblong to ellipsoidal, aseptate, with rounded or obtuse ends, hyaline when young, becoming brown with maturity, smooth-walled, guttulate.

##### Culture characteristics.

Conidia germinating on PDA within 24 h at 28 °C, with colonies on PDA reaching 6 cm in diam. after 3 weeks at 28 °C, with a rough surface, filamentous, hairy, circular, cottony, and white from above; light brown at the margins, turning yellow-brown to brown toward the center in reverse.

##### Known hosts.

*Dipterocarpus alatus* (Afshari et al. 2025b), *Macadamia* sp. (this study).

##### Known distribution.

China (Yunnan Province; this study), Thailand (Afshari et al. 2025b).

##### Material examined.

China • Yunnan Province, Pu’er City, on dead twigs of *Macadamia* sp., 1 August 2024, Xian Zhang, MYP1 (GMB-W 1566); living culture, GMBCC 2450, other living culture GMBCC 2451.

##### Notes.

*Palmiascoma chiangraiense* was introduced by Afshari et al. (2025b), isolated from dead wood of *Dipterocarpus alatus* based on its sexual morph. In this study, the asexual morph of this species was discovered for the first time. In the phylogenetic analyses, the isolate (GMBCC 2450) clustered with *P. chiangraiense* strain MFLUCC 24-0546, with 100% MLBS and 1.00 BYPP statistical support (Fig. 5). Morphologically, the isolate (GMBCC 2450) resembles *P. gregariascomum* in having erumpent, pycnidial conidiomata, phialidic, discrete, ampulliform to cylindrical conidiogenous cells, and solitary, oblong to ellipsoidal conidia, but can be distinguished by having larger conidiomata (225–380 µm high × 335–900 µm diam. vs. 140–200 μm high × 130–220 μm diam.) (Liu et al. 2015). In addition, phylogenetic analyses show that these species form distinct lineages (Fig. 5). *Palmiascoma acericola*, *P. macadamiae*, and *P. qujingense* were recorded only from their sexual morphs. Therefore, the asexual morphological characteristics of *P. chiangraiense* could not be compared with those of the three species. Based on nucleotide comparisons, the isolate (GMBCC 2450) showed similarity to *P. chiangraiense* strain MFLUCC 24-0546, with 3/644 bp (0.47%, without gaps) differences in ITS, 2/836 bp (0.24%, without gaps) differences in nrLSU, 4/1033 bp (0.39%, without gaps) differences in *rpb2*, and identical nrSSU sequences. Therefore, the collection is introduced here as a new host record and a new country record of *P. chiangraiense*, collected from *Macadamia* sp. in China.

#### Crassiparies quadrisporus

Taxon classificationFungiPleosporalesNeohendersoniaceae

M. Matsum., K. Hiray. & Kaz. Tanaka, in Li et al., Fungal Diversity 78: 63 (2016)

F83AB3CF-223F-5C4A-949B-7BD9D5F5A746

559359

Fig. 6

##### Description.

For the original description, see Li et al. (2016).

##### Known hosts.

*Acer* sp. (Li et al. 2016), *Macadamia* sp. (this study), *Machilus japonica* (Tanaka et al. 2017), *Hevea brasiliensis* (Senwanna et al. 2021).

##### Known distribution.

China (Yunnan Province; this study), Japan (Li et al. 2016; Tanaka et al. 2017), Thailand (Senwanna et al. 2021).

##### Material examined.

China • Yunnan Province, Dehong City, on dead twigs of *Macadamia* sp., 24 April 2024, Xian Zhang, MYD3 (GMB-W 1567); living culture, GMBCC 2452, • ibid., MYD9 other living culture GMBCC 2453.

##### Notes.

Li et al. (2016) introduced *Crassiparies quadrisporus*, isolated from dead twigs of *Acer* sp. The morphological characteristics of the isolates resemble those of *Crassiparies quadrisporus* (HHUF 30409, holotype) in having bitunicate, fissitunicate, pedicellate, cylindric-clavate or clavate asci and overlapping biseriate, hyaline, septate, fusiform ascospores (Li et al. 2016; Tanaka et al. 2017; Senwanna et al. 2021). In the phylogenetic analysis, the isolates clustered with *Crassiparies quadrisporus* strains (MAFF 245408, MAFF 246250, MFLUCC 18-0298, and MFLUCC 21-0148) with 100% MLBS and 1.00 BYPP statistical support (Fig. 6). Based on nucleotide comparisons, the isolate GMBCC 2452 was highly similar to *Crassiparies quadrisporus* (MAFF 245408), with only 3/927 bp (0.3%, without gaps) differences in *tef1-α*, and was identical in ITS, nrLSU, nrSSU, and *rpb2*. Therefore, based on morphological and phylogenetic analyses, *Crassiparies quadrisporus* is introduced here as a new host and country record, collected from *Macadamia* sp. in China.

#### Chromolaenicola ananasi

Taxon classificationFungiPleosporalesDidymosphaeriaceae

X.G. Tian, K.D. Hyde & Tibpromma, in Tian et al., Mycosphere 15(1): 31 (2024)

C7E18628-CD3F-5C41-B2F8-997643FAA8A7

900971

Figs 7, 18

##### Description.

***Saprobic*** on dead twigs of *Macadamia* sp. **Sexual morphs**: Ascomata 230–415 µm high × 200–415 µm diam. (x̄ = 332 × 309 µm, *n* = 10, including ostioles), solitary to scattered, immersed or erumpent, scattered, dark brown to black, globose to sub-globose, rarely irregular, unilocular to bilocular, with a central ostiole. ***Peridium*** 18–40 µm wide, thin-walled, composed of several layers, inner layers comprising hyaline cells of textura epidermoidea, outer layers comprising brown to pale brown cells of textura angularis. ***Hamathecium*** composed of 1.5–3.0 µm wide (x̄ = 1.9 μm, *n* = 40), cylindrical to filiform, hyaline, septate, branched, pseudoparaphyses. ***Asci*** 70–110 × 5.5–9 µm (x̄ = 90 × 7.0 µm, *n* = 40), 8-spored, bitunicate, cylindrical-clavate, straight to slightly curved, rounded at the apex, short pedicellate with club-shaped base. ***Ascospores*** 10–14 × 5.0–6.5 µm (x̄ = 12.5 × 5.4 µm, *n* = 40), slightly overlapping, uniseriate, fusiform to broadly fusiform, 1-septate at center, constricted at septum, with wider upper cell, straight or slightly curved, initially hyaline to pale brown, becoming reddish brown to brown at maturity with thickened septum, with or without guttules, without a gelatinous sheath. **Asexual morph**: See Tian et al. (2024b).

**Figure 18.**
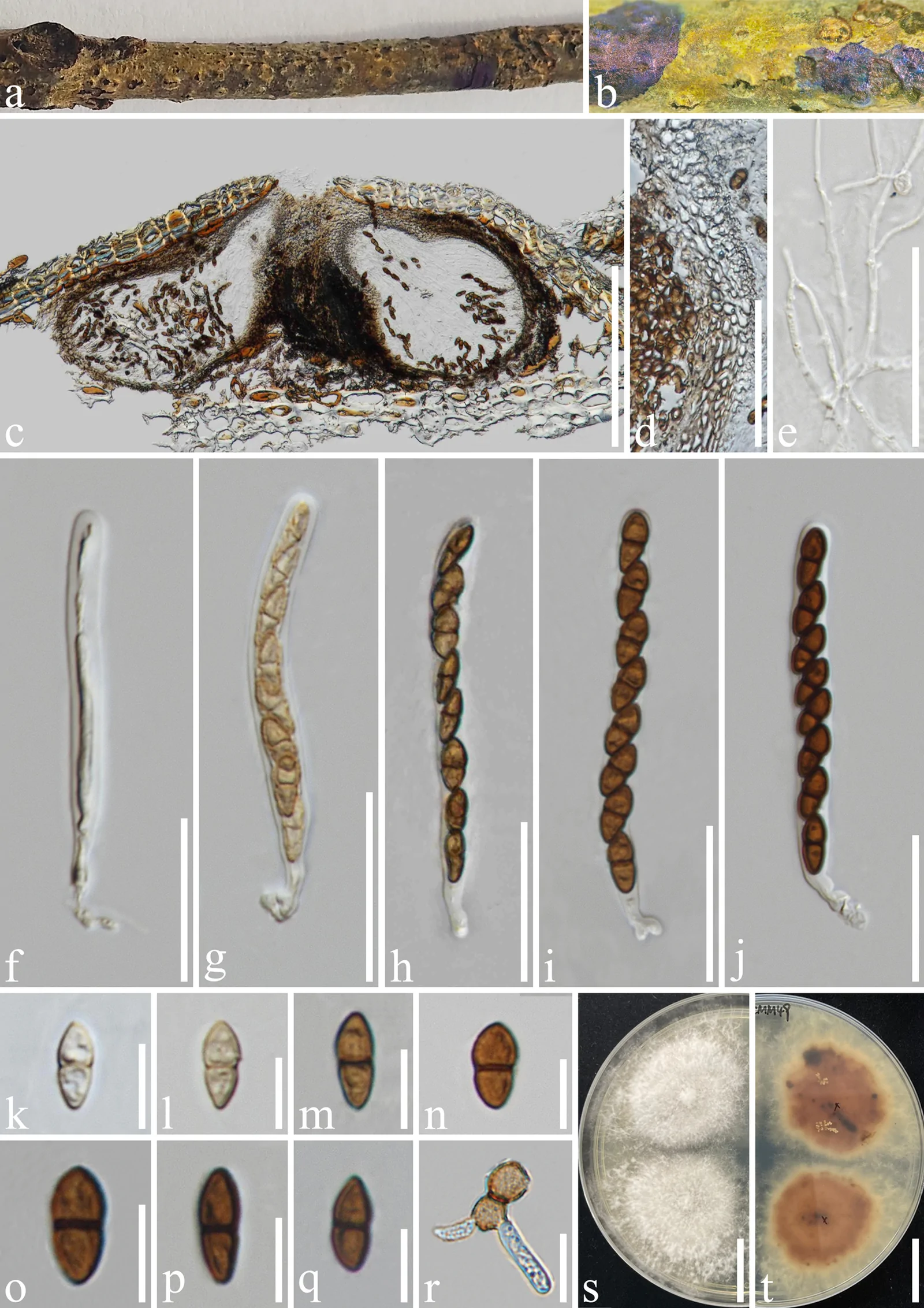
*Chromolaenicola ananasi* (CMUB 40120, new host record and country record). **a, b**. Appearance of ascomata on substrate; **c**. Vertical section of ascomata; **d**. Peridium; **e**. Hamathecium; **f–j**. Asci; **k–q**. Ascospores; **r**. Germinated ascospore; **s, t**. Culture on PDA from above (**s**) and below (**t**). Scale bars: 200 μm (**c**); 50 μm (**d, e**); 30 μm (**f–j**); 20 μm (**r**); 10 μm (**k–q**); 2 cm (**s, t**).

##### Known hosts.

*Ananas comosus* (Tian et al. 2024b), *Macadamia* sp. (this study).

##### Known distribution.

Thailand (Tian et al. 2024b; this study).

##### Material examined.

Thailand • Chiang Mai Province, Doi Saket District, 18°52'43"N, 99°13'15"E, 384 m elevation, on dead twigs of *Macadamia* sp., 24 November 2023, Xian Zhang, TCMM49 (CMUB 40120); living culture, SDBR-CMU872.

##### Notes.

*Chromolaenicola ananasi* was introduced by Tian et al. (2024b), isolated from dead leaves of *Ananas comosus* based on its asexual morph. In the present study, the sexual morph of this species was discovered for the first time. In the phylogenetic analysis, the isolate SDBR-CMU872 grouped with *Chromolaenicola ananasi* (MFLU 23-0167 and MFLUCC 23-0111) with 100% MLBS and 1.00 BYPP statistical support (Fig. 7). Morphologically, the collection is almost identical to *Chromolaenicola sapindi* (HKAS 122789, holotype) in having immersed-to-erumpent ascomata, 8-spored, bitunicate, cylindric-clavate asci, and uniseriate ascospores that are initially hyaline to pale brown, becoming reddish brown to brown at maturity with a thickened septum, constricted at the central septum, and lacking a gelatinous sheath (Ren et al. 2022). However, the asci and ascospores of the collection are slightly different from those of *C. sapindi*. The collection has a peridium composed of cells of textura epidermoidea and textura angularis, short-pedicellate asci with a club-shaped base, and ascospores that are 1-septate at the center from immature to mature stages, whereas *C. sapindi* has ampulliform or obovoid ascomata and ellipsoidal, aseptate or 1-septate ascospores; the phylogenetic tree also shows that they are distinct species (Ren et al. 2022). Based on nucleotide comparisons, the isolate SDBR-CMU872 showed similarity to *C. ananasi* (KUMCC 21-0564 and KUMCC 21-0594), with only 7/902 bp (0.8%, without gaps) differences in *tef1-α*, and was identical in ITS, nrLSU, and nrSSU. The collection is recognized as *C. ananasi* because it shares almost identical molecular data with other collections (Tian et al. 2024b). Therefore, based on morphological and phylogenetic analyses, *Chromolaenicola ananasi* is introduced here as a new host and country record, collected from *Macadamia* sp. in Thailand. In addition, this is the first report of *Chromolaenicola ananasi* associated with *Macadamia* sp.

#### Montagnula donacina

Taxon classificationFungiPleosporalesDidymosphaeriaceae

(Niessl) Wanas., E.G. Jones & D. Hyde, Fungal Biology 120 (11): 1365 (2016)

681B352B-5D79-5C2D-BBEB-0B9B72E7ADAC

626421

Fig. 8

##### Description.

For description, see Sun et al. (2023).

##### Known hosts.

*Acacia reficiens* (Aptroot 1995), *Acacia* sp. (Aptroot 1995), *Adhatoda vasica* (Aptroot 1995), *Ailanthus altissima* (Aptroot 1995), *Althaea rosea* (Aptroot 1995), *Annona squamosa* (Aptroot 1995), *Arundo donax* (Aptroot 1995), *Bambusoideae* sp. (Aptroot 1995), *Cajanus cajan* (Aptroot 1995), *Calamus australis* (Hyde et al. 1999), *Careya arborea* (Aptroot 1995), *Chromolaena odorata* (Aptroot 1995), *Citrus aurantiifolia* (Aptroot 1995), *Citrus* sp. (Aptroot 1995), *Clerodendrum infortunatum* (Aptroot 1995), *Clerodendrum multiflorum* (Aptroot 1995), *Coffea arabica* var. *catimor* (Aptroot 1995), *Coffea arabica* (Aptroot 1995), *Coffea robusta* (Aptroot 1995), *Craterellus odoratus* (Zhao et al. 2018), *Duranta repens* (Aptroot 1995), *Ficus glomerata* (Aptroot 1995), *Funtumia africana* (Aptroot 1995), *Hevea brasiliensis* (Aptroot 1995), *Hibiscus* sp. (Aptroot 1995), *Ipomoea carnea* (Aptroot 1995), *Lagerstroemia* sp. (Aptroot 1995), *Litchi litchi* (Thaung 2008), *Macadamia* sp. (this study), *Mallotus philippinensis* (Aptroot 1995), *Morus alba* (Aptroot 1995), *Nerium odorum* (Aptroot 1995), *Paeonia suffruticosa* (Li et al. 2023), *Phyllostachys bambusoides* (Wang et al. 2004), *Pistacia* sp. (Aptroot 1995; Wang et al. 2004), *Premna cumingiana* (Aptroot 1995), *Pseudosasa japonica* (Aptroot 1995), *Saccharum officinarum* (Aptroot 1995), *Tectona grandis* (Aptroot 1995), *Terminalia tomentosa* (Aptroot 1995), *Trachycarpus fortune* (Hyde et al. 1999), unidentified twig (Aptroot 1995), *Vitis vinifera* (Pitt et al. 2014), *Wikstroemia* sp. (Aptroot 1995), *Zea mays* (Aptroot 1995), dead wood (Ren et al. 2022; Sun et al. 2023; Wanasinghe et al. 2024).

##### Known distribution.

Australia (Pitt et al. 2014), Brazil (Aptroot 1995), Central African Republic (Aptroot 1995), China (Aptroot 1995; Hong Kong, Hyde et al. 1999; Sichuan Province, Li et al. 2023; Yunnan Province, Zhao et al. 2018; Wanasinghe et al. 2024; this study), Colombia (Aptroot 1995), France (Aptroot 1995), Georgia (Aptroot 1995), India (Aptroot 1995), Japan (Wang et al. 2004), Myanmar (Thaung 2008), Namibia (Aptroot 1995), Papua New Guinea (Aptroot 1995), Paraguay (Aptroot 1995), Philippines (Aptroot 1995), Portugal (Aptroot 1995), Sierra Leone (Aptroot 1995), Thailand (Ren et al. 2022), USA (Aptroot 1995; Wang et al. 2004).

##### Material examined.

China • Yunnan Province, Honghe City, on a dead branch of *Macadamia* sp., 16 July 2024, Xian Zhang, MYH4 (GMB-W 1570); living culture, GMBCC 2455.

##### Notes.

Sun et al. (2023) synonymized *Montagnula chromolaenicola*, *M. puerensis*, *M. saikhuensis*, and *M. thailandica* under *M. donacina* based on morphological and phylogenetic analyses. However, Wanasinghe et al. (2024) subsequently suggested that these taxa represent distinct species based on combined gene analyses. In the phylogenetic analysis, the isolate GMBCC 2455 was sister to *M. donacina* (KUNCC 23-14428 and KUNCC 23-14429), with 81% ML bootstrap support (Fig. 8). Morphological analysis also supports the identification of the isolate as *M. donacina* (HKAS 124552), with semi-immersed ascomata, bitunicate, clavate asci with a bulbous, long pedicel, and 1-septate ascospores constricted at the septum (Sun et al. 2023). Therefore, based on this evidence, the isolate is confirmed as *Montagnula donacina*, and this species is reported for the first time from *Macadamia* sp. in China.

#### Montagnula thailandica

Taxon classificationFungiPleosporalesDidymosphaeriaceae

Mapook & K.D. Hyde, in Mapook, Hyde, McKenzie, Jones, Bhat, Jeewon, Stadler, Samarakoon, Malaithong, Tanunchai, Buscot, Wubet & Purahong, Fungal Diversity 101: 35 (2020)

9736D64B-96BF-5FAD-8F69-BDB042F6AF7C

557299

Fig. 8

##### Description.

For description, see Mapook et al. (2020).

##### Known hosts.

*Chromolaena odorata* (Mapook et al. 2020), *Coffea arabica* var. *catimor* (Lu et al. 2022), *Hevea brasiliensis* (Senwanna et al. 2021), *Macadamia* sp. (this study), unidentified twig (Boonmee et al. 2021).

##### Known distribution.

##### Material examined.

China • Yunnan Province, Pu’er City, on dead twigs of *Macadamia* sp., 1 August 2024, Xian Zhang, MYP5 (GMB-W 1569); living culture, GMBCC 2454.

##### Notes.

Mapook et al. (2020) introduced *Montagnula thailandica*, isolated from dead stems of *Chromolaena odorata*, based on morphological and phylogenetic analyses. In the phylogenetic analysis, the isolate GMBCC 2454 was sister to *M. thailandica* (MFLUCC 17-1508) (Fig. 8). Morphologically, the collection fits well with *M. thailandica* (MFLU 20-0325, holotype), with immersed to erumpent ascomata and bitunicate, fissitunicate asci with long pedicels and 1-septate ascospores constricted at the septum (Mapook et al. 2020). Therefore, based on morphological and phylogenetic analyses, the isolate is confirmed as *M. thailandica* and is introduced as a new host and country record from *Macadamia* sp. in China.

#### Pseudochaetosphaeronema chiangraiense

Taxon classificationFungiPleosporalesMacrodiplodiopsidaceae

Wijes., Boonmee & K.D. Hyde, in Boonmee et al., Fungal Diversity 111: 75 (2021)

BEBE40D8-1BDF-5020-A5B3-47E6CB7F00A4

558549

Fig. 9

##### Description.

For the original description, see Boonmee et al. (2021).

##### Known hosts.

*Macadamia* sp. (this study), *Tamarindus* sp. (Boonmee et al. 2021).

##### Known distribution.

China (Yunnan Province; this study), Thailand (Boonmee et al. 2021).

##### Materials examined.

China • Yunnan Province, Dehong City, on a dead branch of *Macadamia* sp., 24 April 2024, Xian Zhang, MYD19 (GMB-W 1571); living culture, GMBCC 2456, other living culture GMBCC 2457.

##### Notes.

Boonmee et al. (2021) introduced *Pseudochaetosphaeronema chiangraiense*, isolated from a terrestrial branch of *Tamarindus* sp. In the phylogenetic analysis, the isolate GMBCC 2456 clustered with *Pse. chiangraiense* strains (MFLUCC 21-0083 and UESTCC 23-0065) with 62% ML bootstrap support (Fig. 9). Based on nucleotide comparisons, the isolate GMBCC 2456 showed similarity to *Pse. chiangraiense* (MFLUCC 21-0083), with only 1/842 bp (0.1%, without gaps) difference in *tef1-α*, and was identical in ITS, nrLSU, and nrSSU. Morphologically, the collection fits well with the description of *Pse. chiangraiense* (MFLU 21-0064, holotype) in having 8-spored, bitunicate asci with rounded ends and overlapping, hyaline, fusiform, septate ascospores (Boonmee et al. 2021). Therefore, based on morphological and phylogenetic analyses, the isolates are confirmed as *Pse. chiangraiense* and are introduced as a new host and country record from *Macadamia* sp. in China.

#### Pseudochaetosphaeronema puerense

Taxon classificationFungiPleosporalesMacrodiplodiopsidaceae

R.F. Xu, K.D. Hyde & Tibpromma [as ‘ puerensis’], in Manawasinghe et al., Fungal Diversity 130: 40 (2024) [2025]

42F633E9-E10D-5059-B55F-D6EF1C017D6D

901412

Fig. 9

##### Description.

For the original description, see Manawasinghe et al. (2025).

##### Known hosts.

*Hevea brasiliensis* (Manawasinghe et al. 2025), *Macadamia* sp. (this study).

##### Known distribution.

China (Yunnan Province; Manawasinghe et al. 2025; this study).

##### Material examined.

China • Yunnan Province, Dehong City, on a dead branch of *Macadamia* sp., 24 April 2024, Xian Zhang, MYD14 (GMB-W 1572); living culture, GMBCC 2458, other living culture GMBCC 2459.

##### Notes.

Manawasinghe et al. (2025) introduced *Pseudochaetosphaeronema puerense*, isolated from a dead branch of *Hevea brasiliensis* in Yunnan Province, China. In the phylogenetic analysis, the isolates GMBCC 2458 and GMBCC 2459 clustered with *Pse. puerense* strains (ZHKUCC 22-0288 and ZHKUCC 22-0289) with 100% MLBS and 1.00 BYPP statistical support (Fig. 9). Based on nucleotide comparisons, the isolate GMBCC 2458 showed similarity to *Pseudochaetosphaeronema puerense* (ZHKUCC 22-0288, ex-type), with only 2/561 bp (0.4%, without gaps) differences in ITS, 1/967 bp (0.1%, without gaps) differences in nrSSU, 2/750 bp (0.3%, without gaps) differences in nrLSU, and identical *tef1-α* sequences. Morphologically, the collection fits well with the description of *Pse. puerense* (MHZU 22-0153, holotype) in having solitary, immersed ascomata, 8-spored, cylindric-clavate to obovoid asci, and overlapping, hyaline, fusiform, septate ascospores constricted at the septa (Manawasinghe et al. 2025). Therefore, based on morphological and phylogenetic analyses, the collection is introduced here as a new host record of *Pse. puerense*, collected from *Macadamia* sp. in China.

#### Pseudochaetosphaeronema siamense

Taxon classificationFungiPleosporalesMacrodiplodiopsidaceae

Jayasiri, E.B.G. Jones & K.D. Hyde [as ‘siamensis’], in Jayasiri et al., Mycosphere 10(1): 88 (2019)

4A83EDED-C85D-585D-A04B-F7DA9A22B0B8

648070

Figs 9, 19

##### Description.

***Saprobic*** on a dead branch of *Macadamia* sp. **Sexual morph: *Ascomata*** 255–420 × 380–470 µm (x̄ = 327 × 355 µm, *n* = 10, including ostioles), solitary, immersed to erumpent, scattered, uni-loculate, dark brown to black, globose to subglobose, ostiolate, papillate. ***Ostioles*** up to 50–80 µm wide, neck, cylindrical to papillate. ***Peridium*** 15–50 µm wide, thin-walled, composed of several layers, brown to light brown cells of textura prismatica. ***Hamathecium*** composed of 1.5–3.0 µm wide (x̄ = 2.1 μm, *n* = 40), abundant, dense, narrowly cylindrical to filiform, hyaline, branched, pseudoparaphyses. ***Asci*** 80–130 × 15–25 µm (x̄ = 100 × 17.5 µm, *n* = 40), 4–8-spored, bitunicate, cylindric-clavate obovoid, straight to slightly curved, rounded at apex, pedicellate with club shaped base, with an apical chamber. ***Ascospores*** 23–33 × 7.5–12.5 µm (x̄ = 26.8 × 9.6 µm, *n* = 40), sometimes overlapping, fusiform to broadly fusiform, 1-septate at center, with pointed ends, with wider upper cell, hyaline, containing up to four guttules, smooth-walled, without mucilaginous sheath or appendages. **Asexual morph**: See Jayasiri et al. (2019).

**Figure 19.**
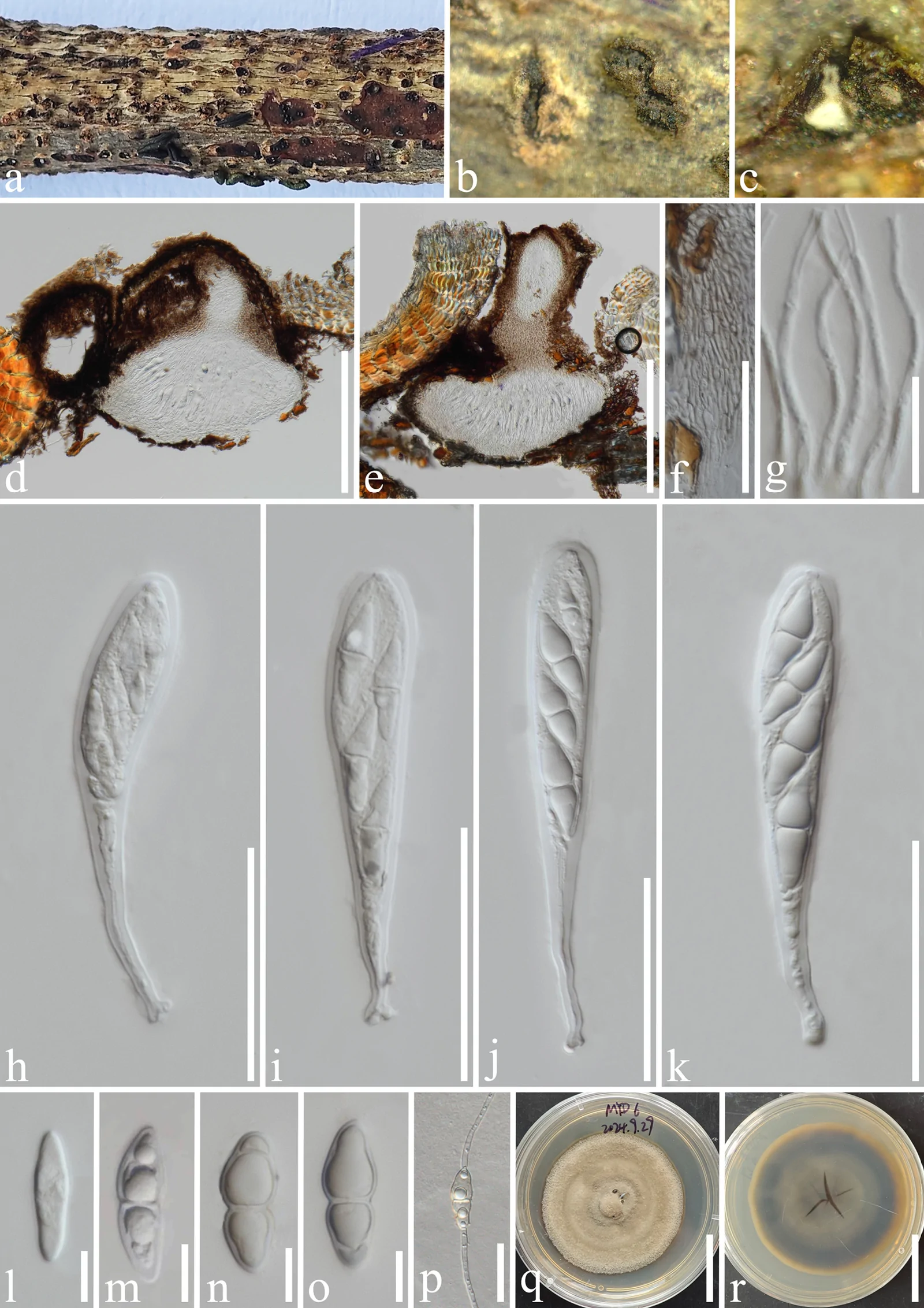
*Pseudochaetosphaeronema siamense* (GMB-W 1573, new host record and country record). **a, b**. Appearance of ascomata on substrate; **c–e**. Vertical sections of ascomata; **f**. Peridium; **g**. Hamathecium; **h–k**. Asci; **l–o**. Ascospores; **p**. Germinated ascospore; **q, r**. Culture on PDA from above (**q**) and below (**r**). Scale bars: 200 μm (**d, e**); 50 μm (**f, h–k**); 30 μm (**p**); 20 μm (**g**); 10 μm (**l–o**); 2 cm (**q, r**).

##### Known hosts.

*Macadamia* sp. (this study), *Tamarind* sp. (Jayasiri et al. 2019).

##### Known distribution.

China (Yunnan Province; this study), Thailand (Jayasiri et al. 2019).

##### Material examined.

China • Yunnan Province, Dehong City, on a dead branch of *Macadamia* sp., April 2024, Xian Zhang, MYD6 (GMB-W 1573); living culture, GMBCC 2460, other living culture GMBCC 2461.

##### Notes.

Jayasiri et al. (2019) introduced *Pseudochaetosphaeronema siamense* from a fallen pod of *Tamarind* sp. (*Fabaceae*) in Thailand, based only on the asexual morph. In the phylogenetic analysis, the isolates GMBCC 2460 and GMBCC 2461 clustered with *Pse. siamense* (MFLUCC 17-2287) with 100% MLBS and 1.00 BYPP statistical support (Fig. 9). Based on nucleotide comparisons, the isolate GMBCC 2460 showed similarity to *Pse. siamense* (MFLUCC 17-2287), with 1/516 bp (0.1%, without gaps) difference in ITS, 1/830 bp (0.1%, without gaps) difference in nrLSU, and 1/1005 bp (0.1%, without gaps) difference in nrSSU. Morphologically, the collection revealed the sexual morph, which is almost identical to that of other species in the genus *Pseudochaetosphaeronema*, with immersed ascomata, bitunicate, fissitunicate asci with rounded ends, and fusiform, 1-septate, guttulate ascospores (Boonmee et al. 2021; Xu et al. 2024). Therefore, based on the phylogenetic analysis, the isolate is introduced here as a new host and country record of *Pse. siamense*, collected from *Macadamia* sp. in China. In addition, this is the first report of *Pseudochaetosphaeronema siamense* associated with *Macadamia* sp.

#### Helminthosporium puerense

Taxon classificationFungiPleosporalesMassarinaceae

L. Lu & Tibpromma, IMA Fungus 16(e144874): 22 (2025)

89E046CB-D5BF-56D8-8C38-D9D4BD48C9C4

901003

Figs 10, 20

##### Holotype.

HKAS 137606

**Figure 20.**
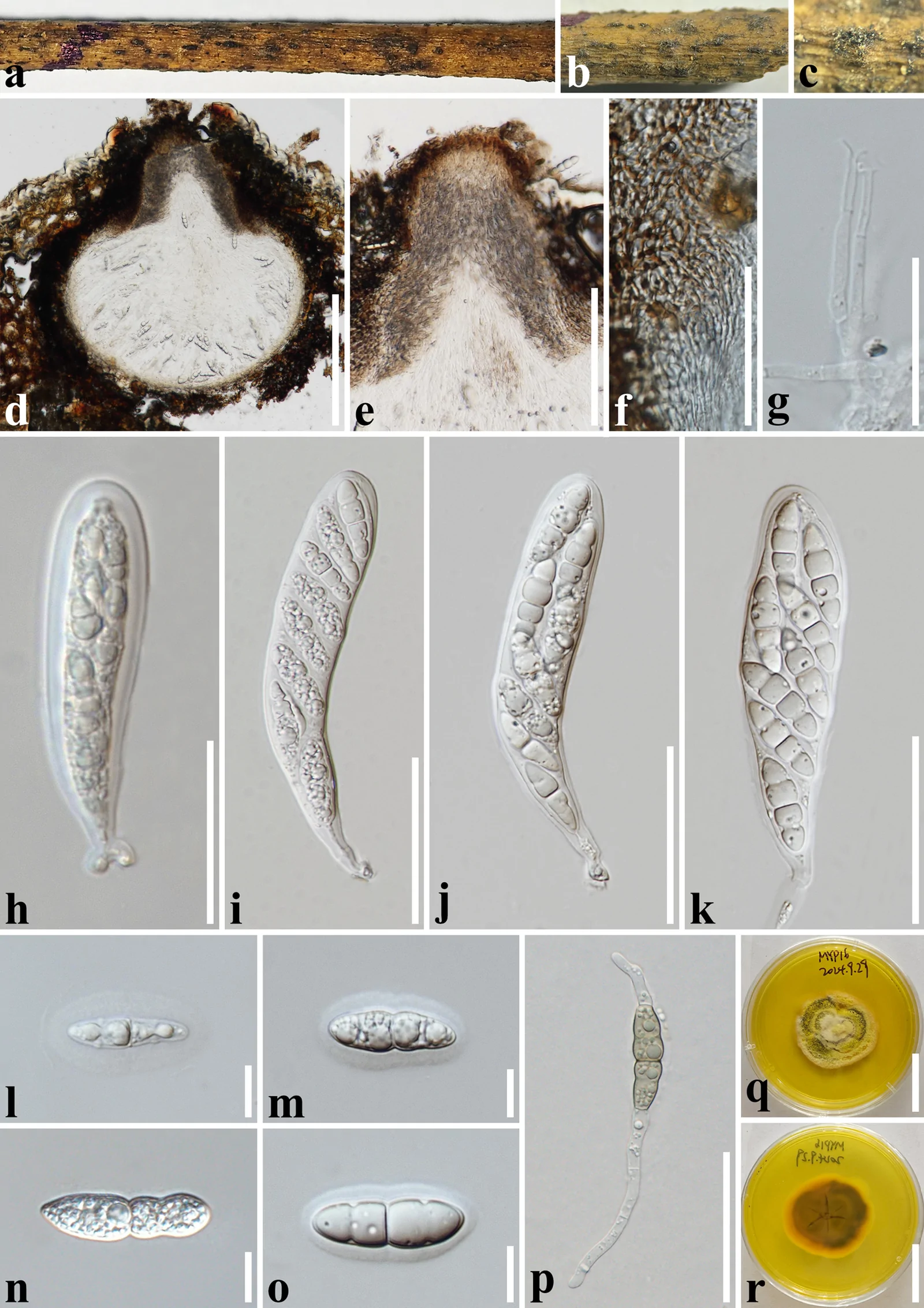
*Helminthosporium puerense* (GMB-W 1574, new host record). **a–c**. Appearance of ascomata on substrate; **d**. Vertical section of ascoma; **e**. Ostiole; **f**. Peridium; **g**. Hamathecium; **h–k**. Asci; **l–o**. Ascospores; **p**. Germinated ascospore; **q, r**. Culture on PDA from above (**q**) and below (**r**). Scale bars: 200 μm (**d**); 100 μm (**e**); 50 μm (**f, i–k**); 30 μm (**g, h**); 10 μm (**l–o**); 2 cm (**q, r**).

##### Description.

***Saprobic*** on dead twigs of *Macadamia* sp. **Sexual morph: *Ascomata*** 250–530 × 330–500 µm (x̄ = 403 × 381 µm, *n* = 10, including ostioles), solitary to scattered, immersed, slightly erumpent, dark spots with black hair on the host surface, globose to subglobose, uniloculate, centrally ostiolate, papillate. ***Ostioles*** neck central, cylindrical to papillate, surrounded by a dark brown clypeus-like structure, without periphyses. ***Peridium*** 35–75 µm thin to thick-walled, composed of hyaline to brown to dark brown cells of textura angularis. ***Hamathecium*** composed of 1.5–3.0 µm wide (x̄ = 2.3 μm, *n* = 40), abundant, dense, narrowly cylindrical to filiform, hyaline, septate, branched, pseudoparaphyses. ***Asci*** 90–160 × 20–30 µm (x̄ = 118 × 23 µm, *n* = 40), 8-spored uni- to biseriate, bitunicate, fissitunicate, clavate, straight to slightly curved, rounded at apex, short-stalked with a club-shaped base, with a narrow apical chamber. ***Ascospores*** 25–32 × 7–11 µm (x̄ = 27.5 × 9 µm, *n* = 40), ellipsoidal to oblong, mostly straight, 1–3-septate with constriction at the septum, hyaline, with subacute to rounded end cells, asymmetric, with wider upper cell, guttulate, smooth-walled, with a thick sheath. **Asexual morph**: Not observed.

##### Culture characteristics.

Ascospores germinating on PDA within 24 h at 28 °C, turning light gray after germination. Colonies on PDA reaching 3.6 cm in diam. after 3 weeks of incubation at 28 °C, with a rough surface; mycelium with filamentous, hairy hyphae, circular, cottony, light yellow from above, surrounded near the center by a yellow to green to light gray ring, and light yellow to brown from the edge to the center; colonies with cracks in the middle.

##### Known hosts.

*Coffea arabica* (Lu et al. 2025a), *Macadamia* sp. (this study).

##### Known distribution.

China (Yunnan Province; Lu et al. 2025a; this study).

##### Materials examined.

China • Yunnan Province, Pu’er City, on dead twigs of *Macadamia* sp., 1 August 2024, Xian Zhang, MYP16 (GMB-W 1574); living culture, GMBCC 2462, • ibid., MYP24 (GMB-W 1575), living culture, GMBCC 2464.

##### Notes.

In the phylogenetic analyses, the isolates GMBCC 2462 and GMBCC 2464 clustered within *Helminthosporium*, sister to *H. puerense* (KUNCC 24-18347), with 82% MLBS and 1.00 BYPP statistical support (Fig. 10). Morphologically, the isolates resemble *H. puerense* (HKAS 137606, holotype) in having immersed ascomata, fissitunicate, bitunicate asci, and 1–3-septate ascospores with wider upper cells. However, the isolates differ from *H. puerense* (HKAS 137606, holotype) by having ascospores with subacute to rounded end cells and a conspicuous sheath and by having hyaline ascospores that turn light gray after germination (Lu et al. 2025a). Based on nucleotide comparisons, the isolates differ from *H. puerense* by 8/549 bp (1.46%, without gaps) in ITS, 2/834 bp (0.2%, without gaps) in nrLSU, no differences in nrSSU, 2/1043 bp (0.2%, without gaps) in *rpb2*, and 3/744 bp (0.4%, without gaps) in *tef1-α*. Therefore, *Helminthosporium puerense* is introduced here as a new host record, collected from *Macadamia* sp. in China.

#### Helminthosporium nanjingense

Taxon classificationFungiPleosporalesMassarinaceae

Meng Zhang, Xiao J. Wang & H.Y. Wu, Mycotaxon 127: 2 (2014)

C2579CD2-F5A1-5A2D-AA40-CA79AE10B934

804655

Figs 10, 21

##### Description.

***Saprobic*** on dead twigs of *Macadamia* sp. **Sexual morph: *Ascomata*** 200–310 µm high × 200–375 µm diam. (x̄ = 251 × 292 µm, *n* = 15, including ostioles), solitary to scattered, immersed, visible as dark spots with a small amount of black hairs on host surface, subglobose to pyriform, uniloculate, ostiolate, with a papilla. ***Ostioles*** neck central, cylindrical to papillate, surrounded by a dark brown clypeus-like structure, without periphyses. ***Peridium*** 25–50 µm thin to thick-walled, composed of hyaline to brown to dark brown cells of textura angularis. ***Hamathecium*** composed of abundant 2–3.5 µm wide (x̄ = 2.5 μm, *n* = 40), hyaline, septate, branched, dense, narrowly cylindrical to filiform, pseudoparaphyses. ***Asci*** 75–130 × 17–27 µm (x̄ = 99.6 × 20.5 µm, *n* = 30), 2–8-spored, bitunicate, fissitunicate, straight to slightly curved, clavate, rounded at apex, short-stalked, with a narrow apical chamber. ***Ascospores*** 25–33 × 6.5–11 µm (x̄ = 28.7 × 8.3 µm, *n* = 40), oblong-ellipsoidal to fusiform, straight to slight curved, 1–3-septate, strongly constricted at middle septum, slightly or not constricted at both side septa, asymmetric, with wider upper cell, hyaline, with subacute to rounded end cells, smooth-walled, with a sheath on surface, guttulate. **Asexual morph**: See Wang et al. (2014).

**Figure 21.**
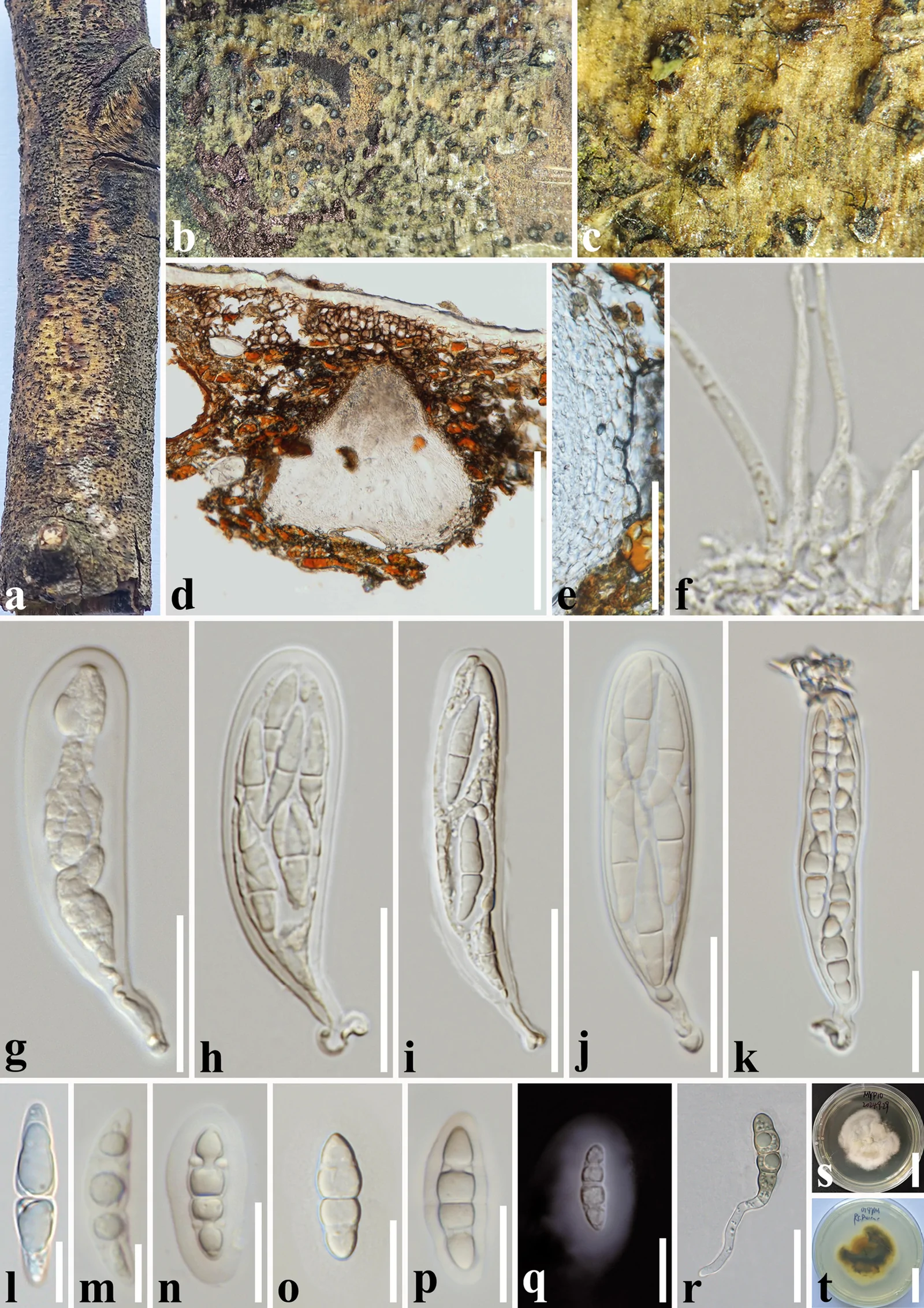
*Helminthosporium nanjingense* (GMB-W 1576, new host record). **a–c**. Appearance of ascomata on substrate; **d**. Vertical section of ascoma; **e**. Peridium; **f**. Hamathecium; **g–k**. Asci; **l–q**. Ascospores (**q**: stained with Indian ink); **r**. Germinated ascospore; **s, t**. Culture on PDA from above (**s**) and below (**t**). Scale bars: 200 μm (**d**); 50 μm (**e**); 30 μm (**g–k, r**); 20 μm (**f, n–q**); 10 μm (**l, m**); 2 cm (**s, t**).

##### Culture characteristics.

Ascospores germinating on PDA within 24 h at 28 °C, turning light gray after germination. Colonies on PDA reaching 3.3 cm in diam. after 3 weeks at 28 °C, with a rough surface; mycelium composed of filamentous, hairy hyphae, circular with an irregular margin, cottony, white from above, surrounded near the center by a slightly yellowish to pale cream coloration, and irregularly light yellow to brown from the edge to the center; colonies with light cracks in the middle.

##### Known hosts.

*Macadamia* sp. (this study), on dead branches of an unidentified tree (Wang et al. 2014).

##### Known distribution.

China (Jiangsu Province; Wang et al. 2014, Yunnan Province; this study).

##### Material examined.

China • Yunnan Province, Pu’er City, on dead twigs of *Macadamia* sp., 1 August 2024, Xian Zhang, MYP10 (GMB-W 1576); living culture, GMBCC 2466, other living culture GMBCC 2467.

##### Notes.

In the phylogenetic tree, the isolates GMBCC 2466 and GMBCC 2467 were nested with *Helminthosporium nanjingense* (HHAUF020380) with 89% MLBS and 1.00 BYPP statistical support (Fig. 10). Based on pairwise nucleotide comparisons, the isolates differ from *H. nanjingense* (HHAUF020380) by 4/454 bp (0.9%, without gaps) in ITS. Morphologically, Wang et al. (2014) described the asexual morph of *Helminthosporium nanjingense*. In this study, the isolates exhibited the sexual morph. *Helminthosporium chinense* (HKAS 124017, holotype) has only been recorded from its asexual morph; therefore, morphological comparison with this species was not possible. The isolates can be distinguished from *H. puerense* (HKAS 137606, holotype) by having subglobose to pyriform ascomata, 2–8-spored, straight to slightly curved asci, and ascospores with conspicuous sheaths, whereas *H. puerense* has globose to subglobose ascomata, 2–8-spored asci, ascospores with inconspicuous sheaths, and sometimes long stipes (Lu et al. 2025a). The phylogenetic tree also shows that they are distinct species (Fig. 10). Therefore, based on the phylogenetic analysis, the isolates are introduced as a new host record of *H. nanjingense*, collected from *Macadamia* sp. in China. This is the first report of *Helminthosporium nanjingense* associated with *Macadamia* sp.

### Evaluation of lignocellulitic enzyme production

**Figure 22.**
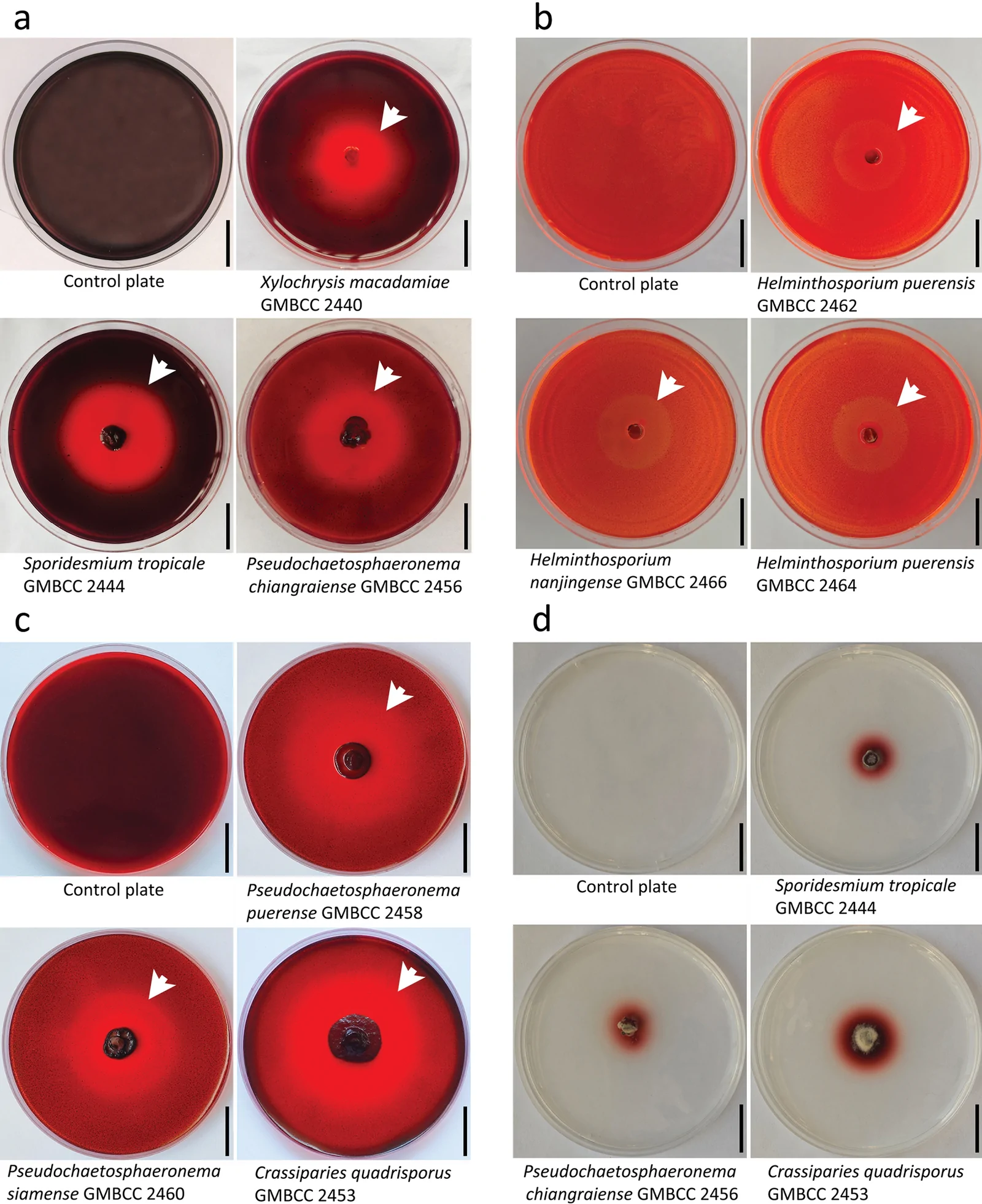
Plate assay for lignocellulolytic enzyme production by saprobic fungi isolated from *Macadamia* sp. in this study. **a**. Xylanase production detected by the formation of clear hydrolysis zones after Congo red staining (indicated by arrowheads); **b**. Cellulase production indicated by the formation of clear hydrolysis zones after Congo red staining (indicated by arrowheads); **c**. Mannanase production indicated by the formation of clear hydrolysis zones after Congo red staining (indicated by arrowheads); **d**. Laccase production indicated by reddish-brown coloration on guaiacol-containing medium. Representative photographs of the three isolates showing the highest enzyme production in each assay are presented. Scale bars: 10 mm.

In this study, 20 pure cultures representing 18 species of saprobic fungi obtained from *Macadamia* specimens collected in China and Thailand were evaluated for their ability to produce lignocellulolytic enzymes, including xylanase, cellulase, mannanase, and laccase. The results showed that the lignocellulolytic enzyme production profiles of 17 fungal isolates varied considerably among the four tested enzymes, whereas three isolates, *Dothiorella lignicola* SDBR-CMU873, *Lasiodiplodia ponkanicola* SDBR-CMU875, and *Lasiodiplodia endophytica* SDBR-CMU876, showed no detectable production of any of the four tested enzymes. A total of 17 isolates representing 15 species exhibited positive xylanase production, 14 isolates representing 12 species exhibited positive cellulase production, and 10 isolates representing nine species exhibited positive mannanase production, as determined by the Congo red assay (Fig. 22). In addition, five isolates representing five species exhibited positive laccase activity, as indicated by the formation of a brownish coloration in the culture medium.

**Figure 23.**
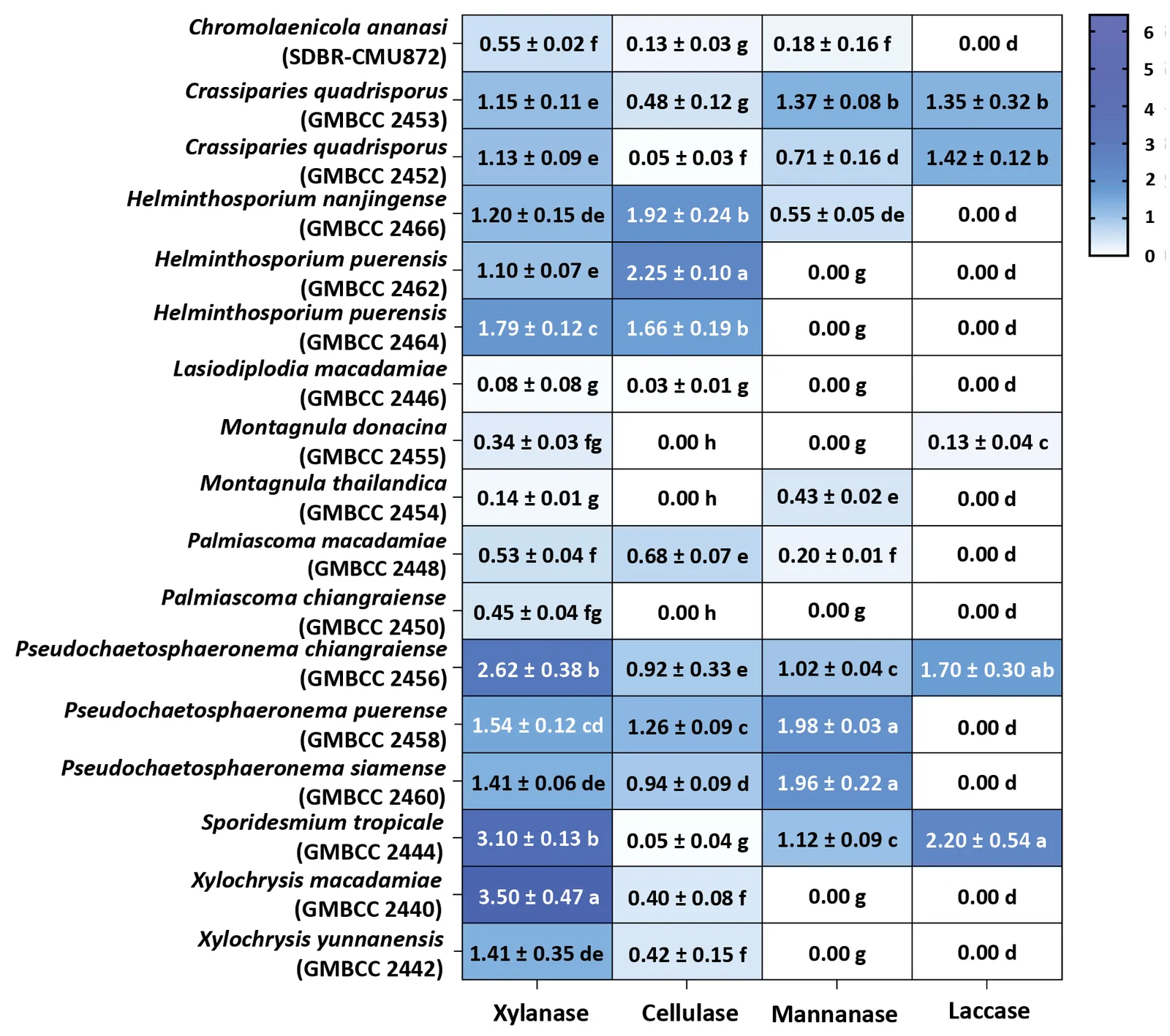
Heat map showing the enzyme potency index for xylanase, cellulase, mannanase, and laccase production by saprobic fungi isolated from macadamia. Data are presented as means ± standard deviation from three replicates. Different letters within each fungal isolate indicate significant differences among enzyme activities (*p* ≤ 0.05), as determined by Duncan’s multiple range test (DMRT).

The enzyme potency index (EPI) was calculated to quantify the relative enzyme-producing ability of the fungal isolates, and the results are presented in Fig. 23. Xylanase production was detected in 17 cultures representing 15 species. The highest xylanase activity was observed in *Xylochrysis macadamiae*GMBCC 2440 (EPI = 3.50), followed by *Sporidesmium tropicale*GMBCC 2444 (EPI = 3.10) and *Pseudochaetosphaeronema chiangraiense*GMBCC 2456 (EPI = 2.62). Cellulase production was detected in 14 cultures representing 12 species, with the highest activity observed in *Helminthosporium puerense*GMBCC 2451 (EPI = 2.25), followed by *H. nanjingense*GMBCC 2466 (EPI = 1.92) and *H. puerense*GMBCC 2450 (EPI = 1.66). Additionally, mannanase production was detected in 10 cultures representing nine species, with the highest activity observed in *Pseudochaetosphaeronema puerense*GMBCC 2458 (EPI = 1.98), followed by *Pse. siamense*GMBCC 2460 (EPI = 1.96) and *Crassiparies quadrisporus*GMBCC 2453 (EPI = 1.37). For laccase production, five isolates representing four species exhibited positive activity, with the highest activity observed in *Sporidesmium tropicale*GMBCC 2444 (EPI = 2.20), followed by *Pseudochaetosphaeronema chiangraiense*GMBCC 2456 (EPI = 1.70) and *Crassiparies quadrisporus*GMBCC 2452 (EPI = 1.42). Overall, four isolates, *Crassiparies quadrisporus*GMBCC 2452 and GMBCC 2453, *Pseudochaetosphaeronema chiangraiense*GMBCC 2456, and *Sporidesmium tropicale*GMBCC 2444, exhibited the ability to produce all four tested lignocellulolytic enzymes.

## Discussion

Saprobic fungi play a crucial role in ecosystem nutrient cycling by decomposing organic matter and releasing nutrients (Sharma and Arora 2015; Corbu et al. 2023; Eliopoulos et al. 2025). However, research on macadamia-associated saprobic fungi remains limited. In this study, saprobic fungi associated with macadamia were collected from Yunnan Province, China, and Chiang Mai Province, Thailand. Based on a combination of multilocus phylogenetic analyses and morphological characteristics, 18 fungal taxa were identified, comprising five newly introduced species (*Dothiorella lignicola*, *Lasiodiplodia macadamiae*, *Palmiascoma macadamiae*, *Xylochrysis macadamiae*, and *X. yunnanensis*) and 13 previously known species (*Chromolaenicola ananasi*, *Crassiparies quadrisporus*, *Helminthosporium nanjingense*, *H. puerense*, *Lasiodiplodia ponkanicola*, *L. endophytica*, *Montagnula donacina*, *M. thailandica*, *Palmiascoma chiangraiense*, *Pseudochaetosphaeronema chiangraiense*, *Pse. puerense*, *Pse. siamense*, and *Sporidesmium tropicale*). The identified fungi belonged to seven families and 10 genera: *Chromolaenicola*, *Crassiparies*, *Dothiorella*, *Helminthosporium*, *Lasiodiplodia*, *Montagnula*, *Palmiascoma*, *Pseudochaetosphaeronema*, *Sporidesmium*, and *Xylochrysis*. Among these, eight genera (*Chromolaenicola*, *Crassiparies*, *Helminthosporium*, *Montagnula*, *Palmiascoma*, *Pseudochaetosphaeronema*, *Sporidesmium*, and *Xylochrysis*) are reported from macadamia for the first time, whereas *Lasiodiplodia* and *Dothiorella* have previously been documented from this host. All 13 known species represent new host records on macadamia. Notably, four species are reported here as new geographical records. Furthermore, the sexual morphs of three species, *Chromolaenicola ananasi*, *Pseudochaetosphaeronema siamense*, and *Helminthosporium nanjingense*, were observed for the first time in this study.

This study shows that saprobic fungi associated with *Macadamia* spp. constitute a diverse source of lignocellulolytic enzymes, although their enzymatic capabilities differ markedly among taxa. Such variation in enzyme production is expected because the degradation of plant biomass requires a complex enzymatic system, and different fungal species have evolved distinct strategies for utilizing lignocellulosic substrates according to their ecological niches and nutritional requirements (Sánchez-Corzo et al. 2021; Bhadra et al. 2022; Khan et al. 2025; Luong et al. 2025;). Among the enzymes evaluated, xylanase was the most frequently detected, followed by cellulase and mannanase, whereas only a few isolates showed laccase activity. These results are consistent with similar trends reported for numerous saprobic ascomycetes, in which xylanase production exceeds cellulase and lignin-degrading enzyme activities during the early stages of plant litter decomposition (Afshari et al. 2025a; Wang et al. 2026). The predominance of xylanase production suggests that hemicellulose degradation represents an important initial step during the decomposition of macadamia tissues. Hemicellulose is generally more accessible and less crystalline than cellulose, allowing fungi to utilize it before attacking the more recalcitrant cellulose and lignin components (Scheller and Ulvskov 2010; Marriott et al. 2016; Petridis and Smith 2018; Sun et al. 2023). The high xylanase activity exhibited by *Xylochrysis macadamiae*GMBCC 2440, *Sporidesmium tropicale*GMBCC 2444, and *Pseudochaetosphaeronema chiangraiense*GMBCC 2456 indicates that these species possess considerable potential for hemicellulose degradation. Likewise, the strong cellulase activities observed in *Helminthosporium puerense* and *H. nanjingense* suggest that members of *Helminthosporium* may play an important role in cellulose decomposition within macadamia ecosystems. Mannanase activity was less common than xylanase and cellulase but was particularly high in *Pseudochaetosphaeronema puerense* and *Pse. siamense*. Laccase production was detected in only five isolates, *Crassiparies quadrisporus*GMBCC 2453, *Crassiparies quadrisporus*GMBCC 2452, *Montagnula donacina*GMBCC 2455, *Pseudochaetosphaeronema chiangraiense*GMBCC 2456, and *Sporidesmium tropicale*GMBCC 2444. Similarly, previous studies have demonstrated that mannanase and laccase activities are generally less prevalent than xylanase and cellulase activities. This pattern may be attributed to the relatively lower abundance of mannan compared with cellulose and xylan in plant cell walls, together with the complex structure of lignin, which is highly resistant to degradation and requires specialized oxidative enzymes for its breakdown (Scheller and Ulvskov 2010; Janusz et al. 2017; Petridis and Smith 2018; Liu et al. 2022; Curry et al. 2023). In contrast, three isolates, *Dothiorella lignicola* SDBR-CMU873, *Lasiodiplodia ponkanicola* SDBR-CMU875, and *Lasiodiplodia endophytica* SDBR-CMU876, showed no detectable activity for any of the four enzymes under the experimental conditions. This lack of detectable activity may reflect enzyme production below the detection limit of the agar plate assay or preferential utilization of readily available simple sugars in the substrate, reducing the need to produce extracellular lignocellulolytic enzymes (Benocci et al. 2017; Saroj and Narasimhulu 2018; Peng et al. 2021). Moreover, extracellular enzyme production depends heavily on substrate composition, nutrient availability, pH, temperature, and incubation time (Loeppmann et al. 2016; Liu et al. 2019; Sharma et al. 2024).

Previous studies on fungi associated with *Macadamia* have primarily focused on pathogenic fungi, with relatively few investigations of endophytic fungi, whereas saprobic fungi have received comparatively little attention (Akinsanmi and Drenth 2013; Wrona et al. 2020; Zhang et al. 2025c). This study expands the current knowledge of saprobic fungi associated with *Macadamia* spp. in China and Thailand by documenting their diversity and evaluating their lignocellulolytic enzyme-producing potential. Most isolates produced at least one lignocellulolytic enzyme, although enzyme production varied considerably among species. Xylanase was the most frequently detected enzyme, followed by cellulase and mannanase, whereas laccase activity was restricted to a limited number of isolates. Notably, *Crassiparies quadrisporus*, *Pseudochaetosphaeronema chiangraiense*, and *Sporidesmium tropicale* exhibited the ability to produce all four tested enzymes, highlighting their potential as promising sources of lignocellulolytic enzymes for biotechnological applications. These findings not only improve understanding of the ecological roles of saprobic fungi in lignocellulose decomposition but also demonstrate that macadamia-associated saprobic fungi represent an underexplored reservoir of industrially relevant enzymes. Further studies should focus on quantitative enzyme assays, optimization of culture conditions, purification and biochemical characterization of the enzymes, and evaluation of their potential for industrial biomass conversion and other biotechnological applications.

## Supplementary Material

XML Treatment for Xylochrysis macadamiae

XML Treatment for Xylochrysis yunnanensis

XML Treatment for Sporidesmium tropicale

XML Treatment for Dothiorella lignicola

XML Treatment for Lasiodiplodia macadamiae

XML Treatment for Lasiodiplodia ponkanicola

XML Treatment for Lasiodiplodia endophytica

XML Treatment for Palmiascoma macadamiae

XML Treatment for Palmiascoma chiangraiense

XML Treatment for Crassiparies quadrisporus

XML Treatment for Chromolaenicola ananasi

XML Treatment for Montagnula donacina

XML Treatment for Montagnula thailandica

XML Treatment for Pseudochaetosphaeronema chiangraiense

XML Treatment for Pseudochaetosphaeronema puerense

XML Treatment for Pseudochaetosphaeronema siamense

XML Treatment for Helminthosporium puerense

XML Treatment for Helminthosporium nanjingense

## Additional information

### Artificial Intelligence (AI) use

The authors accept full responsibility for the content of the manuscript, including the disclosure of any use of AI.

No AI tools were used in the preparation of this manuscript.

### Author ORCIDs

X. Zhang https://orcid.org/0000-0001-6097-8922

S.C. Karunarathna https://orcid.org/0000-0001-7080-0781

S. Tibpromma https://orcid.org/0000-0002-4706-6547

C. Senwanna https://orcid.org/0000-0002-1008-4514

J. Kumla https://orcid.org/0000-0002-3673-6541

T.-Y. Du https://orcid.org/0000-0003-2105-1803

A.M. Elgorban https://orcid.org/0000-0003-3664-7853

A.T. Alfagham https://orcid.org/0000-0001-9008-3679

F.Z. Filimban https://orcid.org/0000-0001-7527-1313

D.-Q. Dai https://orcid.org/0000-0001-8935-8807

N. Suwannarach https://orcid.org/0000-0002-2653-1913

### Data availability

All of the data that support the findings of this study are available in the main text.

## References

[B1] Afshari N, de Farias ARG, Noorabadi MT, Bhunjun CS, Phukhamsakda C, Hyde KD, Alotibi F, Lumyong S (2025a) The crucial roles of ascomycetes in wood litter decomposition: Current knowledge, diversity and function. ScienceAsia 51: 1–13. 10.2306/scienceasia1513-1874.2025.s014

[B2] Afshari N, Noorabadi MT, McKenzie EHC, Pumas C, Bhunjun CS, Jayawardena RS, Gomes de Farias AR, Phukhamsakda C, Jeewon R, Chaharmiri-Dokhaharani S, Suwannarach N, Kumla J, AlOtibi F, Hyde KD, Lumyong S (2025b) Taxonomy and diversity of woody litter microfungi associated with six phylogenetically related host species in Doi Tung national park, Chiang Rai, Thailand. Mycosphere 16: 4783–4935. 10.5943/mycosphere/16/1/36

[B3] Akinsanmi OA, Carvalhais LC (2020) Draft genome of the *Macadamia* husk spot pathogen, *Pseudocercospora macadamiae*. Phytopathology 110: 1503–1506. 10.1094/PHYTO-12-19-0460-A 32343617

[B4] Akinsanmi OA, Drenth A (2013) Pathogenicity and identification of *Botryosphaeriaceae* spp. causing gummosis and dieback in macadamia. Acta Phytopathologica Sinica 43: 21–22.

[B5] Akinsanmi OA, Miles AK, Drenth A (2016a) Fruit abscission in macadamia due to husk spot disease. Acta Horticulturae 1109: 209–214. 10.17660/ActaHortic.2016.1109.34

[B6] Akinsanmi OA, Wang G, Neal JM, Russell D, Drenth A, Topp B (2016b) Variation in susceptibility among macadamia genotypes and species to Phytophthora root decay caused by *Phytophthora cinnamomi*. Crop Protection 87: 37–43. 10.1016/j.cropro.2016.04.021

[B7] Alasalvar C, Huang G, Bolling BW, Jantip PA, Pegg RB, Wong XK, Chang SK, Pelvan E, de Camargo AC, Mandalari G, Hossain A, Shahidi F, Shahidi F (2025) Upcycling commercial nut byproducts for food, nutraceutical, and pharmaceutical applications: A comprehensive review. Food Chemistry 467: e142222. 10.1016/j.foodchem.2024.142222 39626555

[B8] Aptroot A (1995) Redisposition of some species excluded from *Didymosphaeria (Ascomycotina)*. Nova Hedwigia 60: 325–380. 10.1127/nova.hedwigia/60/1995/325

[B9] Apurillo CCS, Phukhamsakda C, Hyde KD, Thiyagaraja V, Jones EBG (2025) New fungal genus, three novel species and one new record from mangroves, with reclassification of *Melanconiella (Melanconiellaceae)* species. MycoKeys 116: 25–52. 10.3897/mycokeys.116.137351 PMC11997611 40235465

[B10] Bao DF, Hyde KD, McKenzie EHC, Jeewon R, Su HY, Nalumpang S, Luo ZL (2021) Biodiversity of lignicolous freshwater hyphomycetes from China and Thailand and description of sixteen species. Journal of Fungi 7: e669. 10.3390/jof7080669 PMC8399276 34436208

[B11] Benatti ALT, Polizeli MLTM (2023) Lignocellulolytic biocatalysts: The main players involved in multiple biotechnological processes for biomass valorization. Microorganisms 11: e162. 10.3390/microorganisms11010162 PMC9864444 36677454

[B12] Benocci T, Aguilar-Pontes MV, Zhou M, Seiboth B, de Vries RP (2017) Regulators of plant biomass degradation in ascomycetous fungi. Biotechnology for Biofuels 10: e152. 10.1186/s13068-017-0841-x PMC5468973 28616076

[B13] Bhadra F, Gupta A, Vasundhara M, Reddy MS (2022) Endophytic fungi: a potential source of industrial enzyme producers. 3 Biotech 12: e86. 10.1007/s13205-022-03145-y PMC8894535 35273898

[B14] Boonmee S, Wanasinghe DN, Calabon MS, Huanraluek N, Chandrasiri SKU, Jones GEB, Rossi W, Leonardi M, Singh SK, Rana S, Singh PN, Maurya DK, Lagashetti AC, Choudhary D, Dai YC, Zhao CL, Mu YH, Yuan HS, He SH, Phookamsak R, Jiang HB, Martín MP, Dueñas M, Telleria MT, Kałucka IL, Jagodziński AM, Liimatainen K, Pereira DS, Phillips AJL, Suwannarach N, Kumla J, Khuna S, Lumyong S, Potter TB, Shivas RG, Sparks AH, Vaghefi N, Abdel-Wahab MA, Abdel-Aziz FA, Li GJ, Lin WF, Singh U, Bhatt RP, Lee HB, Nguyen TTT, Kirk PM, Dutta AK, Acharya K, Sarma VV, Niranjan M, Rajeshkumar KC, Ashtekar N, Lad S, Wijayawardene NN, Bhat DJ, Xu RJ, Wijesinghe SN, Shen HW, Luo ZL, Zhang JY, Sysouphanthong P, Thongklang N, Bao DF, Aluthmuhandiram JVS, Abdollahzadeh J, Javadi A, Dovana F, Usman M, Khalid AN, Dissanayake AJ, Telagathoti A, Probst M, Peintner U, Garrido-Benavent I, Bóna L, Merényi Z, Boros L, Zoltán B, Stielow JB, Jiang N, Tian CM, Shams E, Dehghanizadeh F, Pordel A, Javan-Nikkhah M, Denchev TT, Denchev CM, Kemler M, Begerow D, Deng CY, Harrower E, Bozorov T, Kholmuradova T, Gafforov Y, Abdurazakov A, Xu JC, Mortimer PE, Ren GC, Jeewon R, Maharachchikumbura SSN, Phukhamsakda C, Mapook A, Hyde KD (2021) Fungal diversity notes 1387–1511: taxonomic and phylogenetic contributions on genera and species of fungal taxa. Fungal Diversity 111: 1–335. 10.1007/s13225-021-00489-3 PMC8648402 34899100

[B15] CABI (2026) Crop Protection Compendium. CAB International, Wallingford, UK. CABI Crop Protection Compendium. [accessed 1 September 2026]

[B16] Capella-Gutiérrez S, Silla-Martínez JM, Gabaldón T (2009) TrimAl: A tool for automated alignment trimming in large-scale phylogenetic analyses. Bioinformatics 25: 1972–1973. 10.1093/bioinformatics/btp348 PMC2712344 19505945

[B17] Carbone I, Kohn LM (1999) A method for designing primer sets for speciation studies in filamentous ascomycetes. Mycologia 91: 553–556. 10.1080/00275514.1999.12061051

[B18] Carpenter RJ, Macphail MK, Jordan GJ, Hill RS (2015) Fossil evidence for open, *Proteaceae*-dominated heathlands and fire in the late cretaceous of Australia. American Journal of Botany 102: 2092–2107. 10.3732/ajb.1500343 26643888

[B19] Chukwuma OB, Rafatullah M, Tajarudin HA, Ismail N (2020) Lignocellulolytic enzymes in biotechnological and industrial processes: A review. Sustainability 12: e7282. 10.3390/su12187282

[B20] Corbu VM, Gheorghe-Barbu I, Dumbravă AȘ, Vrâncianu CO, Șesan TE (2023) Current insights in fungal importance-A comprehensive review. Microorganisms 11: e1384. 10.3390/microorganisms11061384 PMC10304223 37374886

[B21] Curry TM, Peña MJ, Urbanowicz BR (2023) An update on xylan structure, biosynthesis, and potential commercial applications. Cell Surface 9: e100101. 10.1016/j.tcsw.2023.100101 PMC9898438 36748082

[B22] de Silva NI, Phillips AJL, Liu J-K, Lumyong S, Hyde KD (2019) Phylogeny and morphology of *Lasiodiplodia* species associated with *Magnolia* forest plants. Scientific Reports 9: e14355. 10.1038/s41598-019-50804-x PMC6778208 31586104

[B23] Denman S, Crous PW, Groenewald JZ, Slippers B, Wingfield BD, Wingfield MJ (2003) Circumscription of *Botryosphaeria* species associated with *Proteaceae* based on morphology and DNA sequence data. Mycologia 95: 294–307. 10.1080/15572536.2004.11833114 21156615

[B24] Dissanayake AJ, Bhunjun CS, Maharachchikumbura SSN, Liu JK (2020) Applied aspects of methods to infer phylogenetic relationships amongst fungi. Mycosphere 11: 2652–2676. 10.5943/mycosphere/11/1/18

[B25] Dou ZP, He W, Zhang Y (2017) Does morphology matter in taxonomy of *Lasiodiplodia*? An answer from *Lasiodiplodia hyalina* sp. nov. Mycosphere 8: 1014–1027. 10.5943/mycosphere/8/2/5

[B26] Du HZ, Chi MF, Wu N, Dissanayake AJ, Liu NG, Cheewangkoon R, Liu JK (2025a) Taxonomic and phylogenetic contributions to *Dothideomycetes* and *Sordariomycetes* associated with medicinal plants in Southwestern China. Mycosphere 16: 179–343. 10.5943/mycosphere/16/2/2

[B27] Du T-Y, Karunarathna SC, Hyde KD, Nilthong S, Mapook A, Dai D-Q, Rajeshkumar KC, Elgorban AM, Han L-S, Wang H-H, Tibpromma S (2025b) New *Aquilariomyces* and *Mangifericomes* species (*Pleosporales*, *Ascomycota*) from *Aquilaria* spp. in China. MycoKeys 112: 103–125. 10.3897/mycokeys.112.139831 PMC11747782 39839667

[B28] Eliopoulos C, Arapoglou D, Haroutounian SA (2025) Bioconversion of agro-industrial byproducts by applying the solid-state fermentation bioprocess to increase their antioxidant potency. Antioxidants 14: e910. 10.3390/antiox14080910 PMC12382885 40867809

[B29] Ellis MB (1971) Dematiaceous *Hyphomycetes*. Commonwealth Mycological Institute. Kew, England, 1–608. 10.1079/9780851986180.0000

[B30] Glass NL, Donaldson GC (1995) Development of primer sets designed for use with the PCR to amplify conserved genes from filamentous ascomycetes. Applied and Environmental Microbiology 61: 1323–1330. 10.1128/aem.61.4.1323-1330.1995 PMC167388 7747954

[B31] Gong L, Ning Y, Chen Y, He X, Wu Z, Yue H (2024) Combined cellular phenotyping and high-throughput sequencing analysis reveals the symbiotic relationships between different types of macadamia root systems and arbuscular mycorrhizal fungi. Frontiers in Sustainable Food Systems 8: e1437577. 10.3389/fsufs.2024.1437577

[B32] Hardner C (2016) *Macadamia* domestication in Hawai’i. Genetic Resources and Crop Evolution 63: 1411–1430. 10.1007/s10722-015-0328-1

[B33] Hongsanan S, Khuna S, Manawasinghe IS, Tibpromma S, Chethana KWT, Xie N, Bagacay JFE, Calabon MS, Chen C, Doilom M, Du HY, Gafforov Y, Huang SK, Li JX, Luangharn T, Luo ZL, Opiña LAD, Pem D, Sadaba RB, Singh R, Tan Q, Tang SM, Wang WP, Wen TC, Xia G, Zhao Q, Bhunjun CS, Cao B, Chen YP, de Silva NI, Dai DQ, Dong W, Du TY, Ferreira-Sá AS, Gao Y, Gui H, Han LS, Han MY, Han XX, Jayawardena RS, Khyaju S, Kumar S, Lei L, LeonardoSilva L, Li H, Li YX, Liao CF, Liu JW, Liu XF, Lu L, Lu WH, Luo M, Maharachchikumbura SSN, Meng QF, Mi LX, Norphanphoun C, Peng XC, Su HL, Tennakoon DS, Thiyagaraja V, Tun ZL, Wijayawardene NN, Xavier-Santos S, Xiong YR, Xu RF, Yadav S, Yang T, Yang YH, Yarasheva M, Zeng XY, Zhang H, Zhang GQ, Zhang X, Zhao HJ, Zhao RL, Zheng DG, Wanasinghe DN, Karunarathna SC (2025) Mycosphere Notes 521–571: A special edition of fungal biodiversity to celebrate Kevin D. Hyde’s 70^th^ birthday and his exceptional contributions to Mycology. Mycosphere 16: 2002–2180. 10.5943/mycosphere/16/2/1

[B34] Htet ZH, Chethana KWT, Manawasinghe IS, Rathnayaka AR, Wijesinghe SN, Win H, Hyde KD, Mapook A (2025) Taxonomy and biological properties of *Ascomycota* on *Bidens Pilosa* and *Chromolaena odorata* in Northern Thailand with a global checklist. Mycosphere 16: 4446–4593. 10.5943/mycosphere/16/1/33

[B35] Hyde KD, Aptroot A, Frohlich J, Taylor JE (1999) Fungi from palms. XLII. *Didymosphaeria* and similar ascomycetes from palms. Nova Hedwigia 69: 449–471. 10.1127/nova.hedwigia/69/1999/449

[B36] Jami F, Slippers B, Wingfield MJ, Gryzenhout M (2012) Five new species of the *Botryosphaeriaceae* from *Acacia karroo* in South Africa. Cryptogamie Mycologie 3: 245–266. 10.7872/crym.v33.iss3.2012.245

[B37] Janusz G, Pawlik A, Sulej J, Swiderska-Burek U, Jarosz-Wilkolazka A, Paszczynski A (2017) Lignin degradation: microorganisms, enzymes involved, genomes analysis and evolution. FEMS Microbiology Reviews 41: 941–962. 10.1093/femsre/fux049 PMC5812493 29088355

[B38] Jayasiri SC, Hyde KD, Jones EBG, McKenzie EHC, Jeewon R, Phillips AJL, Bhat DJ, Wanasinghe DN, Liu JK, Lu YZ, Kang JC, Xu J, Karunarathna SC (2019) Diversity, morphology and molecular phylogeny of *Dothideomycetes* on decaying wild seed pods and fruits. Mycosphere 10: 1–186. 10.5943/mycosphere/10/1/1

[B39] Jeff-Ego OS, Akinsanmi OA (2019) *Botryosphaeriaceae* causing branch dieback and tree death of macadamia in Australia. Australasian Plant Pathology 48: 59–64. 10.1007/s13313-018-0604-6

[B40] Johnston A (1960) A supplement to a host list of plant diseases in Malaya. Mycological Papers 77: 1–30.

[B41] Kalim B, Ali NM (2016) Optimization of fermentation media and growth conditions for microbial xylanase production. Biotech 6: e122. 10.1007/s13205-016-0445-3 PMC4909030 28330199

[B42] Kasana RC, Salwan R, Dhar H, Dutt S, Gulati A (2008) A rapid and easy method for the detection of microbial cellulases on agar plates using gram’s iodine. Current Microbiology 57: 503–507. 10.1007/s00284-008-9276-8 18810533

[B43] Khan J, Drenth A, Akinsanmi F (2025) Mycobiome associated with macadamia plants at different growth stages. Frontiers in Agronomy 7: e1696145. 10.3389/fagro.2025.1696145

[B44] Kumla J, Suwannarach N, Sujarit K, Penkhrue W, Kakumyan P, Jatuwong K, Vadthanarat S, Lumyong S (2020) Cultivation of mushrooms and their lignocellulolytic enzyme production through the utilization of agro-industrial waste. Molecules 25: 2811. 10.3390/molecules25122811 PMC7355594 32570772

[B45] Larsson A (2014) AliView: A fast and lightweight alignment viewer and editor for large datasets. Bioinformatics 30: 3276–3278. 10.1093/bioinformatics/btu531 PMC4221126 25095880

[B46] Lewis JA, Lumsden RD, Locke JC (1996) Biocontrol of damping-off diseases caused by *Rhizoctonia solani* and *Pythium ultimum* with alginate prills of *Gliocladium virens*, *Trichoderma hamatum* and various food bases. Biocontrol Science and Technology 6: 163–174. 10.1080/09583159650039368

[B47] Li GJ, Hyde KD, Zhao RL, Hongsanan S, Abdel-Aziz FA, Abdel-Wahab MA, Alvarado P, Alves-Silva G, Ammirati JF, Ariyawansa HA, Baghela A, Bahkali AH, Beug M, Bhat DJ, Bojantchev D, Boonpratuang T, Bulgakov TS, Camporesi E, Boro MC, Ceska O, Chakraborty D, Chen JJ, Chethana KWT, Chomnunti P, Consiglio G, Cui BK, Dai DQ, Dai YC, Daranagama DA, Das K, Dayarathne MC, de Crop E, de Oliveira RJV, de Souza CAF, de Souza JI, Dentinger BTM, Dissanayake AJ, Doilom M, Drechsler-Santos ER, Ghobad-Nejhad M, Gilmore SP, Góes-Neto A, Gorczak M, Haitjema CH, Hapuarachchi KK, Hashimoto A, He MQ, Henske JK, Hirayama K, Iribarren MJ, Jayasiri SC, Jayawardena RS, Jeon SJ, Jerônimo GH, Jesus AL, Jones EBG, Kang JC, Karunarathna SC, Kirk PM, Konta S, Kuhnert E, Langer E, Lee HS, Lee HB, Li WJ, Li XH, Liimatainen K, Lima DX, Lin CG, Liu JK, Liu XZ, Liu ZY, Luangsa-ard JJ, Lücking R, Lumbsch HT, Lumyong S, Leaño EM, Marano AV, Matsumura M, McKenzie EHC, Mongkolsamrit S, Mortimer PE, Nguyen TTT, Niskanen T, Norphanphoun C, O’Malley MA, Parnmen S, Pawłowska J, Perera RH, Phookamsak R, Phukhamsakda C, Pires-Zottarelli CLA, Raspé O, Reck MA, Rocha SCO, de Santiago ALCMA, Senanayake IC, Setti L, Shang QJ, Singh SK, Sir EB, Solomon KV, Song J, Srikitikulchai P, Stadler M, Suetrong S, Takahashi H, Takahashi T, Tanaka K, Tang LP, Thambugala KM, Thanakitpipattana D, Theodorou MK, Thongbai B, Thummarukcharoen T, Tian Q, Tibpromma S, Verbeken A, Vizzini A, Vlasák J, Voigt K, Wanasinghe DN, Wang Y, Weerakoon G, Wen HA, Wen TC, Wijayawardene NN, Wongkanoun S, Wrzosek M, Xiao YP, Xu JC, Yan JY, Yang J, Da Yang S, Hu Y, Zhang JF, Zhao J, Zhou LW, Peršoh D, Phillips AJL, Maharachchikumbura SSN (2016) Fungal diversity notes 253–366: taxonomic and phylogenetic contributions to fungal taxa. Fungal Diversity 78: 1–237. 10.1007/s13225-016-0366-9

[B48] Li WL, Liang RR, Dissanayake AJ, Liu JK (2023) Mycosphere Notes 413–448: *Dothideomycetes* associated with woody oil plants in China. Mycosphere 14: 1436–1529. 10.5943/mycosphere/14/1/16

[B49] Li X, Leng J, Yu L, Bai H, Li X, Wisniewski M, Liu J, Sui Y (2022) Efficacy of the biocontrol agent *Trichoderma hamatum* against *Lasiodiplodia theobromae* on macadamia. Frontiers in Microbiology 13: e994422. 10.3389/fmicb.2022.994422 PMC9470996 36118222

[B50] Liao M-G, Luo X-X, Xia J-W, Hu Y-F, Zhang X-G, Zhang L-H, Zhang X-P, Xu Z-H, Ma J (2025) Six novel species of *Distoseptispora* (*Distoseptisporaceae*, *Distoseptisporales*) and *Helminthosporium* (*Massarinaceae*, *Pleosporales*) isolated from terrestrial habitats in Southern China. Journal of Fungi 11: e494. 10.3390/jof11070494 PMC12295993 40985377

[B51] Liddle RL, Akinsanmi OA, Galea VJ (2018) Non-host specificity of *Botryosphaeriaceae* on macadamia and blueberry. Australasian Plant Pathology 48: 65–73. 10.1007/s13313-018-0600-x

[B52] Liu D, Qian J, Xu J, Carpita NC, McCann MC, Yang H (2022) Pectin-associated mannans and xylans play distinct roles in cell-cell adhesion in pine and poplar wood. Industrial Crops and Products 184: e115054. 10.1016/j.indcrop.2022.115054

[B53] Liu J-K, Phookamsak R, Doilom M, Wikee S, Li Y-M, Ariyawansha H, Boonmee S, Chomnunti P, Dai D-Q, Bhat JD, Romero AI, Zhuang W-Y, Monkai J, Jones EBG, Chukeatirote E, Ko TWK, Zhao Y-C, Wang Y, Hyde KD (2012) Towards a natural classification of *Botryosphaeriales*. Fungal Diversity 57: 149–210. 10.1007/s13225-012-0207-4

[B54] Liu J-K, Hyde KD, Jones EB, Ariyawansa HA, Bhat JD, Boonmee S, Maharachchikumbura SS, Mckenzie EH, Phookamsak R, Phukhamsakda C, Shenoy BD, Abdel-Wahab MA, Buyck B, Chen J, Chethana KW, Singtripop C, Dai DQ, Dai YC, Daranagama DA, Dissanayake AJ, Doilom M, Dsouza MJ, Fan XL, Goonasekara ID, Hirayama K, Hongsanan S, Jayasiri SC, Jayawardena RS, Karunarathana SC, Li WJ, Mapook A, Norphanphoun C, Pang KL, Perera RH, Peroh D, Pinruan U, Senanayake IC, Somrithipol S, Suetrong S, Tanaka K, Thambugala KM, Tian Q, Tibpromma S, Udayanga D, Wuayawardene NN, Wanasinghe D, Wisitrassameewong K, Zeng XY, Abdel-Aziz FA, Adamk S, Bahkali AH, Boonyuen N, Bulgakov T, Callac P, Chomnunti P, Greiner K, Hashimoto A, Hofstetter V, Kang JC, Lewis D, Li XL, Liu X, Liu ZY, Matsumura M, Mortimer PE, Rambold G, Randrianjohany E, Sato G, Sriindrasutdhi V, Tian CM, Verbeken A, von Brackel W, Wang Y, Wen TC, Xu JC, Yan JY, Zhao RL, Camporesi E (2015) Fungal diversity notes 1–110: taxonomic and phylogenetic contributions to fungal species. Fungal Diversity 72: 1–197. 10.1007/s13225-015-0324-y

[B55] Liu Q, Xu X, Wang H, Blagodatskaya E, Kuzyakov Y (2019) Dominant extracellular enzymes in priming of SOM decomposition depend on temperature. Geoderma 343: 187–195. 10.1016/j.geoderma.2019.02.006

[B56] Liu YJ, Whelen S, Hall BD (1999) Phylogenetic relationships among ascomycetes: evidence from an RNA polymerase II subunit. Molecular Biology and Evolution 16: 1799–1808. 10.1093/oxfordjournals.molbev.a026092 10605121

[B57] Loeppmann S, Blagodatskaya E, Pausch J, Kuzyakov Y (2016) Substrate quality affects kinetics and catalytic efficiency of exo-enzymes in rhizosphere and detritusphere. Soil Biology and Biochemistry 92: 111–118. 10.1016/j.soilbio.2015.09.020

[B58] Lu L, Karunarathna SC, Dai D-Q, Xiong Y-R, Suwannarach N, Stephenson SL, Elgorban AM, Al-Rejaie S, Jayawardena RS, Tibpromma S (2022) Description of four novel species in *Pleosporales* associated with coffee in Yunnan, China. Journal of Fungi 8: e1113. 10.3390/jof8101113 PMC9605522 36294678

[B59] Lu L, Karunarathna SC, Rajeshkumar KC, Elgorban AM, Jayawardena RS, Hongsanan S, Suwannarach N, Kumla J, Xiong Y-R, Hyde KD, Han M-Y, Zheng D-G, Li Q, Dai D-Q, Tibpromma S (2025a) Unveiling fungal diversity associated with coffee trees in China using a polyphasic approach and a global review of coffee saprobic fungi. IMA Fungus 16: e144874. 10.3897/imafungus.16.144874 PMC11915015 40104159

[B60] Lu WH, Alfagham AT, Tibpromma S, Suwannarach N, Kumla J, Dai D-Q, Elgorban AM, Chukeatirote E, Karunarathna SC (2025b) Hidden in the daylight: A polyphasic approach reveals two bioluminescent *Mycenaceae* species from Yunnan Province, China (*Basidiomycota*, *Agaricales*). Biodiversity Data Journal 13: e168858. 10.3897/BDJ.13.e168858 PMC12780892 41522478

[B61] Luong TTH, Silar P, Poeaim S, Tangthirasunun N (2025) Isolation, screening wood rot fungi from the tropical forest of Thailand and their lignocellulolytic enzyme production under solid-state fermentation using agricultural waste as substrate. Biomass Conversion and Biorefinery 15: 2915–2931. 10.1007/s13399-023-05129-1

[B62] Manavalan T, Manavalan A, Heese K (2015) Characterization of lignocellulolytic enzymes from white-rot fungi. Current Microbiology 70: 485–498. 10.1007/s00284-014-0743-0 25487116

[B63] Manawasinghe IS, Hyde KD, WanasinghDN, Karunarathna SC, Maharachchikumbura SSN, Samarakoon MC, Voglmayr H, Pang K-L, Chiang MW, Jones EBG, Saxena RK, Kumar A, RajeshkumarKC, Selbmann L, Coleine C, Hu Y, Ainsworth AM, Liimatainen K, Niskanen T, Ralaiveloarisoa A, Arumugam E, Kezo K, Kaliyaperumal M, Gunaseelan S, Dissanayake AJ, Khalid AN, Gajanayake AJ, Flakus A, Armand A, Aptroot A, Rodrigues A, Tsurykau A, López-Villalba Á, de Faria ARG, Sánchez A, Góes-Neto A, Goto BT, de Souza CAF, Chuaseeharonnachai C, Lin C-G, Li C, Denchev CM, Guerra-Mateo D, Tennakoon DS, Wei D-P, Begerow D, Alves E, Drechsler-Santos ER, Sousa ES, de Medeiros EV, Langer E, ZhangF, de Souza FA, Magurno F, Barreto GG, Moreno G, Mane G, Alves-Silva G, da Silva GA, Xia G, Shen H-W, Gui H, Senanayake IC, Luangsa-ard JJ, Liu J-W, Liu J-K, Ma J, Lin J-Y, Beserra Jr JEA, Cano-Lira JF, Gené J, Harikrishnan K, Lu L, dos Santos LA, Xu L, Lacerda LT, Gusmão LFP, Cáceres MES, Câmara MPS, de Barros-Barreto MBB, CalabonMS, Kukwa M, Kemler M, de Melo MP, Ghobad-Nejhad M, Luo M, Ding M, Doilom M, Phonemany M, Usman M, Thongklang N, Boonyuen N, Ashtekar N, Kularathnage ND, Sruthi OP, Kwantong P, Ansil PA, Kooij PW, Zhao Q, Alfenas RF, de Oliveira RJV, Singh R, da Silva RMF, Avchar R, Morey R, Sharma R, Xu R-J, da Silveira RMB, Xu R-F, Jayawardena RS, Nanu S, Nuankaew S, Tibpromma S, Boonmie S, Somrithipol S, Varghese S, Moreira SI, Rajwar S, He S-C, Kumar TKA, Denchev TT, Luangharn T, de Oliveira TGL, Du T-Y, Wen T-C, Du T, Wu T, Sri-Indrasutdhi V, Doyle VP, Baulin V, Dong W, Li W-L, Lu W-H, Tian W, dos VieiraWA, von Bracke W, Yu X-D, Zhang X, Liu X-F, Peng X-C, Chen Y-P, Yang Y, Gao Y, Xiong Y-R, Shu Y, Lu Y-Z, Shen Y-M, Zhou Y, ZhangY-X, Zhang W, Luo Z-L, Madushani MA, Cheewangkoon R, Song JG, Xu B (2025) Fungal diversity notes 1818–1918: taxonomic and phylogenetic contributions on genera and species of fungi. Fungal Diversity 130: 1–261. 10.1007/s13225-024-00541-y

[B64] Mao T, Chen X, Ding H, Cheng X, Jiang X (2020) Pepper growth promotion and *Fusarium* wilt biocontrol by *Trichoderma hamatum* MHT1134. Biocontrol Science and Technology 30: 1228–1243. 10.1080/09583157.2020.1803212

[B65] Mapook A, Hyde KD, McKenzie EHC, Jones EBG, Bhat DJ, Jeewon R, Stadler M, Samarakoon MC, Malaithong M, Tanunchai B, Buscot F, Wubet T, Purahong W (2020) Taxonomic and phylogenetic contributions to fungi associated with the invasive weed *Chromolaena odorata* (Siam weed). Fungal Diversity 101: 1–175. 10.1007/s13225-020-00444-8

[B66] Marriott PE, Gómez LD, McQueen‐Mason SJ (2016) Unlocking the potential of lignocellulosic biomass through plant science. New Phytologist 209: 1366–1381. 10.1111/nph.13684 26443261

[B67] Miles AK, Akinsanmi OA, Sutherland PW, Aitken EAB, Drenth A (2009) Infection, colonisation and sporulation by *Pseudocercospora macadamiae* on macadamia fruit. Australasian Plant Pathology 38: 36–43. 10.1071/AP08074

[B68] Miller MA, Pfeiffer W, Schwartz T (2010) Creating the CIPRES Science Gateway for inference of large phylogenetic trees. In: 2010 Gateway Computing Environments Workshop (GCE). IEEE Computer Society, New Orleans, LA, USA, 1–8. 10.1109/GCE.2010.5676129

[B69] Mohankumar V, Dann EK, Akinsanmi OA (2022) Diversity and pathogenicity of *Botryosphaeriaceae* associated with macadamia branch dieback in Australia. Plant Disease 106: 2576–2582. 10.1094/PDIS-10-21-2125-RE 35171638

[B70] Mojapelo NA, Seroka NS, Khotseng L (2023) Macadamia nut bio-waste: an agricultural waste with potential to Be used as carbon support material in fuel cell applications. Coatings 13: e1545. 10.3390/coatings13091545

[B71] Monkai J, Wanasinghe DN, Jeewon R, Promputtha I, Phookamsak R (2021) Morphological and phylogenetic characterization of fungi within *Bambusicolaceae*: introducing two new species from the Greater Mekong Subregion. Mycological Progress 20: 721–732. 10.1007/s11557-021-01694-9

[B72] MycoBank (2026) MycoBank. Westerdijk Fungal Biodiversity Institute, Utrecht, The Netherlands. https://www.mycobank.org/ [accessed 12 May 2026]

[B73] Nguyen LT, Schmidt HA, von Haeseler A, Minh BQ (2015) IQ-TREE: A fast and effective stochastic algorithmfor estimating maximum-likelihoodphylogenies. Molecular Biology and Evolution 32: 268–274. 10.1093/molbev/msu300 PMC4271533 25371430

[B74] Nilpanich S, Rukpakavong W, Subsomboon K (2025) Machine learning-based classification of macadamia nut quality using physical features. Journal of Asian Scientific Research 15: 849–863. 10.55493/5003.v15i4.5758

[B75] Nirmalasari I, Santosa DA, Prastowo E (2022) Characterization of lignocellulolytic fungi isolated from cocoa rhizosphere under different cropping patterns. IOP Conference Series: Earth and Environmental Science 980: e012008. 10.1088/1755-1315/980/1/012008

[B76] Nock CJ, Hardner CM, Montenegro JD, Termizi AAA, Hayashi S, Playford J, Edwards D, Batley J (2019) Wild origins of macadamia domestication identified through intraspecific chloroplast genome sequencing. Frontiers in Plant Science 10: e334. 10.3389/fpls.2019.00334 PMC6438079 30949191

[B77] Nylander JAA, Zoology S, Posada D, Mrmodeltest R, Os F (2008) MrModeltest2 v. 2.3 (Program for Selecting DNA Substitution Models Using PAUP). Evolutionary Biology Centre, Uppsala, Sweden.

[B78] O’Donnell K, Cigelnik E (1997) Two divergent intragenomic rDNA ITS2 types within a monophyletic lineage of the fungus *Fusarium* are nonorthologous. Molecular Phylogenetics and Evolution 7: 103–116. 10.1006/mpev.1996.0376 9007025

[B79] Peng M, Khosravi C, Lubbers RJ, Kun RS, Pontes MVA, Battaglia E, Chen C, Dalhuijsen S, Daly P, Lipzen A, Ng V, Yan J, Wang M, Visser J, Grigoriev IV, Mäkelä MR, de Vries RP (2021) CreA-mediated repression of gene expression occurs at low monosaccharide levels during fungal plant biomass conversion in a time and substrate dependent manner. The Cell Surface 7: e100050. 10.1016/j.tcsw.2021.100050 PMC7985698 33778219

[B80] Petridis L, Smith JC (2018) Molecular-level driving forces in lignocellulosic biomass deconstruction for bioenergy. Nature Reviews Chemistry 2: 382–389. 10.1038/s41570-018-0050-6

[B81] Phillips AJL, Alves A, Abdollahzadeh J, Slippers B, Wingfield MJ, Groenewald JZ, Crous PW (2013) The *Botryosphaeriaceae*: genera and species known from culture. Studies in Mycology 76: 51–167. 10.3114/sim0021 PMC3825232 24302790

[B82] Pitt WM, Úrbez-Torres JR, Trouillas FP (2014) *Munkovalsaria donacina* from grapevines and Desert Ash in Australia. Mycosphere 5: 656–661. 10.5943/mycosphere/5/5/6

[B83] Prasannath K, Galea VJ, Akinsanmi OA (2020) Characterisation of leaf spots caused by *Neopestalotiopsis clavispora* and *Colletotrichum siamense* in macadamia in Australia. European Journal of Plant Pathology 156: 1219–1225. 10.1007/s10658-020-01962-6

[B84] Prasannath K, Shivas RG, Galea VJ, Akinsanmi OA (2021) *Neopestalotiopsis* Species Associated with Flower Diseases of *Macadamia integrifolia* in Australia. Journal of Fungi 7: 771. 10.3390/jof7090771 PMC8471233 34575809

[B85] Rabeendran N, Jones EE, Moot DJ, Stewart A (2006) Biocontrol of *Sclerotinia* lettuce drop by *Coniothyrium minitans* and *Trichoderma hamatum*. Biological Control 39: 352–362. 10.1016/j.biocontrol.2006.06.004

[B86] Rambaut A (2012) FigTree. Version 1.4.0. University of Edinburgh, Edinburgh, UK. http://tree.bio.ed.ac.uk/software/figtree [Accessed 10 January 2026]

[B87] Rathnayaka AR, Chethana KT, Phillips AJ, Jones EG (2022) Two new species of *Botryosphaeriaceae (Botryosphaeriales)* and new host/geographical records. Phytotaxa 564: 8–38. 10.11646/phytotaxa.564.1.2

[B88] Rathnayaka AR, Tennakoon DS, Jones GE, Wanasinghe DN, Bhat DJ, Priyashantha AH, Stephenson SL, Tibpromma S, Karunarathna SC (2025) Significance of precise documentation of hosts and geospatial data of fungal collections, with an emphasis on plant-associated fungi. New Zealand Journal of Botany 63: 462–498. 10.1080/0028825X.2024.2381734

[B89] Réblová M, Stepanek V, Schumacher RK (2014) *Xylochrysis lucida* gen. et sp. nov., a new lignicolous ascomycete (*Sordariomycetidae*) with holoblastic conidiogenesis. Mycologia 106: 564–572. 10.3852/13-266 24871596

[B90] Rehner SA, Buckley E (2005) A *Beauveria* phylogeny inferred from nuclear ITS and EF1-α sequences: Evidence for cryptic diversification and links to *Cordyceps* teleomorphs. Mycologia 97: 84–98. 10.3852/mycologia.97.1.84 16389960

[B91] Ren GC, Wanasinghe DN, de Farias ARG, Hyde KD, Yasanthika E, Xu JC, Balasuriya A, Chethana KWT, Gui H (2022) Taxonomic novelties of woody litter fungi (*Didymosphaeriaceae*, *Pleosporales*) from the Greater Mekong Subregion. Biology 11: e1660. 10.3390/biology11111660 PMC9687740 36421373

[B92] Ronquist F, Teslenko M, van der Mark P, Ayres DL, Darling A, Höhna S, Larget B, Liu L, Suchard MA, Huelsenbeck JP (2012) MrBayes 3.2: efficient Bayesian phylogenetic inference and model choice across a large model space. Systematic Biology 61: 539–542. 10.1093/sysbio/sys029 PMC3329765 22357727

[B93] Ruiz RFC, Guarro J, Mayayo E, Decock AC (1997) Notes on conidial fungi. XVI. A new species of *Dendryphiosphaera* and some new records from Cuba. Mycotaxon 67: 9–19. 10.5962/p.415404

[B94] Sánchez C (2009) Lignocellulosic residues: biodegradation and bioconversion by fungi. Biotechnology Advances 27: 185–194. 10.1016/j.biotechadv.2008.11.001 19100826

[B95] Sánchez-Corzo LD, Álvarez-Gutiérrez PE, Meza-Gordillo R, Villalobos-Maldonado JJ, Enciso-Pinto S, Enciso-Sáenz S (2021) Lignocellulolytic enzyme production from wood rot fungi collected in Chiapas, Mexico, and their growth on lignocellulosic material. Journal of Fungi 7: e450. 10.3390/jof7060450 PMC8229410 34198931

[B96] Saroj P, Narasimhulu K (2018) Characterization of thermophilic fungi producing extracellular lignocellulolytic enzymes for lignocellulosic hydrolysis under solid-state fermentation. Bioresources and Bioprocessing 5: 1–14. 10.1186/s40643-018-0216-6

[B97] Scheller HV, Ulvskov P (2010) Hemicelluloses. Annual Review of Plant Biology 61: 263–289. 10.1146/annurev-arplant-042809-112315 20192742

[B98] Senanayake IC, Rathnayaka AR, Marasinghe DS, Calabon MS, Gentekaki E, Lee HB, Hurdeal VG, Pem D, Dissanayake LS, Wijesinghe SN, Bundhun D, Nguyen TT, Goonasekara ID, Abeywickrama PD, Bhunjun CS, Jayawardena RS, Wanasinghe DN, Jeewon R, Bhat DJ, Xiang MM (2020) Morphological approaches in studying fungi: Collection, examination, isolation, sporulation and preservation. Mycosphere 11: 2678–2754. 10.5943/mycosphere/11/1/20

[B99] Senwanna C, Mapook A, Samarakoon MC, Karunarathna A, Wang Y, Tang AMC, Haituk S, Suwannarach N, Hyde KD, Cheewangkoon R (2021) *Ascomycetes* on Para rubber (*Hevea brasiliensis*). Mycosphere 12: 1334–1512. 10.5943/mycosphere/12/1/18

[B100] Senwanna C, Suwannarach N, Phookamsak R, Kumla J, Hongsanan S (2023) New host and geographical records of *Rhytidhysteron* in northern Thailand, and species synonymization. Phytotaxa 601: 157–173. 10.11646/phytotaxa.601.2.3

[B101] Sharma N, Ahlawat YK, Stalin N, Mehmood S, Morya S, Malik AHM, Nellore J, Bhanot D (2024) Microbial enzymes in industrial biotechnology: sources, production, and significant applications of lipases. Journal of Industrial Microbiology and Biotechnology 52: kuaf010. 10.1093/jimb/kuaf010 PMC12094072 40328460

[B102] Sharma RK, Arora DS (2015) Fungal degradation of lignocellulosic residues: an aspect of improved nutritive quality. Critical Reviews in Microbiology 41: 52–60. 10.3109/1040841X.2013.791247 23855359

[B103] Shuai X, Dai T, Chen M, Liang R, Du L, Chen J, Liu C (2022) Comparative study on the extraction of macadamia (*Macadamia integrifolia*) oil using different processing methods. Lebensmittel-Wissenschaft Technologie – Food Science and Technology 154: e112614. 10.1016/j.lwt.2021.112614

[B104] Slippers B, Roux J, Wingfield MJ, van der Walt FJJ, Jami F, Mehl JWM, Marais GJ (2014) Confronting the constraints of morphological taxonomy in the *Botryosphaeriales*. Persoonia 33: 155–168. 10.3767/003158514X684780 PMC4312931 25737598

[B105] Song JY, Yang Y, Yu XD, Dissanayake AJ, Tian Q (2023) *Xylochrysis aquatica* sp. nov. (*Woswasiaceae*) from freshwater habitats in Sichuan Province, China. Phytotaxa 587: 31–38. 10.11646/phytotaxa.587.1.4

[B106] Stamatakis A (2006) RAxML-VI-HPC: Maximum likelihood-based phylogenetic analyses with thousands of taxa and mixed models. Bioinformatics 22(21): 2688–2690. 10.1093/bioinformatics/btl446 16928733

[B107] Stamatakis A (2014) RAxML version 8: A tool for phylogenetic analysis and post-analysis of large phylogenies. Bioinformatics 30(9): 1312–1313. 10.1093/bioinformatics/btu033 PMC3998144 24451623

[B108] Stamatakis A, Hoover P, Rougemont J (2008) A rapid bootstrap algorithm for the RAxML web servers. Systematic Biology 57(5): 758–771. 10.1080/10635150802429642 18853362

[B109] Sun C, Meng X, Sun F, Zhang J, Tu M, Chang J-S, Reungsang A, Xia A, Ragauskas AJ (2023) Advances and perspectives on mass transfer and enzymatic hydrolysis in the enzyme-mediated lignocellulosic biorefinery: A review. Biotechnology Advances 62: e108059. 10.1016/j.biotechadv.2022.108059 36402253

[B110] Sun Y-R, Zhang J-Y, Hyde KD, Wang Y, Jayawardena RS (2023) Morphology and phylogeny reveal three *Montagnula* species from China and Thailand. Plants 12: e738. 10.3390/plants12040738 PMC9961173 36840086

[B111] Sun Y-R, Hyde KD, Liu NG, Jayawardena RS, Wijayawardene NN, Ma J, Zhang Q, Al-Otibi F, Wang Y (2025) Micro-fungi in southern China and northern Thailand: emphasis on medicinal plants. Fungal Diversity: 1–201. 10.1007/s13225-024-00549-4

[B112] Sutton BC (1991) Notes on *Deuteromycetes* III. Sydowia 43: 264–280.

[B113] Tan YP, Shivas RG, Marney TS, Edwards J, Dearnaley J, Jami F, Burgess TI (2019) Australian cultures of *Botryosphaeriaceae* held in Queensland and Victoria plant pathology herbaria revisited. Australasian Plant Pathology 48: 25–34. 10.1007/s13313-018-0559-7

[B114] Tanaka K, Hashimoto A, Matsumura M, Sato T (2017) *Brevicollum*, a new genus in *Neohendersoniaceae*, *Pleosporales*. Mycologia 109: 608–619. 10.1080/00275514.2017.1387432 29144843

[B115] Tao L, Zhang C, Ying Z, Xiong Z, Vaisman HS, Wang C, Shi Z, Shi R (2022) Long-term continuous mono-cropping of *Macadamia integrifolia* greatly affects soil physicochemical properties, rhizospheric bacterial diversity, and metabolite contents. Frontiers in Microbiology 13: e952092. 10.3389/fmicb.2022.952092 PMC9582743 36274682

[B116] Tennakoon DS, Phillips AJL, Phookamsak R, Ariyawansa HA, Bahkali AH, Hyde KD (2017) Sexual morph of *Lasiodiplodia pseudotheobromae* (*Botryosphaeriaceae*, *Botryosphaeriales*, *Dothideomycetes*) from China. Mycosphere 7: 990–1000. 10.5943/mycosphere/si/1b/11

[B117] Thaung MM (2008) Pathologic and taxonomic analysis of leaf spot and tar spot diseases in a tropical dry to wet monsoon ecosystem of lowland Burma. Australasian Plant Pathology 37: 180–197. 10.1071/AP08007

[B118] Tian WH, Jin Y, Liao YC, Faraj TK, Guo XY, Maharachchikumbura SSN (2024a) Phylogenetic insights reveal new taxa in *Thyridariaceae* and *Massarinaceae*. Journal of Fungi 10: e542. 10.3390/jof10080542 PMC11355412 39194868

[B119] Tian XG, Bao DF, Karunarathna SC, Jayawardena RS, Hyde KD, Bhat DJ, Luo ZL, Elgorban AM, Hongsanan S, Rajeshkumar KC, Maharachchikumbura SSN, Suwannarach N, Dawoud TM, Lu YZ, Han JJ, Xiao YP, Du TY, Lu L, Xu RF, Dai DQ, Liu XF, Liu C, Tibpromma S (2024b) Taxonomy and phylogeny of *Ascomycetes* associated with selected economically important monocotyledons in China and Thailand. Mycosphere 15: 1–274. 10.5943/mycosphere/15/1/1

[B120] Tibpromma S, Hyde KD, McKenzie EH, Bhat DJ, Phillips AJ, Wanasinghe DN, Samarakoon MC, Jayawardena RS, Dissanayake AJ, Tennakoon DS, Doilom M, Phookamsak R, Tang AMC, Xu J, Mortimer PE, Promputtha I, Maharachchikumbura SSN, Khan S, Karunarathna SC (2018) Fungal diversity notes 840–928: Microfungi associated with *Pandanaceae*. Fungal Diversity 93: 1–60. 10.1007/s13225-018-0408-6

[B121] Trakunyingcharoen T, Lombard L, Groenewald JZ, Cheewangkoon R, To-Anun C, Crous PW (2015) Caulicolous *Botryosphaeriales* from Thailand. Persoonia 34: 87–99. 10.3767/003158515X685841 PMC4510273 26240447

[B122] Treseder KK, Lennon JT (2015) Fungal traits that drive ecosystem dynamics on land. Microbiology and Molecular Biology Reviews 79: 243–262. 10.1128/MMBR.00001-15 PMC4429240 25971588

[B123] Twiddy DI, Fouché A, Akinsanmi OA, Fourie G (2024) Biology and pathogenicity of fungi causing husk rot of macadamia in South Africa. European Journal of Plant Pathology 170: 501–518. 10.1007/s10658-024-02915-z

[B124] Vaidya G, Lohman DJ, Meier R (2011) SequenceMatrix: Concatenation software for the fast assembly of multi-gene datasets with character set and codon information. Cladistics 27: 171–180. 10.1111/j.1096-0031.2010.00329.x 34875773

[B125] Vilgalys R, Hester M (1990) Rapid genetic identification and mapping of enzymatically amplified ribosomal DNA from several *Cryptococcus* species. Journal of Bacteriology 172: 4238–4246. 10.1128/jb.172.8.4238-4246.1990 PMC213247 2376561

[B126] Wanasinghe DN, Nimalrathna TS, Qin Xian L, Faraj TK, Xu J, Mortimer PE (2024) Taxonomic novelties and global biogeography of *Montagnula* (*Ascomycota*, *Didymosphaeriaceae*). MycoKeys 101: 191–232. 10.3897/mycokeys.101.113259 PMC10820738 38283721

[B127] Wang T, Ma J, Zhong Y, Liu S, Cui W, Liu X, Fan G (2026) Study of fermentation conditions optimization for xylanase production by *Aspergillus tubingensis* FS7Y52 and application in agricultural wastes degradation. Foods 15: e399. 10.3390/foods15020399 PMC12840682 41596999

[B128] Wang XJ, Wu HY, Zhang M (2014) A new species of *Helminthosporium* from Jiangsu, China. Mycotaxon 127: 1–4. 10.5248/127.1

[B129] Wang YZ, Aptroot A, Hyde KD (2004) Revision of the Ascomycete Genus *Amphisphaeria*. Fungal Diversity Press 13: 1–168.

[B130] White T, Bruns T, Lee S, Taylor J (1990) Amplification and direct sequencing of fungal ribosomal RNA genes for phylogenetics. In: Innis MA, Gelfand DH, Sninsky JJ, White TJ (Eds) PCR Protocols: A Guide to Methods and Applications. Academic Press, San Diego, 315–322. 10.1016/B978-0-12-372180-8.50042-1

[B131] Wrona CJ, Mohankumar V, Schoeman MH, Tan YP, Shivas RG, Jeff‐Ego OS, Akinsanmi OA (2020) *Phomopsis* husk rot of macadamia in Australia and South Africa caused by novel *Diaporthe* species. Plant Pathology 69: 911–921. 10.1111/ppa.13170

[B132] Xiao XE, Wang W, Crous PW, Wang HK, Jiao C, Huang F, Pu ZX, Zhu ZR, Li HY (2021) Species of *Botryosphaeriaceae* associated with citrus branch diseases in China. Persoonia 47: 106–135. 10.3767/persoonia.2021.47.03 PMC10486630 37693792

[B133] Xiao Y, Liu JK (2025) *Xylochrysis guttulata* sp. nov., associated with *Camellia sinensis* in Sichuan Province, China. Phytotaxa 698: 35–42. 10.11646/phytotaxa.698.1.4

[B134] Xu R-F, Karunarathna SC, Phukhamsakda C, Dai D-Q, Elgorban AM, Suwannarach N, Kumla J, Wang X-Y, Tibpromma S (2024) Four new species of *Dothideomycetes (Ascomycota)* from Pará Rubber (*Hevea brasiliensis*) in Yunnan Province, China. MycoKeys 103: 71–95. 10.3897/mycokeys.103.117580 PMC10980880 38560534

[B135] Yang J, Maharachchikumbura SSN, Liu JK, Hyde KD, Jones EBG, Al-Sadi AM, Liu ZY (2018) *Pseudostanjehughesia aquitropica* gen. et sp. nov. and *Sporidesmium* sensu lato species from freshwater habitats. Mycological Progress 17: 591–616. 10.1007/s11557-017-1339-4

[B136] Yuliana T, Maharddhika A, Rialita T, Lembong E, Anastassya F, Krama A, Safitri R (2024) Optimization of laccase production from *Marasmius* sp. in a submerged fermentation system. Pakistan Journal of Biological Sciences 27: 283–288. 10.3923/pjbs.2024.283.288

[B137] Zhang J, Xu T, Ge Q (2003) Notes on *Pestalotiopsis* from southern China. Mycotaxon 85: 91–99. 10.5962/p.414697

[B138] Zhang JY, Hyde KD, Bao DF, Hongsanan S, Liu YX, Kang JC, Luo ZL, Liu JK, Lu YZ (2025a) A worldwide checklist and morpho-molecular systematics of fungi associated with pteridophytes. Fungal Diversity 132: 1–273. 10.1007/s13225-025-00554-1

[B139] Zhang Q, Ren Y, Habib K, Lu C, Liu L, Kang J, Shen X, Lin C, Wijayawardene NN, Al-Shwaiman HA, Li Q, Elgorban AM (2025b) Two new species and a new host record of *Hyphomycetes* associated with decaying wood in Yunnan Province, China. MycoKeys 121: 311–328. 10.3897/mycokeys.121.162535.figure3 PMC12421249 40936728

[B140] Zhang X, Karunarathna SC, Tibpromma S, Du TY, Elgorban AM, Lumyong S, Dai DQ, Hyde KD (2024) Morphology and phylogenetic analyses reveal *Neopalmiascoma* gen. nov. (*Bambusicolaceae*, *Pleosporales*) on *Macadamia integrifolia* in Yunnan Province, China. Phytotaxa 633: 230–240. 10.11646/phytotaxa.633.3.3

[B141] Zhang X, Tibpromma S, Karunarathna SC, Du T-Y, Han L-S, Elgorban AM, Kumla J, Senwanna C, Dai D-Q, Suwannarach N, Wang H-H (2025c) Additions to the saprobic fungi (*Ascomycota*) associated with macadamia trees from the Greater Mekong Subregion. MycoKeys 113: 1–29. 10.3897/mycokeys.113.140031 PMC11786193 39897715

[B142] Zhang Y, Feng J, Jiang N, Xu J, Qin C, Xu F, Tian L (2026) *Lasiodiplodia carlesii* sp. nov. (*Botryosphaeriales*, *Botryosphaeriaceae*), a novel pathogen causing canker and dieback on *Castanopsis (Fagaceae)* plantations in China. MycoKeys 137: 81–102. 10.3897/mycokeys.137.184738 PMC13416940 42529214

[B143] Zhao ZZ, Zhao K, Chen HP, Bai X, Zhang L, Liu JK (2018) Terpenoids from the mushroom-associated fungus *Montagnula donacina*. Phytochemistry 147: 21–29. 10.1016/j.phytochem.2017.12.015 29287257

[B144] Zhou J, Li A, Jiang N (2025) Morphology and phylogeny reveal new species and records of *Diplodia*, *Dothiorella*, and *Phaeobotryon* associated with tree cankers in Xizang, China. Journal of Fungi 11: e331. 10.3390/jof11050331 PMC12112754 40422665

[B145] Zhou Y, Manawasinghe IS, He Z, Zhang W, Liu M, Song J, Li S, Fan Z, Yan J (2024) Microfungi associated with peach branch diseases in China. Journal of Fungi 10: e217. 10.3390/jof10030217 PMC10971364 38535225

